# Abstracts of the 16th European Congress of Paediatric and Adolescent Gynaecology †

**DOI:** 10.3390/jcm13247574

**Published:** 2024-12-12

**Authors:** Panagiotis Christopoulos, Anastasia Vatopoulou, Lina Michala, Zuzana Nižňanská, Zoran Stankovic, Evelien Roos, Theodoros Theodoridis, Pandelis Tsimaris, Pallavi Lathe, Angelos Daniilidis, Nikos F. Vlahos, Žana Bumbulienė

**Affiliations:** 1 PAG Clinic, 2nd Department of Obstetrics and Gynecology, Aretaieio Hospital, Medical School, National and Kapodistrian University of Athens, 15772 Athens, Greece; 2 Department of Obstetrics and Gynecology, PAG, Faculty of Medicine, University of Ioannina, 45110 Ioannina, Greece; 3 1st Department of Obstetrics and Gynaecology, Alexandra Hospital, Medical School, National and Kapodistrian University of Athens, 15772 Athens, Greece; 4 1st Department of Obstetrics and Gynecology, School of Medicine, Commenius University, 81499 Bratislava, Slovakia; 5 Department of PAG Surgery and FIGIJ Training Center, Hospital Euromedik, 11000 Belgrade, Serbia; 6 Department of Obstetrics and Gynaecology, Tergooi MC, 10016 Hilversum, The Netherlands; 7 1st Department of Obstetrics and Gynaecology, Papageorgiou Hospital, Aristotle University of Thessaloniki, 54124 Thessaloniki, Greece; 8 Division of PAG, IASO Maternity Hospital, 15123 Athens, Greece; 9 Birmingham Women’s and Children’s NHS Foundation Trust, University of Birmingham, Birmingham B15 2TT, UK; 10 Clinic of Obstetrics and Gynaecology, Institute of Clinical Medicine, Faculty of Medicine, Vilnius University, 01513 Vilniaus, Lithuania

**Keywords:** paediatric, adolescent, gynaecology, congress, abstracts, HellenicPAG, EURAPAG

## Abstract

**Objectives of Paediatric and Adolescent Gynaecology (PAG):** PAG aims to foster a collaborative environment that bridges knowledge from various disciplines to ensure the highest quality of care for children and adolescents with gynaecological issues. The European Association of PAG and HellenicPAG, like all National PAG Societies, support research and education to advance new insights, improve health outcomes, enhance quality of life, and protect future fertility. Additionally, PAG promotes international cooperation by proposing guidelines for good clinical practice in terms of prevention, diagnosis, procedures, and treatment. **Scientific Program and Highlights:** The scientific committee curated a comprehensive program featuring renowned experts and researchers from across Europe. Over four days, participants explored a wide range of topics through lectures, panel discussions, case studies, debates, video presentations, and workshops. The conference attracted professionals from various disciplines, including gynaecology, paediatrics, paediatric endocrinology, psychology, plastic surgery, and paediatric surgery. The collaboration between the European Association of Paediatric and Adolescent Gynaecology and the Hellenic Society of Paediatric and Adolescent Gynaecology resulted in a cutting-edge scientific program. World-renowned experts (Appendix A) presented on all aspects of paediatric and adolescent gynaecology, with a particular focus on emerging topics such as abortion, global rights, transgender care, teenage pregnancy, and more. **Theme—Individualized Care in an Evolving World:** The congress theme aligned perfectly with the evolving landscape of PAG. By presenting high-quality, evidence-based topics, the program aimed to equip the next generation of specialists with the knowledge and skills needed to provide individualized care in a rapidly changing world.

**O01. Use of Gonadotrophin Releasing Hormone Agonists in an Adolescent Patient with an Obstructive Mullerian Anomaly: A Scoping Review**

**N. Warreth, S. McQuillan, C. Osborne, K. Nelson, P. Brain**

Department of Pediatric and Adolescent Gynecology, University of Calgary, Calgary, AB, Canada

**Introduction:** A 16 year old with history of Vacterl Association presented with a 11 cm hematometrocolpos. She was started on a Gonadotrophin releasing hormone (GnRH) agonist, leuprolide acetate for menstrual suppression to allow time for surgical planning. After the initial dose of the GnRH agonist, she developed worsening abdominal pain secondary to an oestrogen flare and bleed. She was subsequently trialed on an aromatase inhibitor, Letrozole, with her second dose of Leuprolide.

**Discussion:** GnRH agonists are used off-label to suppress menstruation in patients with Mullerian anomalies. An abstract at NASPAG 2023, discussed need for hematocolpos drainage in 3 cases until a GnRH was used for suppression. This allows time for surgical planning and determination of surgical readiness for postoperative care including potential stenting/dilation. GnRH agonists cause amenorrhea and prevent further accumulation of blood above the obstruction as the hematocolpos dissolves. In an attempt to minimize an oestrogen flare bleed with a GnRH agonist random start and potential irregular bleeding in the first month post injection, Letrozole 5 mg with the initial dose of the GnRH agonist for five days has been utilized. Letrozole is part of the Aromatase inhibitor family and works by reducing Oestrogen thus minimizing the effect of oestrogen at the level of the uterine lining. This is achieved by inhibiting conversion of androgens to oestrogen. The use of Letrozole is based on one study, where Letrozole was used in conjunction with GnRH in adult women with Fibroids and endometriosis.

**Conclusions:** There is limited evidence of use of letrozole with GnRH agonist in the PAG population, more research is needed on this option for quick suppression. More research is also needed to assess if the mechanism of action is the same in the maturing HPO axis and if any side effects are different for adolescents in comparison to adults.

**O02. To See or Not to See: Hematometra or Not?**

**N. Burger, E. Van den Boogaard, F. Yarde, J. Dekker**

Amsterdam University Medical Center, Amsterdam, The Netherlands

**Introduction and Aims of the Study:** Prepubertal vaginal bleeding can be caused by a variety of etiologies, including trauma, vulvo-vaginitis, foreign body, precocious puberty, hormonal withdrawal, dermatological conditions, malignant masses or sexual abuse. We describe an unusual cause of vaginal bleeding in a 9 year old girl. The aim was to describe the clinical challenges of vaginal bleeding in a prepubertal girl.

**Methods:** Case report.

**Results and Discussion:** A healthy 9 year old girl was referred to our outpatient clinic of Pediatric Gynecology because of vaginal bleeding since two months, without trauma and accompanied by mild abdominal discomfort. She suffered from daily vaginal bleeding, varying from spotting to small blood clots. No vaginal discharge, pruritus, fever or constipation. She did not use medication. Physical examination showed a 1.29 m height girl, weighing 28 kg with Tanner stage 1. Genital examination showed normal prepubertal external genitalia without skin lesions or vaginal discharge. A vulvovaginal sample for bacterial culture was normal. Abdominal ultrasound showed an enlarged uterus with a 2.2 cm hyperechogenic intracavitary mass without blood flow (Figure 1) and normal ovaries. It was concluded to be hematometra. MRI confirmed normal development of internal genitals and hematometra. Blood results were (repeatedly): Hb 7.0 mmol/L; FSH 0.7 IU/L; LH < 0.1 IU/L; estradiol < 20 pml/L. Normal values of infection markers, tumor markers, thyroid function and testosterone. Hysteroscopy was performed under general anesthesia and showed a round, pale pedunculated intracavitary structure. Biopsies showed a thrombus, without signs of malignancy. In a following hysteroscopy the structure was completely removed without significant blood loss. The pathology report showed a benign capillary-venous malformation.

**Conclusions:** If in doubt about the diagnosis hematometra based on ultrasound and MRI, a hysteroscopy can be helpful in correctly diagnosing intracavitary lesions. It is important to raise awareness of intracavitary anomalies in prepubertal girls with vaginal bleeding.

**O03. Surgical Correction for Females with Congenital Adrenal Hyperplasia**

**M. Ardelean, I. Orendi, G. Brandtner, L. Bauer, R. Metzger**

Uniklinikum, Paracelsus Medical University, 5020 Salzburg, Austria

**Aims of the Study**: To evaluate the results of urethroplasty (UP) and genitoplasty (GP) in 46XX patients (pts) with congenital adrenal hyperplasia (CAH) due to 21-hydroxylase deficiency.

**Methods**: We retrospectively evaluate our 14 patients with virilization due to CAH who underwent surgical repair: 12 from 2008 to 2016 and 2 patients in 2023. According to the Prader scale, the degree of virilization corresponded to stage III in 3, to stage IV in 8, and to stage V in 3 patients. We performed the operation in 10 patients when they were under 3 years old, and in another 4 patients when they were 7, 14, 15, and 18 years old.

A one-stage repair (UP&GP) was carried out in 12 patients and a two-stage repair in 2 patients.

Of 12 patients with one-stage surgery, 10 patients underwent “total” correction (urethrovaginoplasty, clitoroplasty and labiaplasty); No clitoroplasty was performed in other 2 patients. In 2 patients with two-stage repair, we performed urethrovaginoplasty and labiaplasty at the ages of 15 and 18 years, respectively. These patients underwent clitoroplasty before the age of 2 years. Urethrovaginoplasty was performed by urogenital mobilization in all patients. All patients receive hormone replacement therapy.

**Results**: Follow-up included clinical and endoscopic examination. Outcome criteria were: appearance of the clitoris, labia, vagina and urinary continence. The cosmetic results on the clitoris and labia are satisfactory in all patients. 12 patients have a physiologically sized vagina; in the other 2 two the vaginal opening is stenotic. All patients are continent.

**Conclusions**: Urogenital mobilization enables urethrovaginoplasty and preserves urinary continence in CAH patients. A “one-stage” correction in infancy leads to good functional and cosmetic results.

**O04. Pelvic Pain and Generalized Persistent Pain Syndromes in People with Mayer-Rokitansky-Kuster-Hauser Syndrome: A Cross-Sectional Survey Study**

**R. Gaikaiwari ^1^, S. Grover ^2,3^, I. Wright ^1,4,5^, A. Battle ^1,6^, N. Drever ^1,7^**

^1^  James Cook University, Cairns, QLD 4870, Australia
^2^  Department of Pediatrics, University of Melbourne, Melbourne, VIC 3052, Australia
^3^  Royal Children’s Hospital, Melbourne, VIC, Australia
^4^  Department of Pediatrics, Cairns Hospital, Cairns, QLD, Australia
^5^  The University of Queensland Centre for Clinical Research, University of Queensland, Brisbane, QLD, Australia
^6^  Northern Clinical School, Prince Charles Hospital, Faculty of Medicine, The University of Queensland, Chermside, QLD, Australia
^7^  Department of Obstetrics and Gynecology, Cairns Hospital, Cairns, QLD, Australia

**Introduction and Aims:** To characterize experiences of pelvic and generalized pain in people with Mayer-Rokitansky-Küster-Hauser Syndrome (MRKH) and to compare with the general female population, and between participants with absent uterus and those with uterine remnants.

**Methods:** This cross-sectional study included adults with MRKH who are affiliated with MRKH Australia. Our survey was distributed through MRKH Australia’s social media channels and website. Generalized pain was assessed using the American College of Rheumatology’s modified diagnostic criteria for fibromyalgia, and pelvic pain prevalence and severity data was collected. Descriptive statistics and chi square comparisons were used.

**Results and Discussion:** Of the 142 responses, 23.9% had a uterine remnant, 68.3% did not, and 7.75% were unsure. Absence of any pelvic pain (34.0%) was more common in the sample cohort than rates previously reported in the general female population (24.8%) (*p* = 0.021). However, the prevalence of moderate to severe chronic pelvic pain in the sample population (38.3%) (*p* < 0.001) and in participants without remnant uteri (39.6%) (*p* < 0.001) was higher than non-menstrual and non-sexual moderate to severe pain reported in the general female population (13.3%). This prompts exploration of multifactorial and nongynecological etiologies of persistent pelvic pain. Clinicians should also consider ovulation as a contributor to cyclical pelvic pain, and pain associated with pelvic floor hypertonicity related to vaginal dilator use. 28.2% of the sample cohort and 26.8% of those without remnant uteri met the generalized pain threshold. Pelvic pain was strongly correlated with generalized persistent pain (*p* = 0.002).

**Conclusions:** This is the first study to investigate pain prevalence amongst people with MRKH. Despite a significantly higher proportion of this population reporting absence of pelvic pain, the findings of this study show a surprisingly higher prevalence of moderate to severe pelvic pain, and generalized pain, when compared to general population data.

**O05. Congenital Anomalies of Genitalia in Adolescence—Peculiarities of Treatment Tactic and Surgery**

**T. Tatarchuk, I. Bachynska, I. Gavrilova, I. Mirochnik**

National Children’s Specialized Hospital “Ohmatdyt”, Kyiv, Ukraine
State Institution “Institute of Pediatrics, Obstetrics and Gynecology named after Academician O.M. Lukyanova of the National Academy of Medical Sciences of Ukraine”, Kyiv, Ukraine

**Introduction:** Management tactics for girls with congenital abnormalities of genitals must be carefully planned, with preoperative management, surgical tactics and postoperative monitoring aimed at prevention of endometriosis, stenosis of the created opening in cases of plastic surgery of the vagina and cervix.

**Aim:** To present our experience in treatment of congenital abnormalities of genitals in adolescents.

**Methods:** We reviewed the charts of 225 girls with congenital anomalies who underwent surgical treatment between 2000 and 2023, 60% were complex uterovaginal anomalies.

**Results:** Hemi-uterus with non-communicative functional horn—U4aC0V0—is a rare pathology, only 8%. This malformation presents with severe dysmenorrhea from menarche, requires surgical treatment—removal of the rudimentary horn by laparoscopy. Histological samples of the removed part often show adenomiosis, thus a concern about endometriosis may occur.

Bicorporeal uterus with hemiobstructed vagina (OHVIRA)—U3C2V2—not very rare case. We have 40% cases. We perform plastic of the obstructed vagina wall to provide blood flow without complications.

Bicorporeal uterus may be accompanied by cervical atresia—U3C3V0, resulting in haematometra and haematocervix. We had 18% of cases. The purpose of surgery is to create an external opening of the cervical canal—a difficult task with high risk of stricture and inflammatory complications.

In 72% of cases, we managed to do it from the vagina in 28% of cases. due to technical difficulties, the uterus was removed with an atretic cervix on one side. The normal uterus and cervix remained intact.

In the case of vaginal aplasia with function uterus we start from the cessation of menstruation and conservative colpoelongation, and then the creation of utero-vaginal anastomosis. If impossible to elongate the vagina, we perform surgical treatment in two stages 1-creation of a neo-vagina using oxiginated cellulose and after epithelization 2-creating of utero-vaginal anastomosis. Use of protectors in the postoperative process prevents stenosis of formed openings and to preserve menstrual and reproductive functions. We also use conservative colpoelongation firstly in cases of MRKH syndrome with efficiency 60%.

**Conclusions:**

1. Treatment of patients with congenital genital abnormalities should be carried out in specialized departments with relevant experience.
2. Timely and correct diagnosis, management tactics for both operative treatment and the postoperative period reduce risk of complications and preserve reproductive potential.

**O06. A Differentiated Approach to the Management of Patients with Ohvira Syndrome**

**Z. Batyrova, E. Uvarova, V. Chuprynin, Z. Kumykova, D. Kruglyak, F. Mamedova, P. Kulabuchova, A. Asaturova**

FSBI National Medical Research Center for Obstetrics, Gynecology and Perinatology Named After Academician V.I. Kulako, Moscow, Russia

**Introduction:** OHVIRA is obstruction of hemivagina and ipsilateral renal anomaly; among all malformations, 0.16–10% occur with a frequency of 1:2100 or 1:28,000 live-born girls. The etiological causes are unclear and practically unexplored; Diagnosis is usually delayed due to the presence of relatively regular menstrual function, leading to unjustified surgical interventions.

**Aim of the Study:** To analyze the clinical and anamnestic features of patients with OHVIRA.

**Methods:** 97 patients with OHVIRA aged 10–17 y who were treated at the pediatric and adolescent gynecology department from 2021–2023. Clinical and anamnestic data were studied.

**Results:** Urinary system defect was usually detected before hospitalization in 90.7%, ipsilateral renal aplasia in 78.4% (more often on the right), dysplasia/polycystic kidney 12.4%. From the moment of detection the anomaly of the urinary to a genital passed 6 y. The age of menarche 12 y. Periods continued for 6 days after 28–30, characterized by dysmenorrhea—75.3%. Irregularity of the cycle in 25.8%. Prior to hospitalization 57.7% had experience of previous examination and treatment, due to suspicion of acute surgical pathology 30.9%. 27.8% had a history of surgical treatment. Partial obstruction—the presence of fistulous communication between the hemivagina in 30.9%, unfortunately at the prehospital stage only in 2.1%. It should be noted that patients with partial obstruction were more likely to notice an earlier onset of dysmenorrhea, every second indicated the disappearance of pain after an episode of uterine bleeding, and 43.3% complained of prolonged pathological discharge.

**Conclusions:** The presence of abnormality of the urinary system in a girl, regardless of age, requires a mandatory examination by a gynecologist. The presence of early onset dysmenorrhea with a tendency to progression and/or prolonged pathological discharge, including recurrent episodes of uterine bleeding, is an indication for in-depth follow-up examination in order to exclude genital malformation.

**O07. Ovarian Torsion in Pre-Pubertal Girls: Retrospective Study in 30 French Tertiary Centers**

**C. Defert, A. Haffreingue, T. Deleforterie, P. Verot, A. Scalabre, E. Haraux, F. Schmitt, C. Messelod, O. Hild, A. Olland, A. Belgacem, M. Bourezma, Y. Bonnin, M. Bousquet, M. Zislin, A. Lubet, A. Bourg, P. Clermidi, M. Glenisson, C. Lefébure, H. François-Coridon, A. Cartault, A. Arnaud**

University Hospital of Nantes, Nantes, France

The diagnosis of ovarian torsion is made with delay among pre-pubertal girls and the management after the first surgery is poorly codified. We interested in the French experience during the last two decades.

Retrospective study in French pediatric surgery centers from January 2000 to December 2022 concerning girls who presented with at least 1 episode of ovarian torsion in the prepubertal period. Infants under 2 years of age were excluded. Data collected included patient history, symptomatology, biological and imaging assessment at first episode, emergency surgery, follow-up including relapse.

Thirty centers participated with 503 girls included. None had a history of ovarian transposition. The girls had a mean age of 7.9 years (±2.8) and a median age of 8.3 years (Q1 5.97; Q3 10.12) at the time of the first episode of torsion. Nausea or vomiting was present in 71% of cases. Fever was present in 23% of cases and was more displayed 24 h after the onset of abdominal pain (*p* < 0.0001). Surgery was performed by laparoscopy (71%), laparotomy (15%) or laparoscopy converted to laparotomy (71 cases, 14%). Mean follow-up was 2.1 (±2.61) years. Sixty-seven girls (13% of the cohort) were referred to the endocrinologist after the first episode of torsion. 42/142 (30%) of girls with oversized ovaries were referred to the endocrinologist. There was at least one recurrence in 75/503 girls (17.5%). Among them: 10 (7.5%) had an ovarian mass and 42 (55%) oversized ovaries.

Symptoms of ovarian torsion in pre-pubertal girls are not specific and clinicians should be aware of this insidious presentation in this group. These girls should be systematically referred to endocrinologists.

**O08. Ovarian Immature Teratoma: A Case Report**

**S. Vodopivec ^1,2^, A. Štolfa ^1,2^, T. Kunič Pirš ^1,2^, M. Jakimovska Stefanovska ^1,2^**

^1^  University Medical Centre Ljubljana, Ljubljana, Slovenia
^2^  Medical Faculty, University of Ljubljana, Ljubljana, Slovenia

**Introduction:** Immature teratoma is a malignant germ cell tumor, characterized by presence of immature neuroepithelium. It mostly affects adolescents and is clinically presented as a pelvic tumor mass.

**Case Report:** A nine-year-old previously healthy girl presented with obstipation and enlarged abdomen that has become prominent within ten days before presentation. She didn’t have pain, fever or other complaints. Clinical examination showed symmetrically distended abdomen with no signs of peritonitis. Percussion was dull, peristalsis present. Abdominal ultrasound and MRI showed well-defined, septate cystic formation with vascularized solid inclusions and macroscopic fat tissue inclusions, measuring 14 × 21 × 24 cm with no infiltration of other organs. Left ovary was normal, the right ovary could not be differentiated. The origin of the tumor could not be precisely defined. Laboratory results showed anemia, raised alpha fetoprotein (31.3 kU/L) and Ca125 (333 kU/L). Chest X-ray was normal. We performed median laparotomy and revealed torquate tumor, originating from the right adnexa and miliary plaques on omentum and peritoneum in cavum Douglasi. Right side adnexectomy, partial omentectomy, peritoneal biopsies were done. Histological examination of the tumor showed 24 cm big high-grade immature teratoma inside of the right ovary, and free ovarian surface. Right fallopian tube was normal. Omental and peritoneal biopsies showed glioblastosis without immature neural elements. Cytologic examination of free peritoneal fluid was negative for malignant cells. Eight days after surgery alpha fetoprotein and Ca125 values were in normal range. Tumor was staged as IA. Tumor board concluded that surgical treatment is finished, adjuvant treatment was not required. The patient will be followed by a pediatric gynecologist and oncologist.

**Conclusions:** Immature teratoma in girls is rare. Diagnosis is made by ultrasound and magnetic resonance imaging. Conservative surgical treatment with complete resection of the tumor is the golden standard.

**O09. Primary Ovarian Insufficiency (Poi) and Autoimmune Diseases: A Systematic Review**

**J. Shaqollari ^1^, C. Makedona ^1^ and L. Michala ^2^**

^1^  School of Medicine, NKUA, Athens, Greece
^2^  1st Department of Obstetrics and Gynecology, NKUA, Alexandra Hospital, Athens, Greece

**Introduction:** Primary ovarian insufficiency (POI) is a rare pathology characterized by the reduction or loss of ovarian activity in women younger than 40 years of age. The etiology of POI varies from genetic and autoimmune to iatrogenic causes, whereas there still remains an important portion of idiopathic cases.

**Aims:** This study aimed to identify the most frequent autoimmune diseases connected to POI, in order to assist physicians distinguish the possibility of POI in the context of autoimmunity in early stages.

**Methods:** A systematic review of the literature within the PubMed and ScienceDirect databases was conducted. Studies exploring the co-occurrence of autoimmune diseases and POI were selected and evaluated independently based on predetermined eligibility criteria, while the extraction of data and the assessment of methodological quality were performed concurrently. Only studies concerning humans, published in English and French, were included.

**Results:** According to the preset criteria 48 studies, among which 20 observational, 20 comparative, 3 comparative observational, 2 cross—sectional and 3 clinical trials, as well as a series of 64 case reports were included.

**Discussion:** Based on the aforementioned studies, endocrinopathies (such as Addison’s disease, hypothyroidism, autoimmune polyglandular syndrome), myasthenia gravis, connective tissue disorders (rheumatoid arthritis, systemic lupus erythematosus) and autoimmune oophoritis seem to be associated with POI. It is important to mention that in 7 studies concerning connective tissue disorders, cyclophosphamide treatment was administered, resulting in difficulty in the investigation of the correlation of the disorder with POI. Furthermore, there was no justified association between diabetes mellitus type I and POI.

**Conclusions:** There seems to be a correlation between POI and autoimmune diseases. The pathophysiological pathways uniting the disorders need to be further investigated and clarified, in order for more specific diagnostic tools and biomarkers to be developed.

**O10. Why Am I Tearful and Angry? Premenstrual Syndrome in Adolescent Girls**

**L. Michala ^1,2^, A. Deliveliotou ^2,3^, N. Georgopoulos ^2,4^, S. Roidi ^1,2^, A. Soldatou ^2,5^, P. Tsimaris ^2,6^, A. Vatopoulou ^2,7^**

^1^  National and Kapodistrian University of Athens, Athens, Greece
^2^  Scientific Society PAG-Greece, Athens, Greece
^3^  Paediatric and Adolesecent Gynaecology Department, REA Hospital, Athens, Greece
^4^  Division of Reproductive Endocrinology, Patras Medical School, Patras University Hospital, Rio, Greece
^5^  2nd Department of Paediatrics, National and Kapodistrian University of Athens, Paediatric Hospital of “P&A Kyriakou”, Athens, Greece
^6^  Paediatric and Adolescent Gynaecology Department, Iaso General Hospital, Athens, Greece
^7^  Paediatric and Adolescent Gynaecology, Department of Obstetrics and Gynaecology, University of Ioannina, Ioannina, Greece

**Introduction:** Premenstrual Syndrome (PMS) refers to a cluster of emotional, behavioural and physical symptoms occurring during the luteal phase of the cycle and receding during menstruation. It has an estimated prevalence of 80% and approximately 5–10% of women are affected by Premenstrual Dysphoric Disorder (PMDD), which is a severe psychiatric condition.

**Aim:** The aim of the study was to validate the PMS screening tool PSST-A for usage in Greek adolescents and assess the prevalence of the condition.

**Methods:** The PSST—A questionnaire consists of 19 items, assessing core and secondary PMS symptoms, and the degree of their effect on daily activities.

The Questionnaire was translated and cognitively reviewed, prior to being electronically disseminated anonymously to menstruating adolescents, recruited through specialized clinics in Greece.

**Results:** 128 adolescent girls with a median age of 16 years were included in the study. The questionnaire was reliable (Cronbach α = 0.87).

119 girls (92%) reported at least one premenstrual symptom affecting their daily routine and relations. The most common symptoms were anger/irritability, cravings, and fatigue. 22 girls (17%) were suspected to have PMDD with the most prominent symptom being tearfulness and increased sensitivity to rejection.

48% of respondents reported having no prior knowledge on PMS, whereas 83% stated they would want to have more knowledge on the condition, selecting social media (62%), School (63%) and their doctor (80%) as preferred sources of information.

**Conclusions:** The Greek version of the PSST-A questionnaire is a reliable tool for screening PMS and PMDD in adolescent girls. PMS symptoms were prevalent, and a high proportion of girls may be suffering with PMDD, which requires expert assessment and management. Information through Social Media campaigns, school-based discussions and directed medical consultations will raise awareness on the subject, help girls identify and seek help for PMS symptoms.

**O11. Surgery for Ovarian Dermoid Cyst in the Pediatric and Adolescents Patients, Does the Size of Cyst Matter for Pain?**

**O. Dural, I. Evruke, I. Tas, H. Ulusoy, H. Saygili, C. Yasa, F. Gungor Ugurlucan, S. Akhan**

Department of Obstetrics and Gynecology, Faculty of Medicine, Istanbul University, Istanbul, Turkey

**Introduction:** Dermoid cyst (DC) represents the most frequently diagnosed ovarian neoplasm in the pediatric and adolescent population. Although the definitive indications and timing of surgery have yet to be determined, cysts that are larger than 5 cm, grow rapidly and/or symptomatic usually treated with a surgical management.

**Aims of the Study**: To evaluate indications, approach, and outcomes of the surgeries performed for DC.

**Methods:** We retrospectively reviewed the records of patients aged under 21 who underwent surgical treatment of DC between 2017–2023. Data was collected on demographics, clinical presentation, surgical findings, and follow-up. We also analyzed presenting symptoms among patients with cyst size ≤5 cm (Group-1) and >5 cm (Group-2).

**Results:** Among a total of 92 cases underwent surgical management of adnexal masses, 32 (34%) were performed for DC. The mean age of the patients was 16.6 ± 3.6 (range, 7–21) years. The mean cyst size was 6.1 ± 2.5 cm, two paitent had bilateral tumours. All but three patient (91%) underwent laparoscopic cystectomy (Table 1). Patients in Group-2 (n = 25) was more likely to present with torsion. While only one patient in Group-1 (n = 7) presented with torsion, the other patients required surgical treatment due to chronic pain (<0.001). Among the cases presenting with torsion, while cystectomy was performed at the time of detorsion in 67%, other patients underwent interval ovarian cystectomy.

**Discussion:** Although the timing of surgical treatment of DCs remains controversial, laparoscopic cystectomy is considered a safe and effective approach. Our findings suggest that regardless of size, DCs may present with chronic pelvic/abdominal pain that affects quality of life and may require surgical treatment.

**O12. Recurrent Ovarian Torsion in Pre-Pubertal Girls: Why Perform Oophoropexy and When?**

**C. Defert, S. Faraj, A. Cartault, A. Arnaud**

University Hospital of Nantes, Nantes, France

Ovarian torsion is less frequent in pre-pubertal girls than in teenagers but there is a higher risk of recurrence. Mainly, it occurs without ovarian cyst or tumor. The mechanism of torsion in this population is explained by the enlarged size of the gonad. Literature is poor concerning any age-related size chart of the ovary. We interested in the link between the recurrence of torsion in pre-pubertal girls and oversized ovaries and also the extent in which oophoropexy would be valuable or at least must be discussed.

Retrospective study of 75 pre-pubertal girls from 30 French tertiary centers with recurrent ovarian torsion. We made a statement of the volume of the ovaries on US-scan before and after the first relapse, age at menarche, biological parameters (AMH, testosterone, estradiol, FSH, LH). Oversize ovary was defined as an age-related volume at or beyond the 97th percentile.

The median age of the cohort at the first episode was 7.9 years (±2.8). Table 2 shows the number of relapses reported. Among them, 10 girls had a tumour (excluded for the following analysis. Forty-two had oversized ovaries. Among the 23 girls left, the estimation of the ovarian volume was not possible. The side affected during the first relapse was independent of the side affected during the first episode of torsion (*p* = 0.63). AMH assay(s) (preoperative and/or postoperative) were above the upper laboratory standard in 14/65 girls. Elevated AMH was significantly associated with an increased risk of torsion recurrence from the second recurrence onwards (*p* = 0.01).

Oophoropexy should be discussed after the second relapse of ovarian torsion. AMH level and volume of the ovaries could be reliable criteria for decision.

**O13. Unveiling the Enigma: Exploring Cryptic Pregnancy and Its Psychological Implications**

**N. Papari ^1^, D. Penekeli ^1^, C. Skentou ^2^, F. Gkrozou ^2^, A. Zikopoulos ^3^, A. Vatopoulou ^2^**

^1^  Faculty of Medicine, School of Health Sciences, University of Ioannina, Ioannina, Greece
^2^  Department of Obstetrics and Gynaecology, Medical School, University of Ioannina, Ioannina, Greece
^3^  Royal Devon and Exeter Hospital UK, Department of Obstetrics and Gynaecology, Exeter, UK

**Introduction and Aims of the Study:** Cryptic pregnancy is a phenomenon that challenges conventional understandings of reproductive processes and poses intriguing questions about the human body’s ability to conceal the presence of a developing fetus. We aim to delve into the intricacies of the phenomenon, examining its prevalence, physiological mechanisms, and the psychological impact on individuals experiencing it, and explore the profound implications it carries for both the individuals involved and the broader healthcare community.

**Methods:** Methods employed involve a comprehensive review of existing literature, clinical case studies, and an analysis of diagnostic tools and protocols utilized in obstetric practice. We explore the potential reasons behind the lack of detectability of pregnancy symptoms, addressing furthermore the stigma and misconceptions surrounding the phenomenon.

**Results:** Results shed light on the challenges in accurate identification and the subsequent implications for maternal and fetal well-being. It is approximated that 1 in 475 pregnancies go unnoticed until the 20-week milestone, and 1 in 2455 pregnancies persist undetected until labor. The likelihood of denied pregnancy is higher among very young or relatively older reproductive age individuals, with women of non-native cultural heritage being less frequently affected. Among the cited effects of the phenomenon, most significant are the psychological consequences for the mother, the possibility of unassisted childbirth and preterm delivery as well as neonaticide. Therefore, healthcare professionals must evaluate the maternal capacity to consent to obstetric interventions, while carefully assessing the mother’s best interests, if she lacks the capacity to do so.

**Conclusions:** The conclusions drawn from this exploration underscore the necessity for increased awareness, early diagnosis, and a holistic approach to managing cryptic pregnancies. We aim to contribute to a broader conversation about reproductive health, challenging preconceived notions and fostering a more informed approach to the experiences of those navigating the intricacies of cryptic pregnancy.

**O14. HPV Vaccination Status Among a Socioeconomically Deprived Population in Greece: An Extended Analysis**

**E. Tsarna ^1,2^, M. Marasioni ^1^, P. Grapsas ^1^, D. Balafoutas ^2^, E. Paschalidou ^2^, P. Christopoulos ^2^**

^1^  Department of OBGYN, General Hospital of Nikaia “Agios Panteleimon”, Athens, Greece
^2^  Division of Pediatric and Adolescent Gynecology, 2nd Departement of OBGYN, Medical School, National and Kapodistrian University of Athens, Athens, Greece

**Introduction and Aims of the Study:** The WHO has set the goal of 90% HPV vaccination coverage among girls up to 15 years old by 2030. In Greece, HPV vaccination was fully funded from the National Health System for women born between 1982 and 2008. The aim of this study was to estimate the extent of vaccination in a socioeconomically deprived population in Greece and to describe the characteristics of unvaccinated study participants.

**Methods:** This study was conducted among 1013 women born between 1982 and 2008, who provided a structured interview at the outpatient OB/GYN office between 2017 and 2023. Participants’ characteristics were evaluated between vaccinated and unvaccinated women with the Chi square test for categorical variables and the non-parametric Kruskal-Wallis Rank Sum Test for continuous variables.

**Results and Discussion:** An alarmingly low proportion of study participants (222 out of 1013, 21.2%) were vaccinated against HPV. Nonetheless, among 101 women below 20 years old, 50.5% were vaccinated. Unvaccinated study participants were born earlier, were less likely to be insured, were less likely to be single, had a lower educational level despite being older at time of interview, were more likely to be unemployed and not insured, were more likely to smoke, were more likely to have children at the time of the interview, and were more likely to have had at least one pap smear before the time of interview. With regard to contraception use, unvaccinated women were less likely to use condoms, but had an intrauterine contraceptive device and engaged in intermittent sexual intercourse for contraception more often. No other examined variable differed significantly between the two groups.

**Conclusions:** Among a socioeconomically deprived population in Greece, vaccination coverage was low, but has improved in younger participants.

**O15. Polycystic Ovary Sindrom in Adolescents: Challenges and Possible Solutions**

**E. Asanidze, J. Kristesashvili**

Teaching University Geomedi, Tbilisi, Georgia

Clarifying the diagnostic criteria of polycystic ovary syndrome (PCOS) and determining the benefits of different treatment methods is crucial for preventing long term reproductive, cardio-metabolic, and emotional consequences for adolescents.

**Aims of the Study:** Determine the possibility of using AMH for the diagnosis of PCOS in adolescents and evaluate the efficacy of different treatment methods.

**Methods**: A prospective cross-sectional study was carried out in 213 adolescents (13–19 years), 153 patients with PCOS (study group) and 60 healthy adolescents (controls). The study group was divided into: Group I-adolescents without insulin resistance (*n* = 60) and Group II-adolescents with insulin resistance (*n* = 93). Group I was treated with oral contraceptives (OCs), while Group II was divided into treatment subgroups of 31 patients each: Subgroup A received OCs; Subgroup B-myo-inositol; subgroup C-OCs + myo-inositol. Data were analyzed at baseline and 6 months of treatment.

**Results**: Average AMH level, total testosterone (TT), free testosterone (FT), mFG (Ferriman–Gallwey modified scale) and ovarian volume (Ov/v) were significantly higher in PCOS patients than in controls, *p* < 0.05. There was no significant difference in average antral follicle count (AFC) between PCOS patients and controls. ROC curve analyses demonstrated that the optimal value of AMH for predicting PCOS in adolescents was 6.52 ng/mL, with 69.5% sensitivity and 79.5% specificity. After treatment, in Group I and Group II Subgroup A: AMH, FT, TT, Ov/v, AFC, and mFG significantly decreased, homeostatic model assessment-insulin resistance (HOMA-IR), body mass index (BMI) did not change significantly. In Subgroup B only HOMA-IR and BMI decreased significantly; in Subgroup C all the parameters decreased significantly. The correlation between AMH and hormonal, morphological characteristics of ovaries were established.

**Conclusions:** AMH could possibly be a valuable marker for the diagnosis of PCOS in adolescents, and for the assessment of treatment efficacy as well.

**O16. The Differences Between Adults and Adolescents Using a Mobile Health Application for Menstrual Complaints: A Usability and Qualitative Study**

**H. Özcan^.1^, N. Burger ^1^, M. Derksen ^2^, L. Peute ^2^, J. Huirne ^1^, R. De Leeuw ^1^**

^1^  Department of Obstetrics and Gynaecology, Amsterdam Reproduction and Development Research Institute, Amsterdam UMC, AMC and VUmc, Amsterdam, The Netherlands
^2^  Department of Medical Informatics, eHealth Living & Learning Lab Amsterdam, Amsterdam UMC, University of Amsterdam, Amsterdam, The Netherlands

**Introduction:** A “Menstruatie Educatie Kalender” application (Menstruation Education Calendar, (MEK-APP)) was developed for adults to evaluate menstrual complaints. The future aim of this app is to use it as a self-diagnostic instrument for menstrual abnormalities for both adults and adolescents. Early identification of the potential of an application for future use by both user groups would increase implementation success and adoption of the application.

**Aim:** To compare differences in experienced usability by adults versus adolescents and to identify factors influencing future use for both age groups in one mHealth application (in this study the MEK-APP).

**Methods:** This study consisted of three phases: (1) usability testing of the MEK-APP for iOS and Android by think-aloud method, (2) two-month daily use and (3) in-depth individual interview. During the think-aloud sessions, twelve tasks were performed in the MEK-APP while they were thinking aloud. Usability problems were rated for their severity with Nielsen’ Severity Scale. Both the think-aloud sessions and in-depth interviews were verbatim transcribed and thematically analyzed to determine the factors influencing future use for both groups. In addition, the System Usability Scale (SUS) and Intrinsic Motivation Inventory (IMI) questionnaires were filled out during the interviews.

**Results:** Seven adults (>18 years) and seven adolescents (14–18 years) evaluated the MEK-APP. There were 14 usability issues and 16 bugs in both groups. There were no differences between adults and adolescents. In the thematic qualitative analysis, the following future use factors were identified: use-expectation, motivation, privacy, understandability, and user-experience. The use-expectation, motivation and privacy differed between both groups but did not influence usage. No differences were observed in SUS and IMI scores between both groups.

**Conclusions:** There are five factors influencing the future use of a menstrual-related mHealth application for both adults and adolescents. It is possible to serve different age groups with a single application.

**O17. Can a Seventeen-Year-Old Have Breast Plastic Surgery—A Case Study and Bibliography Review**

**P. Kalavas ^1^, K. Katifori ^1^, A. Vatopoulou ^2^, F. Gkrozou ^2^, C. Skentou ^2^, M. Paschopoulos ^2^**

^1^  University of Ioannina, School of Medicine, Ioannina, Greece
^2^  University General Hospital of Ioannina, Ioannina, Greece

**Introduction:** Τhere has been an increase in the number of Adolescent Girls and Young Females who seek to undergo Breast Plastic Surgery (BPS) and more specifically breast reduction or breast augmentation procedures. This increase has raised the concern of the medical community to find the causes that lead young females to surgery as well as the results/consequences in their later life, particularly in younger patients who undergo surgery during puberty.

**Aim of Study:** This study aims to present the reasons for which adolescent girls and younger females undergo BPS, as well as an attempt to present and understand the impact of these surgeries on a psychological and physical level both before and after surgery.

**Methods:** During the last 5 years, numerous studies have been contacted regarding BPS in adolescence, leading to various conclusions concerning this matter. By reviewing the literature, motivated by 2 cases of our own, we present our experience and conclusions regarding the current situation and what has yet to be done.

**Discussion:** In the decision making for BPS, physicians should consider various matters that strongly concern this age (e.g., psychosocial mental status, etc.), as well as developmental vulnerabilities in physical, psychological, and cognitive maturity. Regarding the outcome of the surgery, patients must have knowledge that due to their young age, changes in the size of breast from further development could require a second intervention. Patients should be counseled about possible scaring in the incision area. At the same time, potential future, financial consequences arising from medical expenses (e.g., cases of extreme gigantomastia, significant asymmetry) should also be considered, thus raising the question whether these procedures should be financially covered by the state.

**Conclusions:** The decision itself, whether a BPS should be performed in adolescent girls is challenging. All the details about the surgery as well as the possible side effects and precautions that the patient must take after the operation should be fully understood by the patients. Therefore, this necessitates the need for a multidisciplinary approach with the collaboration of physicians of various specialties.

**O18. Case Study of a Three Year Old Patient with Anogenital Condylomata Accuminata and Plan of Care**

**S. Roidi ^1^, A. Soldatou ^2^, V. Vasilopoulou ^2^, N. Poniros ^2^, L. Michala ^1^**

^1^  PAG Department Alexandra General Hospital, Athens, Greece
^2^  Agia Sofia Paediatric Hospital, Athens, Greece

**Introduction and Aim of the Study:** Condylomata acuminata (also known as anogenital warts) are manifestations of human papillomavirus (HPV) infection. It is known as a sexually transmitted disease. Condylomata accuminata can be diagnosed in children too. Because the concern of sexual abuse that comes with the diagnosis, the plan of care for these patients are a major challenge in practice. The aim of our study is review how to manage these cases using our patient as an example.

**Methods:** A three year old girl was reviewed in our outpatient department and diagnosed with perianal warts. A general pediatrician, a pediatric dermatologist and pediatric plastic surgeon were also involved and a referral to social services was made. It was her 7th recurrence when she was examined for the first time in our clinic. She had undergone two sessions of cryotherapy and four sessions of laser treatment. Because of her previous treatment history it was decided to continue with topical treatment with imiquimod. We are waiting for the next appointment to see her progress. Meanwhile our social services arranged interviews with her parents and school settings.

**Discussion:** Perinatal warts in children can be transmitted by autoinoculation, heteroinoculation (an infected caregiver that transmits it to the child), and vertical transmission in birth or sexual abuse. It is generally reported that under four years of age the possibility of sexual abuse is highly unlikely however cannot be excluded. There is no guideline for treatment other that the known methods used in adults.

**Conclusions:** The management of condylomata accuminata in pediatric patients is a major challenge for every physician. It requires a medical team of different specialties for the best result and social services to investigate the possibility of sexual abuse. The social impact and strain put on the families because of this diagnoses is significant and management must be case specific.

**O19. Prepubertal Vaginal Bleeding: A Retrospective Analysis of Prospectively Collected Data from a Tertiary Pediatric Gynecology Center in Greece**

**E. Iordanidou, A. Pana, S. Tsiapakidou, E. Klonos, T. Theodoridis, G. Grimbizis**

1st Department of Obstetrics & Gynecology, Aristotle University of Thessaloniki, Thessaloniki, Greece

**Introduction:** Vaginal bleeding in prepubertal girls often elicits concerns from parents and health providers alike. The cause of bleeding can be as simple as vulvovaginitis or as devastating as sexual abuse or malignancy. It is important for all health providers to familiarize themselves with the differential diagnosis of prepubertal vaginal bleeding in order to utilize the appropriate medical examinations and reach a prompt diagnosis.

**Aim:** The aim of this study was to describe the clinical features of children with prepubertal bleeding and the appropriate medical investigations to reach a prompt diagnosis.

**Methods:** A retrospective review of prospectively collected data was performed. All children under the age of 10, who presented with vaginal bleeding, between January 2012 and December 2023, were included in the analysis.

**Results:** A total of 8 cases were retrieved with a mean age of 5.7 years (range 2–8 years). Detailed individual and family history was taken from all patients and complete clinical examination was performed. Vaginal swabs were taken, bone age was assessed and a pelvic ultrasound of internal genitalia was performed. In 2 cases pelvic and brain MRI were carried out to assist differential diagnosis. Hormone testing and especially LH-RH (Luteinizing Hormone Releasing Hormone) stimulation test were prescribed when necessary. Moreover, n 3 cases (37.5%) vaginoscopy was deemed necessary. Conclusively, 3 cases (37.5%) were diagnosed with precocious puberty, 1 case (12.5%) was diagnosed with genital hemangioma, 2 cases (25%) were diagnosed with vaginal foreign body, and in the 2 remaining cases (25%) no diagnosis was reached and thus were assigned to a monitoring schedule.

**Conclusions:** Vaginal bleeding in prepubertal girls is a complex differential diagnosis problem that needs a holistic approach to investigate all possible causes. The cooperation between specialties is paramount to ensure the optimal handling of these incidents.

**O20. Vaginal Foreign Bodies in the Female Pediatric Age Group—Our Experience**

**A. Sima, D. Ivanova-Panova, D. Dabeski, S. Simeonova-Krstevska, V. Livrinova**

UGAK Skopje, Skopje, North Macedonia

**Introduction:** A common occurrence in female pediatric care is self-insertion of a vaginal foreign body (VFB). The reason is most often unintentional, when exploring the genitals with various objects, children’s play, maintaining hygiene, etc. Most often, this condition remains unrecognized, the main symptoms are vaginal discharge, bleeding, pain, foul-smelling discharge.

**Materials and Methods:** The population of girls up to 14 years old was retrospectively analyzed in the last 5 years. They reported to the University Clinic of Ob/Gyn in Skopje, North Macedonia due to suspected VFBs. They were examined in the emergency room, then continued evaluation, hospitalization, and treatment in the Department of Pediatric and Adolescent Gynecology. The gold standard for diagnosing and treatment of this condition is vaginoscopy, which was performed under anesthesia in every case. The removal of the foreign bodies resulted in full recovery for these patients.

**Results:** The incidence of VFBs was around 2.5%. The most common symptoms were vaginal discharge, bleeding, pain an unpleasant odor. Different types of foreign bodies were found—plants, paper, stones, decorative objects—pearls, cones. The patients underwent a gynecological examination, an inspection of the introitus vaginae area, followed by a vaginoscopy and the removal of the foreign body, as well as rinsing and disinfection of the vagina. Antibiotic prophylaxis was given for 5–7 days. There were no complications noted after the intervention.

**Conclusions:** VFBs should be considered in everyday pediatric practice. A multidisciplinary approach is needed in the diagnosis and treatment of this condition. A team of pediatricians, pediatric, gynecologists, and pediatric psychologists should provide safe and effective treatment, prevent complications, and prevent recurrence of the condition.

**O21. Vaginoresectoscopy in the Treatment of Ohvira in Adolescent Girls of the Russian Federation**

**Z. Batyrova, E. Uvarova, V. Chuprynin, Z. Kumykova, D. Kruglyak, F. Mamedova, E. Filippova, K. Kostukov, A. Asaturova, V. Karabatch, G. Sukhikh**

FSBI National Medical Research Center for Obstetrics, Gynecology and Perinatology Named after Academician V.I. Kulakov, Moscow, Russia

**Introduction:** OHVIRA syndrome is an obstruction of the hemivagina in the presence of a kidney anomaly on the ipsilateral side. The therapeutic approach in the management of such patients is to carry out surgical treatment in order to minimize complications and improve the quality of life. The classic method of treatment is to perform reconstructive plastic surgery in the volume of instrumental defloration and vaginal correction. This approach is characterized by long-term operating room occupancy and the risk of intraoperative complications. Previously described resectoscopic dissection of the asymmetric septum pointing out the high efficiency and low percentage of complications.

**Aim of the Study:** To evaluate the effectiveness and safety of vaginoresectoscopy in the treatment of OHVIRA.

**Methods:** 130 patients with OHVIRA were treated at the Pediatric and Adolescent Gynecology department from 2021 to 2023 y. According to the classical method n = 46, using vaginoresectoscopy according to the improved method n = 72. The average duration of the operation, blood loss, complaints in the early postoperative period, early and late complications were evaluated.

**Results:** The average duration of surgery in the main group (vaginoresectoscopy method) was 38.4 min versus 98.8 min in the comparison group; blood loss in the main group averaged 7.8 mL versus 55.2 mL in the comparison group; it should be noted that in most cases the girls noted the absence of any complaints affecting the general well-being already on the 1st day after the intervention, determining by VAS—the absence of pain. During the follow-up period from 2021 to the present, in the main group, stricture of the formed vagina was detected in 2.7% versus 4.3% in the comparison group.

**Conclusions:** Vaginoresectoscopy is an effective and safe method of surgical treatment for obstructive genital abnormalities.

**O22. Conservative Treatment for Vaginal Agenesis: When Surgery Is Not the ‘Primadonna’. A Multidisciplinary Experience**

**M. Lucchetti ^1^, A. Tassi ^2^, L. Spagnol ^1^, C. Carducci ^3^, C. Bizzarri ^4^, C. Ribaldone ^5^, M. Silveri ^1^**

^1^  Andrologic and Gynecologic Pediatric Surgical Unit, Bambino Gesù Children’s Hospital, IRCCS, Rome, Italy
^2^  Obstetrics and Gynecology Unit, Morgagni-Pierantoni Hospital, Forlì, Italy
^3^  Psycology Unit, Bambino Gesù Children’s Hospital, IRCCS, Rome, Italy
^4^  Endocrinology Unit, Bambino Gesù Children’s Hospital, IRCCS, Rome, Italy
^5^  General and Thoracic Pediatric Surgery Unit, Bambino Gesù Children’s Hospital, IRCCS, Rome, Italy

**Introduction and Aims:** Vaginal agenesis can be isolated or associated with various and different conditions, such as Rokitansky syndrome (MRKH syndrome), complete androgen insensitivity (CAIS) or anorectal malformations (ARMs). Various conservative and surgical treatment options are available for this condition. We report the results of vaginal dilation therapy delivered by a multidisciplinary team as the first-line treatment.

**Materials and Methods:** A retrospective review of electronic medical records was performed on patients aged 18 years or younger with vaginal agenesis in a tertiary pediatric hospital. Seventy-six patients were enrolled, 46 of whom with a diagnosis of MRKH, 20 of CAIS, and 10 with other associated malformations. Reference values for vaginal length were determined from previous literature.

**Results:** At diagnosis, patients with CAIS were younger (mean age 9.7 years) than those with MRKH (mean age 15.1 years). The diagnosis was communicated to the patient after a variable period of multidisciplinary work, always in the presence of the psychologist, endocrinologist and gynecologist. All treatment options were illustrated soon after diagnosis. Conservative treatment was started slightly earlier in MRKH patients (17.25 years vs. 18.5 years) and patients needed shorter time to be considered ‘highly motivated’. Nine of the 46 patients with MRKH had undergone previous vaginal reconstructive surgery, 33 patients had conservative therapy, and 4 patients were too young to start therapy. Two patients were affected by anorectal malformation. CAIS patients started conservative time later (probably as a conquence of a diagnosis requiring more time to be elaborated) but their results were faster. Further results will be illustrated in detail.

**Conclusions:** In the presence of vaginal atresia from any cause, isolated or associated with other malformative conditions, vaginal dilations remains the first choice therapy, but it has to be offered and managed by a motivated and competent multidisciplinary team to a motivated and competent patient.

**O24. The Risk of Gonadal Tumors in Patients with Disorders of Sex Development—Our Experience**

**V. Zovnir, T. Ivanova, I. Bachynska, I. Gavrilova, N. Pogadayeva**

National Children’s Specialized Hospital “Ohmatdyt”, Kyiv, Ukraine

**Introduction:** The presence of Y chromosome material in patients with disorders of sex development (DSD) associates with high risk of malignancy. Prophylactic gonadectomy recommended in females with bilateral intraabdominal location of gonads and Y chromosome.

**Aim:** To present our experience with prophylactic gonadectomy in those patients and evaluate their risk of gonadal tumors.

**Methods:** We reviewed the charts of 59 female patients with bilateral gonadectomy (laparoscopically in 58 patients, laparotomy in 1 patients) between 2005 and 2024 at NCSH “OHMATDYT”.

**Results:** 9 patients had chromosomal DSD (mosaic karyotype with Y chromosome) and intraabdominal location of gonads. 50 patients had DSD 46 XY with Swyer syndrome and CAIS with intraabdominal location of gonads. All patients had female phenotype. All were investigated by specialists of our multidisciplinary team with geneticist, endocrinologist, gynecologist, urologist, psychologist. All treatment tactics were approved by patients and their parents with the team’s counsel. Age at surgery ranged from 12 to 18 (mean 13.7) and follow-up—0.5 to 7 (mean 2.3) years. Pathologic examination revealed gonadal tumors in 13 of 59 patients (22%), all with 46XY karyotype, Swyer syndrome. Gonadoblastoma was detected in 3 gonads; association of dysgerminoma with gonadoblastoma was detected in 1 gonad; association of seminoma with gonadoblastoma—in 2, seminoma—in 1 gonad, dysgerminoma—in 2 gonads. Age ranged from 14 to 16 (mean 15.6) years old. MRI showed no metastasis, and postoperative course was uneventful in all patients (All patients consulted by oncologists).

**Conclusions:** In our series of DSD patients, risk of gonadal tumor was rather high. Considering high potential of gonadoblastoma for malignant transformation, careful investigation of patients and performance prophylactic gonadectomy are needed.

**O25. Use of a Vaginal Stent to Prevent Stenosis Following Vaginal Brachytherapy, How Long?**

**O. Dural ^1^, I. Evruke ^1^, S. Kucuck ^2^, U. Demirsoy ^3^, R. Kebudi ^4^, U. Yildirim ^4^, H. Ulusoy ^1^, H. Saygili ^1^, S. Akhan ^1^**

^1^  Department of Obstetrics and Gynecology, Istanbul University, Faculty of Medicine, Istanbul, Turkey
^2^  Institute of Oncology, Radiation Oncology, Istanbul University, Istanbul, Turkey
^3^  Pediatric Oncology, Kocaeli University, İzmit, Turkey
^4^  Institute of Oncology, Division of Pediatric Hematology-Oncology, Istanbul University, Istanbul, Turkey

**Introduction:** The long-term sequelae of vaginal brachytherapy (VBT), which can be used as an effective local treatment option for vaginal embryonal rhabdomyosarcoma (ERMS), is the risk of radiation-induced fibrosis and stenosis assumed to persist for up to 6 months after completion of therapy. We aim to report the long-term use a foley catheter as a vaginal stent in a girl underwent VBT.

**Case:** A two-year-old girl presented with a mass protruding through the introitus diagnosed with vaginal ERMS and treated with chemotherapy (CHT) alone due to complete response. Six months after the last CHT dose, because of the development of local recurrence, second-line CHT was initiated and the patient was referred to our institution for a local treatment plan. Following three cycles of CHT, intracavitary VBT was administered at different time periods with a total dose of 36 Gy. Six weeks after completion of VBT, vaginoscopy was performed with no evidence of residual disease and a nonlatex 22 French foley catheter placed in the vagina (Figure 2). The patient continued to receive CHT for about six month. With follow-up vaginoscopy performed every 3 months, the stent was replaced in the same fashion and remained in place for a total of 6 months. Since vaginal stenosis was detected 3 months after the stent was removed (Figure 3), the stent was placed again and remained in place for another 6 months. The patient did not develop any stent-related complaints. After 18 months of follow-up, control vaginoscopy revealed normal vaginal mucosa with no lesions or erosions.

**Conclusions:** Vaginal dilation, used as the primary method to prevent vaginal stenosis due to VBT in adults, is a traumatic method for young children. Our report shows that long-term use of vaginal stents up to 1 year may prevent the development of BT-induced vaginal stenosis.

**O26. Hematosalpinx in an Adolescent Girl with Heavy Menstrual Bleeding: A Case Report**

**L. Vogiatzi Vokotopoulou ^1^, M. Panagiotopoulos ^1^, M. Tsiriva ^1^, S. Kasioni ^2^, V. Triantafyllidi ^1^, E. Domali ^1^, G. Daskalakis ^1^, L. Michala ^1^**

^1^  1st Department of Obstetrics and Gynecology, ‘Alexandra’ General Hospital, National and Kapodistrian University of Athens, Athens, Greece
^2^  Department of Obstetrics and Gynaecology, Elena Venizelou Maternity Hospital, Athens, Greece

**Introduction:** Heavy menstrual bleeding (HMB) is a common complain among adolescent girls. Anovulatory cycles is the leading cause of HMB, in this age group, due to immaturity of the hypothalamic-pituitary-ovarian axis. For hemodynamically stable girls the work up includes assessment for anaemia, endocrine and bleeding disorders. Ultrasound is not always deemed necessary.

**Case Presentation:** We present the case of an 11 year old girl that was hospitalised 3 times in the course of 25 days due to menorrhagia and anaemia. During her initial evaluation she had heavy menstrual bleeding (changing 10–12 pads/day) for 14 days causing a hemoglobin drop to 7.5 g/dL. Her medical and surgical history was insignificant and she reported no other symptoms. She received one unit of packed red cells and the bleeding was managed with norethisterone orally. She was discharged 4 days later. During her re-hospitalisation there were no signs of significant bleeding and no further drop of the hemoglobin level. She had a routine transabdominal ultrasonography, which revealed an elongated mass (consistent with hematosalpinx). Due to the lack of other symptoms and inflammation markers in the normal range, a conservative approach was decided. The patient was given the combined oral contraceptive pill and a follow up was scheduled after one and four months. On her first follow up the mass had decreased significantly and on her second visit it was completely absorbed.

**Discussion:** Hematosalpinx in adolescents is rarely reported, and the aetiology is usually linked to obstructive uterovaginal anomalies. It is usually associated with lower abdominal pain. This case represents a curious and unusual finding of unilateral tubal enlargement, soon after menarche, without identification of menstrual obstruction. The most likely cause is retrograde menstruation caused by heavy menstrual bleeding. The mass resolved spontaneously within 4 months of initial presentation.

**O27. The Evaluation of Efficacy and Safety of Application of Ulipristal Acetate in the Treatment of Patients with Various Types of Uterine Fibroids**

**M. Kaviladze, S. Levakov, M. Dzhafarova**

I.M. Sechenov First Moscow State Medical University (Sechenov University), Moscow, Russia

**Objectives:** Nowadays there has been a steady and very intensive increase in the frequency of benign uterine fibroids. At reproductive age, uterine fibroids are detected in 40% of patients, affecting not only the quality of life of a woman, but also limiting her reproductive potential. Despite a fairly large clinical experience in the treatment of uterine fibroids, the effect of treatment is often incomplete, the disease progresses, which requires radical surgical intervention. In this regard, the search for the most accessible and highly effective methods of therapy is of great practical importance.

**Aims:** The aim of the study was to assess the efficiency and safety of treatment based on ulipristal acetate in patients of reproductive age diagnosed with simple or proliferating uterine fibroids.

**Material and Methods:** A prospective randomized study of efficiency of ulipristal acetate in 150 patients with simple (group I) and proliferating (group II) uterine fibroids was conducted in the Department of Gynecology in I.M. Sechenov First Moscow State Medical University. Histological and immunohistochemical study of uterine leiomyomas were performed by the Pathology Department.

**Results:** After 3 months of treatment, amenorrhea was observed in 70% of patients in group I and in 90% of patients in group II. After 3 months from the beginning of the therapy, reduction of the nodes diameter by 27% in group I and by 47% in group II was revealed. In 80% of patients in both groups, an average decrease of endometrium to 4–5 mm was found, in 10%—the thickness of the endometrium reached 8 mm and the last 10% of patients were found to increase the thickness of the endometrium to 12–14 mm. Ulipristal reduces the leiomyomas size not only due to the tumor cell apoptosis induction, and reduction of their proliferative and mitotic activity, but also due to angiogenesis and growth factors (VEGF, EGF, FGF-2, TGF-β1) inhibition in combination with the increased level of matrix metalloproteinases (MMP-2, -10, -12) production and their tissue inhibitors (TIMP-1, -2, -3) reduction.

**Conclusions:** Ulipristal causes the simultaneous negative impact on the parenchymal components, angiogenesis and extracellular matrix and leads to rapid, significant and sustained decrease in leiomyomas volume. The obtained data allow us to recommend the application of ulipristal acetate as preoperative preparation in the group of patients with simple and proliferating uterine fibroids.

**O28. Safety, Compliance and Pharmacokinetics of E4/Drsp in Post-Menarchal Female Adolescents**

**D. Apter ^1^, K. Haldre ^2^, G. Chatel ^3^, K. Gemzell-Danielsson ^4^**

^1^  VL-Medi Clinical Research Center, Helsinki, Finland
^2^  West Tallinn Central Hospital and East Tallinn Central Hospital, Tallinn, Estonia
^3^  Mithra Pharmaceuticals, Liège, Belgium
^4^  Department of Women’s and Children’s Health, Karolinska Institutet, Karolinska University Hospital, Stockholm, Sweden

**Introduction and Aims:** Estetrol 15 mg/drospirenone 3 mg (E4/DRSP) is an effective combined oral contraceptive (COC). E4, a native estrogen with selective action in tissues (NEST), combined with DRSP, exhibits predictable bleeding patterns and a low incidence of adverse events (AE). Due to the paucity of data in the youngest women using hormonal contraceptives for purposes beyond contraception and to comply with European Medicines Agency’s requirements, a phase 3, open-label, single-arm study was conducted across 23 European centers (NCT04792385). The primary objective is to assess the safety and tolerability of E4/DRSP in healthy post-menarchal females aged 12–17 years.

**Methods:** Adolescents aged 12–17 years old were enrolled in this study evaluating E4/DRSP in a 24-active/4-placebo regimen. Figure 4 summarize the study design. Treatment-emergent AEs (TEAEs) were recorded during the course of the study. Laboratory parameters and physical examinations were evaluated at baseline/cycle 6. Participants recorded daily drug intake, vaginal bleeding/spotting events and dysmenorrhea. The KIDSCREEN-27 and Menstrual Distress questionnaires measured psychological and social well-being at baseline/cycles 1/3/6. Plasma concentrations of E4, DRSP (cycle 1/6) and levels of sex hormone binding globulin and activated protein C resistance endogenous thrombin potential based were assessed (baseline/cycle 6).

**Results and/or Discussion:** Out of the initial 145 participants screened, 33 were excluded, and 23 withdrew prematurely, leaving 89 who completed the study. The last subject’s last visit was completed in November 2023. A favorable outcome concerning safety and tolerability is expected since no safety concerns affecting the benefit-risk ratio were observed.

**Conclusions:** This study will provide crucial insights into E4/DRSP’s safety, compliance, and bleeding profiles in post-menarchal adolescents, commonly prescribed COC for non-contraceptive purposes.

**O29. Safety and Tolerability of the Estetrol/Drospirenone Contraceptive Pill in Young Women up to 25 Years of Age**

**K. Haldre ^1^, F. Fruzzetti ^2^, K. Gemzell-Danielsson ^3^, T. Piltonen ^4^, A. Black ^5^, C. Bouchard ^6^, M. Chen ^7^, J. Foidart ^8^**

^1^  West Tallinn Central Hospital and East Tallinn Central Hospital, Tallinn, Estonia
^2^  Department of Obstetrics and Gynecology, Pisa University Hospital, Pisa, Italy
^3^  Department of Women’s and Children’s Health, Karolinska Institutet, Karolinska University Hospital, Stockholm, Sweden
^4^  Department of Obstetrics and Gynecology, Medical Research Center Oulu, Research Unit of Clinical Medicine, University of Oulu and Oulu University Hospital, Oulu, Finland
^5^  Department of Obstetrics and Gynecology, University of Ottawa, and The Ottawa Hospital Research Institute, Ottawa, ON, Canada
^6^  Clinique de Recherche en Santé de la Femme (RSF) Inc., Québec City, QC, Canada
^7^  Department of Obstetrics and Gynecology, University of California, Davis, Sacramento, CA, USA
^8^  Department of Gynaecology and Obstetrics, University of Liège, Liège, Belgium

**Introduction and Aims:** Estetrol 15 mg/drospirenone 3 mg (E4/DRSP) is an effective combined oral contraceptive. E4, a native and selective estrogen, combined with DRSP, ensures a regular bleeding pattern and low rates of adverse events (AE). This study assessed the safety and tolerability of E4/DRSP in young adults (16–25 years).

**Methods**: Two phase 3 trials (NCT02817828, NCT02817841) assessed E4/DRSP safety and tolerability over 13 cycles (24/4-day regimen). Participants (EU/RUS: 18–50 years, US/CAN: 16–50 years) with regular menstrual cycles and BMI of 18–35 kg/m^2^ were included. Pooled data analyzed frequency and severity of adverse events.

**Results:** The trials included 1736 subjects aged 16–25 years and 1491 aged 26–35 years. In the 16–25 years group, 49.4% (n = 857) reported AEs compared to 50.6% (n = 754) in the group of 26–35 years. The most commonly reported AE’s in 16–25-year-old versus 26–35-year-old were headache (5.8% vs. 5.5%), metrorrhagia, (4.6% vs. 5.0%), viral upper respiratory tract infections (3.9% vs. 3.2%), dysmenorrhea (3.8% vs. 2.3%, *p* < 0.05), urinary tract infection (3.7% vs. 1.5%, *p* < 0.0001), acne (3.6% vs. 3.8%), weight increased (3.1% vs. 2.5%), vaginal haemorrhage (3.0% vs. 3.4%), nausea (2.6% vs. 2.5%), abdominal pain (2.2% vs. 2.1%) and libido decreased (2.0% vs. 1.5%). In the group of 16–25 years, 176 participants (10.1%) discontinued treatment due to AEs compared to 140 (9.4%) in the group of 26–35 years. Serious AEs were reported by 22 (1.3%) of participants in the age of 16–25 years compared to 13 (0.9%) in 26–35 years-old group. Only 4 SAE were considered as possibly related to study medication (with two events/group).

**Conclusions:** The incidence of AEs was equivalent between the age groups except for dysmenorrhea and urinary tract infection in the 16–25 years group. These findings reinforce the favorable safety profile of E4/DRSP in young contraceptive users.

**O30. Menstrual Pain and Non-Communicable Diseases in Adolescent Girls—Results from Polka 18 Study**

**K. Rylewicz ^1^, M. Drejza ^2^**

^1^  Medical University of Warsaw, Warsaw, Poland
^2^  Cambridge University Hospitals, Cambridge, UK

**Introduction and Aim of the Study:** Menstrual pain is a prevalent issue among adolescent girls worldwide. It has a significant impact on their quality of life and has been linked to increased mental health problems, engagement in risky behaviours, and school absenteeism. There is limited research worldwide on the influence of non-communicable diseases (NCDs) on menstrual health. The aim of this study was to investigate potential associations between various NCDs and menstrual pain.

**Methods:** This research is part of the POLKA 18 study, a youth-led cross-sectional study aimed at assessing the knowledge, attitudes, and practices of Polish adolescents regarding their health and healthcare, with a special focus on sexual and reproductive health, conducted between April and December 2019. Study was funded by European Society of Contraception and Reproductive Health. Final year high school students were surveyed using self-reported paper questionnaires. The analysis was conducted using the R language in the RStudio environment. A *p*-value of less than 0.05 was considered statistically significant.

**Results:** A significant association (*p* < 0.05) was found between menstrual pain levels and heart and vascular diseases, hypertension, thyroid diseases, and gastrointestinal diseases. However, no association was observed between menstrual pain levels and asthma, diseases of the musculoskeletal system, acne, or psoriasis.

**Conclusions:** There is evidence to suggest that certain NCDs may be associated with higher levels of menstrual pain, indicating a potential impact of overall health burden on menstrual health. This highlights the importance of expanding outreach to menstruating adolescents with NCDs and providing gynaecological support and treatment for dysmenorrhea, if necessary, as they may be at increased risk. It also highlights the need for menstrual health awareness in specialist paediatric and adolescent healthcare settings. Further research is needed to establish causal mechanisms and better understand these relationships.

**O31. Virginity Certificates: Time to Wake Up!**

**D. Lentzaris, A. Vatopoulou, F. Gkrozou, C. Skentou, M. Paschopoulos**

University Hospital of Ioannina, Ioannina, Greece

**Introduction:** Virginity is a cultural and religious construction rather than a scientific one. Its definition and importance varies across the world. Some cultures value virginity while others oblige women to be virgin before marriage and display their vaginal bleeding after wedding in order to prove their virginity. Every year thousands of women are murdered worldwide in the name of honour, triggered by allegations of extramarital sexual relations and considered as the way of restoring the family’s honour. Consequently, young females being obliged or in order to protect themselves, request for virginity certificates or hymen restoration from healthcare provders. These practices not only trample the human rights but also lead to serious physical and psychological complications. Furthermore, Integrity or abnormalities of the hymen are not unequivocal signs of vaginal penetration, and so virginity cannot be certified by medical examination. Finally, while it is believed that existence of virginity testing reduces STDs, data shows the practice may cause the opposite!

**Aims of the Study:** Prove that virginity testing has no benefits, is unreliable and can cause complications, based on scientific data.

**Methods:** We searched the literature for data that scientifically prove virginity testing has to be abandoned regarding hymen anatomy variations, physical and psychological complications and consequences.

**Results and Discussion:** There is more than enough scientific data to support the virginity test elimination, as it has no benefits, cannot certify virginity and can lead to serious complications.

**Conclusions:** Although it is easy to scientifically prove virginity tests should be eliminated, it is hard to make it happen. In fact, the practice is still very common in numerous countries. Only appropriate information and education can beat the virginity taboo in a permanent way making certificates pointless.

**O32. Violence Against Women; Are Future Doctors Prepared? Creation and Evaluation of a Participatory Workshop for Greek Medical Students**

**M. Koutrouli ^1^, E. Avgerinopoulou ^1^, E. Kaliatsi ^1^, K. Dimakakou ^1^, L. Michala ^2^, G. Daskalakis ^2^**

^1^  National and Kapodistrian University of Athens, Athens, Greece
^2^  1st Department of Obstetrics & Gynaecology, NKUA, Alexandra Regional Hospital, Athens, Greece

**Introduction:** One in three women globally will face physical or sexual violence. Despite the alarming prevalence, the true extent remains unknown, confirming the iceberg phenomenon. Survivors of Intimate Partner Violence (IPV) are likely to have recurring contact with healthcare providers (HCPs) emphasizing the need for medical students to acquire essential knowledge and skills in identifying and addressing IPV.

**Purpose:** To: (1) develop a participatory educational workshop in IPV for medical students, (2) train and establish a peer-to-peer teaching group, and (3) assess the effectiveness of the workshop in gaining knowledge and improving self-confidence for participants.

**Methods:** We created a two-and-a-half-hour workshop with an introductory “pre-simulation briefing” part familiarizing students with crucial clinical characteristics of IPV and diagnostic tools. The second part was participatory incorporating role-playing and peer-to-peer teaching. Volunteer medical students were trained to simulate 4 real clinical scenarios, acting as patients/women. A 16-item knowledge and a 4-item self-confidence questionnaire were used before and after each workshop to assess its effectiveness.

**Results:** To date, two educational workshops have been conducted, with a total attendance of 21 participants. 11 students completed both pre and post questionnaires, showing statistically significant gains in knowledge and self-confidence (Mann Whitney test *p* < 0.0001). Self-selection bias testing confirmed the representativeness of the 11 students who completed both questionnaires.

**Conclusions:** We successfully established a peer-to-peer teaching group to conduct two workshops that improved the participants’ knowledge and self-confidence in identifying and managing violence against women. This feasible and novel active learning approach may help address inadequacies in medical curricula.

**O33. What Do Women Living with FGM Want Doctors to Know About Their Care?**

**M. Politis ^1^, A. Vatopoulou ^2^, C. Skentou ^2^, F. Grozou ^2^, M. Paschopoulos ^2^**

^1^  University of Ioannina, Ioannina, Greece
^2^  University General Hospital of Ioannina, Ioannina, Greece

**Introduction:** Female Genital Mutilation (FGM) persists with significant culturoreligious spread and varying prevalence in an estimated 130 countries. Due to migratory flow/refugee diaspora, health services operating in countries where FGM is illegal/not endemic, see an increasing number of women living with FGM. However, few health care practitioners (HCPs) are trained in treating the biopsychosocial complications associated with FGM, often compounded by difficulties of asylum/emigration, and may struggle to address the issue with patients and respectfully provide effective, transcultural care.

**Aim of Study:** This study aims to facilitate better communication/quality of care by elucidating points that women living with FGM have reported wanting HCPs to know regarding their interactions and treatment.

**Methods:** To this effect a review of existing literature was conducted, and the relative findings extracted. Furthermore, women living with FGM in refugee camps near Ioannina, Greece, were offered a questionnaire to evaluate their interactions with medical systems in their host countries, and offered an interview to voice their experiences and desires pertaining to their care. Interviews are ongoing and the final number of participants pending.

**Discussion:** Points that recur in testimonies include need for (1) active, respectful inquiry on behalf of doctors as to FGM status (keeping in mind that women may feel shy/consider complications a normal part of womanhood), (2) sufficient time and competent translation services; (3) communication and provision of treatment in a professional manner with sensitivity to trauma; (4) serious consideration of reported symptoms and values; (5) timely referral to specialized services; (6) patient empowerment. Safeguarding strategies have been flagged as vulnerable to paternalistic implementation, while supportive/informative outreach to affected communities remains strongly encouraged.

**Conclusions:** Mutual effort to improve doctor-patient communication and affirm patient autonomy results in improved treatment accessibility and quality of care. Resulting bolstered community awareness of complications engenders positive effects potentially extending beyond the scope of host countries.

**O34. Effect of Eating Behaviours and Duration of Symptoms on Bone Mineral Density (Bmd) in Patients with Atypical Anorexia Nervosa (AAN)**

**I. Evruke, O. Dural, I. Tas, H. Ulusoy, H. Saygili, C. Yasa, F. Gungor Ugurlucan, S. Akhan**

Istanbul University, Faculty of Medicine, Istanbul, Turkey

**Introduction:** AAN is an eating disorder with all the features of anorexia nervosa and significant weight loss in individuals who are not considered underweight. Despite the increased diagnosis of eating disorders, there is limited information available regarding the effect of AAN on bone health.

**Aim of the Study:** To evaluate BMD and its association with clinical factors that may affect bone health in adolescents with AAN.

**Methods:** The study enrolled 26 adolescents with AAN attending to gynecology clinic between 2020 and 2023. A retrospective evaluation was conducted to assess the factors that could impact adolescents’ lumbar spine dual-energy X-ray absorptiometry (DXA) data. Data on BMD, amenorrhea, duration of symptoms, body mass index (BMI) were evaluated (Table 3).

**Results:** The mean age at the onset of the symptoms was 16.2 ± 1.3 years, and body mass index (BMI) was 19.1 ± 2.1. 15 patients had a Z score lower than −1 (group 1), and 11 had equal to and greater than −1 (group 2). Although BMI and duration of the amenorrhea did not significantly differ in both groups, group 1 was found to experience a longer period of disrupted eating behaviours.

**Conclusions:** Our findings suggest that patients with disrupted eating behaviors, regardless of the duration of amenorrhea, are especially at higher risk for low BMD. We conclude that the duration of disruption of the eating behaviors has a negative correlation with BMD. Eating habits should be questioned in all patients presenting with amenorrhea, even with normal ranged BMI.

**O35. Adnexal Surgery in Adolescents. The Experience of a Tertiary University Hospital**

**A. Lazaridis ^1,2^, A. Mpagiasta ^1^, E. Tsarna ^1^, O. Triantafyllidou ^1^, N. Vlahos ^1^, P. Christopoulos ^1^**

^1^  2nd Division of OB/Gyn, Department of Paediatric & Adolescent Gynaecology, Aretaieion University Hospital, National and Kapodistrian University of Athens, Athens, Greece
^2^  St. Mary’s Hospital, Imperial College NHS Trust, London, UK

**Introduction:** Contrary to the common belief adolescent adnexal pathology is not uncommon albeit surgical management is mainly limited to persisting pathology or performed under emergency circumstances for pain or bleeding. Lack of expertise and experience often prevents young women from having laparoscopic procedures (1). Moreover, in certain occasions young women are treated with a radical approach including oophorectomy or salpingectomy, despite fertility and organ preservation being the main outcome of any surgical approach on that age group. Finally, in the modern era of enhanced recovery after surgery (ERAS) this population group should also benefit from the much valuable gained knowledge (2).

**Methods:** We retrospectively gathered information from all adolescent girls from the age of 14 years till 19 years old undergoing surgery for any adnexal pathology over a period of two years in our department of medical & surgical paediatric & adolescent gynaecology from January 2021 till December 2023.

We identified 15 cases of surgical management for ovarian or tubal pathology and reviewed all individual surgical notes and abidance to ERAS protocols.

**Results:** Out of 15 women, the vast majority (80%) was offered laparoscopic surgery with either a Hasson (58.3%) or Veress needle (41.6%) entry. There were no entry related complications identified, particularly that of vascular injury since the mean BMI was 20 kg/m^2^. Ten cases were performed for ovarian cystic lesions, with one of them having a histological diagnosis of mucinous borderline ovarian tumour. Additionally, three cases were performed for gonadal cryopreservation prior to receiving chemoradiotherapy for non-gynaecological malignancies. Blood loss was minimal with a median of 60 mls and there were no blood transfusions on this group of patients. Abiding to ERAS protocols, there was no utilisation of abdominal drains post-operatively and all laparoscopically treated patients had a trial without catheter within 6 h following their procedure and discharge was achieved in all laparoscopic cases within 24 h. Moreover, post-operative analgesia was achieved through regular Paracetamol and NSAIDs.

**Conclusions:** Through our caseload experience we have reviewed current surgical practices associated with adnexal pathology in adolescent girls and confirmed that laparoscopic approach is safe and feasible in centres with expertise and experience to manage them. Moreover, the ERAS protocol can and should be extrapolated to this population group since it offers significant benefits such as shorter hospitalisation and improved perception of the overall clinical experience.

**O36. Creation of Neovagina in Patients with Mrkh by Laparoscopically Modified Davydov & Longterm Sexual Outcomes: Eleven Years of Experience**

**G. Grimbizis, E. Iordanidou, A. Pana, G. Kioussis, S. Tsiapakidou, C. Anthoulakis, T. Mikos, T. Theodoridis**

1st Department of Obstetrics & Gynecology, Aristotle University, Thessaloniki, Greece

**Introduction:** Mayer Rokitansky Kuster Hauser (MRKH) syndrome has an incidence of 1 in 4000. Vaginal and uterine aplasia present with primary amenorrhea, sexual dysfunction and infertility, influencing the quality of life.

**Aim:** To present our experience in the creation of neovagina by laparoscopically modified Davydov procedure in patients with MRKH and to display the perioperative complications, post-operative anatomical findings and longterm sexual outcomes of these cases.

**Methods:** A retrospective analysis was conducted and 20 cases were identified with MRKH who underwent vaginoplasty by laparoscopically modified Davydov procedure between January 2012 and December 2023.

**Results**: A total of 20 patients with MRKH syndrome underwent laparoscopic modified Davydov procedure. All patients were operated by the same surgical team. All patients underwent detailed clinical examination and appropriate preoperative investigations. The mean age of patients at surgery was 18 years. Average vaginal length on arrival was 1.5 cm and width 2 cm. Average surgical time: 130 ± 20 min. Average intraoperative blood loss: 200 ± 100 mL. Postoperatively, the average length of the neovagina was 8 ± 1 cm and the width 4 cm ± 1 cm. One intraoperative complication was recorded: rectal serosa injury. Postoperative complications: three cases showed prolonged vaginal bleeding (>3 months) and two cases experienced urinary retention. The average postoperative follow-up time was 60 months (range 6–84 months). All cases were perioperatively monitored by a specialist in adolescent gynecology and a psychologist.

**Conclusions:** The Davydov method is a safe surgical technique for vaginal reconstruction in patients with MRKH. It should be recommended to all patients with vaginal aplasia, who either do not wish to use the self-dilatation method or do not achieve the desired result by using it.

**O37. Particular Case of Secondary Amenorrhea, a Case Report**

**M. Zanon ^1^, G. Impastato ^1^, P. Mertino ^1^, F. Pampaloni ^1^, M. Vangelisti ^2^, M. Fambrini ^1^**

^1^  Department of Experimental, Clinical and Biomedical Sciences, Division of Obstetrics and Gynecology, University of Florence, Careggi University Hospital, Florence, Italy
^2^  Department of Radiodiagnosis and Imaging, University of Florence, Careggi University Hospital, Florence, Italy

This paper shows the inconsistency between laboratory and radiology findings in a particular case of secondary amenorrhea. Despite some warning of a possible neoformation of the stromal line, it ultimately proved to be an anomalous form of polycystic ovary syndrome (PCOS).

In August 2023, a 15-year-old girl came to our observation at the Childhood and Adolescent Gynaecology Clinic. The girl presented a picture of secondary amenorrhea with menarche in May 2020 and no other subsequent menstrual period. The remote pathological history reported three operations for abdominal lipoblastomasis and treatment for precocious puberty from 2016 to 2019.

Against this background, we performed an ultrasound examination, which showed bilaterally enlarged ovaries occupied by numerous cystic formations as in polycystic ovaries. The scan detected a hyperechoic pseudonodular formation with clear margins of 45 × 30 mm in the right ovary. Therefore, we requested an in-depth MRI to examine the suspect formation, suspecting two possible different diagnosis of an enlarged ovary due to PCOS or a neoformation of the stromal line. The dosage of ovarian tumour markers highlights positivity only for inhibin B while the hormonal dosages are compatible with PCOS. In December, considering the radiological images and the positivity of inhibin B, we carried out a right ovarian resection and peritoneal staging surgery on suspicion of a neoformation of the stromal line. The celioscopy showed a regular right adnexus, from which originates a cystic capsulated pedunculate formation of about six centimeters. Histology exams revealed that this formation was composed by multiple ovarian follicular cysts and the peritoneal cytology was negative.

In conclusion, it emerged that the laboratory and radiological findings were discordant with the final diagnosis. Despite the warning of a neoformation of the stromal line, this clinical picture is likely to be an anomalous presentation of PCOS.


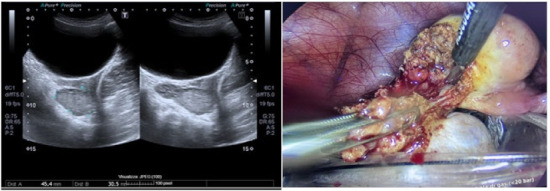

**O38. Polycistic Ovary Syndrome and Vitamin 25(OH)D Levels in Adolescents: Is There Really a Connection?**

**A. Papanikolaou ^1^, A. Vatopoulou ^1^, I. Georgiou ^1^, A. Serbis ^2^, K. Tsiaras ^3^, M. Paschopoulos ^1^**

^1^  Department of Obstetrics and Gynaecology, Faculty of Medicine, School of Health Sciences, University of Ioannina, Ioannina, Greece
^2^  Department of Pediatrics, Faculty of Medicine, School of Health Sciences, University of Ioannina, Ioannina, Greece
^3^  Faculty of Medicine, School of Health Sciences, University of Ioannina, Ioannina, Greece

**Introduction and Aims of the Study:** Emerging evidence suggests a potential interplay between PCOS and vitamin 25(OH)D deficiency in adolescents. The aim of this review is to elucidate if there is a statistically significant correlation.

**Methods:** We performed a clinical trial and case-control search in Cochrane Database searching for articles published from 2013 until 2023. We started our search by applying the key words: PCOS and Vitamin D and Adolescent. Only 9 out of the 28 studies were relevant. Nine studies were included (773 adolescents) investigating the deficiency of vitamin 25(OH)D on PCOS adolescents. One study is about a clinical trial and the rest studies, eight in total, are case controls.

**Results:** Although vitamin 25(OH)D levels of all the PCOS adolescents were lower than the control groups, significant correlation (*p* < 0.05) is found in only 3 out of 9 studies (337 adolescents-45%). Moreover, we observe that the lowest mean age observed in PCOS patients and in control group is 14.9 and 14.4 respectively and the highest is 19.4 and 20. Also, the minimum level of vitamin 25(OH)D in PCOS girls is 13.40 and the maximum is 24.88. Furthermore, the results indicate that the lowest level of vitamin 25(OH)D in the control group is 16 and the maximum is 58.5.

**Conclusions:** Although in all studies the vitamin 25(OH)D levels are lower in the PCOS subjects than in the control group, indicating that there may be a connection between adolescents with PCOS and low vitamin 25(OH)D levels, there is not enough data to prove a direct statistically significant correlation as in adults. Comprehending this correlation is crucial in clarifying the genesis of PCOS and refining preventing and therapeutic approaches, underscoring the significance of further and focused investigations.

**O39. Etiology and Diagnosis of Primary Amenorrhea: Updated Data from a Tertiary Level Center in Northern Greece**

**A. Pana, E. Iordanidou, S. Tsiapakidou, C. Anthoulakis, E. Bili, T. Theodoridis, G. Grimbizis**

1st Department of Obstetrics & Gynecology, Aristotle University of Thessaloniki, Thessaloniki, Greece

**Introduction:** Primary amenorrhea is a distressing problem for parents and patients alike. These patients are both misdiagnosed and mismanaged at numerous occasions.

**Aim:** The objective of this study was to collect and present data, with respect to etiology and diagnosis, from patients referred to a pediatric and adolescent gynecology tertiary level center in Northern Greece following the initial diagnosis of primary amenorrhea.

**Methods:** A retrospective analysis of prospectively collected data from all female patients with primary amenorrhea was performed between January 2016 and December 2023. All patients were assigned to one of the following groups and subgroup analysis was performed. Group A consisted of patients 13-year-old or older with no pubertal development. Group B consisted of patients 15-year-old or older with no menses. Group C consisted of patients with no menses, irrespective of age, three years after breast augmentation.

**Results:** A total of 40 patients were retrieved from the archives. Mean age at presentation was 16.4 years of age (14–23). In Group A, 8 (20%) patients were identified. 7 patients tested positive for progesterone challenge test (PCT), and 1 was diagnosed with Mayer-Rokitansky-Küster-Hauser (MRKH) Syndrome. In Group B, 15 (37.5%) patients were identified. 8 patients tested positive for PCT, 3 patients were diagnosed with primary ovarian insufficiency (1 diagnosed with Turner Syndrome), 2 with congenital malformations (U0C0V3 and U4aC4V4), and 2 with MRKH Syndrome. In Group C, 17 (42.5%) patients were identified. 4 patients tested positive for PCT, 1 was diagnosed with primary ovarian insufficiency (Turner Syndrome), 1 with congenital malformation (U0C4V4), 1 with complete androgen insensitivity syndrome (CAIS), and 10 with MRKH Syndrome.

**Conclusions:** Primary amenorrhea may result from a number of different conditions. A systematic evaluation including a detailed history, physical examination, and laboratory assessment of selected serum hormone levels can usually identify the underlying cause.

**O40. Metabolic Impact of Hormonal Replacement Therapy in Premature Ovarian Insufficiency**

**G. Impastato, M. Zanon, P. Mertino, F. Pampaloni, F. Petraglia, V. Bruni**

Division of Obstetrics and Gynaecology, Department of Experimental, Clinical and Biomedical Sciences, University of Florence, Careggi University Hospital, Florence, Italy

**Objective:** The purpose of the study is to highlight, the effects of Hormone Replacement Therapy (HRT) at metabolic level in patients affected by Premature Ovarian Insufficiency (POI), evaluating the lipid, glycaemic, and cardiovascular profile, bone health, thyroid function, and liver functionality.

**Methods:** We selected 149 patients with both primary and secondary Premature Ovarian Insufficiency (POI) from the Gynaecology of Childhood and Adolescence Clinic at Careggi University Hospital, Florence. All patients were under 35 years-old, and with a gynaecological age not exceeding ten years. We evaluated anthropometric data, risk factors, gynaecological age, characteristics of previous menstrual cycles, haematological, hormonal, and bone values, the presence of FMR1 mutations through FISH technique, family history, and the treatment received, analysing the type of oestrogen and progestin.

**Results:** HRT has shown beneficial effects on bone health with an improvement in bone mineral density values. The treatment predominantly included a combination of oestradiol and oral dydrogesterone. in patients with secondary amenorrhea, higher doses formulations (oestradiol 2 mg + dydrogesterone 10 mg) were mainly administrated to those with a gynaecological age of less than 5 years, while lower dosages (oestradiol 1 mg + dydrogesterone 10 mg) were given mainly to the population with a gynaecological age between 6 and 10 years.

**Conclusions:** The novelty of this study stems from the choice of the patients’ age range. As women under 35 are less likely to experience the cessation of ovarian function and its short and long-term effects, they are often overlooked in POI studies. The evidence shows important psychological implications as well as practical consequences for their possibility of reproduction. HRT represents, for these women, a guarantee of maintaining the proper functioning of the skeletal system and sexuality.

**O41. Is It Anorexia Nervosa or Is There Another Cause for Amenorrhea? Amenorrhea as the Presenting Symptom of Cushing Disease**

**S. Ben-Harush Negari, C. Avnon Ziv**

Shaare Zedek Medical Center, Jerusalem, Israel

**Introduction:** Anorexia nervosa (AN) is a life-threatening disorder with significant mortality and morbidity. Malnutrition in AN often presents with hypothalamic amenorrhea.

**Aim:** To discuss amenorrhea as the presenting symptom of Cushing disease (CD) in a patient with AN.

**Discussion:** We describe a 12-year-old female with rapid 12 kg weight gain and anxiety due to environmental changes in her life (from 52 kg to 64 kg). A year later, she rapidly lost 20 kg and was diagnosed with atypical restrictive AN. She was treated in a multidisciplinary adolescent clinic with psychotherapy, dietitian counseling and Sertraline. Primary amenorrhea was attributed to her malnutrition. At the age of 15, after successful treatment, her weight was stable at 53 kg. Despite resolution of anxiety and behavioral recovery she did not commence menses. Since her breast tanner staging did not fit the amenorrhea, a combined ACTH and LHRH test was done. LH rose from 1.4 IU/L to 26.7 and FSH from 4.1 IU/L to 15.2, cortisol rose from 615 to 1630 nmol/L. A full workup including free urinary cortisol, several different dexamethasone suppression tests and salivary midnight cortisol all showed high levels of cortisol and ACTH. MRI showed a 4 mm pituitary microadenoma. She underwent a transsphenoidal resection of the tumor successfully. Pathology demonstrated a sparsely granulated corticotroph adenoma. Two months after resection she commenced menstruating and has regular periods. Her weight and eating habits are stable.

**Conclusions:** CD clinically overlaps with AN and anxiety. CD in children can manifest as growth failure, irregular menses, and depression. CD is difficult to diagnose, especially in AN patient. It is important to consider other potential etiologies for amenorrhea rather than malnutrition alone, such as CD.

**O42. Reassesing Primary Ovarian Insufficiency Workup Guidelines: A Call for Change**

**N. Gruber ^1,2^, H. Raanani ^3^, H. Shani ^2,4^, M. Segev ^2,3,4^, O. Barel ^2,5^, R. Mizrahi Sapir ^1,2^, A. Hourvitz ^2,6^, O. Pinhas-Hamiel ^1,2^, A. Kedem ^2,6^**

^1^  Pediatric Endocrine and Diabetes Unit, Edmond and Lily Safra Children’s Hospital, Sheba Medical Center, Tel-Hashomer, Ramat Gan, Israel
^2^  Tel Aviv University, Tel Aviv, Israel
^3^  Fertility Preservation Center, Department of Obstetrics and Gynecology, Sheba Medical Center, Tel-Hashomer, Ramat Gan, Israel
^4^  Clinical Genetics, Obstetrics & Gynecology, The Danek Gertner Institute of Human Genetics, Sheba Medical Center, Tel-Hashomer, Ramat Gan, Israel
^5^  Genomics Unit, Sheba Cancer Research Center, Sheba Medical Center, Tel-Hashomer, Ramat Gan, Israel
^6^  IVF Unit, Shamir Medical Center (Assaf-Harofeh), Zerrifin, Israel

**Introduction and Aims:** The current guidelines for the genetic workup of primary ovarian insufficiency (POI) include performing karyotype and assessing the Fragile X carrier state (FXS). We aimed to investigate the genetic etiologies of POI and assess the need for updated guidelines for POI workup.

**Methods:** We conducted a prospective trial that included individuals with non-iatrogenic, normal karyotype POI, referred to two endo-gynecologic fertility clinics. Demographic, clinical, laboratory, and imaging data were collected, and blood samples were drawn to extract DNA for FXS analysis and whole exome sequencing (WES), followed by extensive bioinformatics analysis.

**Results:** A total of 30 individuals (13 adolescents) with a mean age of 24.1 ± 8.7 years, were recruited. The mean age of menarche was 13.1 ± 1.5 years, 21 experienced menstrual irregularities, whereas 9 had normal menses with infertility, and 12 had familial POI. We identified a genetic variation in 13 (43%) cases that might explain POI. Six were classified as likely pathogenic or pathogenic (2 FXS, 2 *FIGLA*, *SYCE1*, and deletion and duplication in chromosome 22). All of these variations were novel except FXS. In addition, seven cases were classified as a variant of unknown significance in genes previously reported in a few individuals with POI (*EXO1*, *FANCA*, *RREB1 & ATG9B*, *BMP8*, *POR*, *STAG3*) and in *GREB1*, which was described only in mice. Two other pathogenic variants were found in genes unrelated to POI (*PKD2*, *PROKR2*), but with further clinical implications. The results led to five fertility preservations among the individuals’ sisters and yielded precise counseling for both the patients and their families.

**Conclusions**: Identifying the genetic etiology of POI enables personalized medicine, improves care and fertility preservation for the index patient and her family, and contributes to the understanding of this phenomenon. We assert that WES should be integrated into the guidelines in the POI workup.

**O43. Endometriosis in Adolescents with Non-Contributive Clinical and Imaging Examinations**

**K. Kwaśniak ^1^, W. Zajączkowska ^2^, K. Kapczuk ^2^**

^1^  Division of Gynecology, Gynecology and Obstetrics Clinical Hospital of Poznań University of Medical Sciences, Poznań, Poland
^2^  Division of Gynecology, Poznań University of Medical Sciences, Poznań, Poland

**Introduction:** Adolescent endometriosis is distinct from adult endometriosis and in many cases definitive diagnosis can be made only with laparoscopy. The study aimed to evaluate adolescents who underwent diagnostic laparoscopy due to dysmenorrhea non-responsive to treatment with non-steroidal anti-inflammatory drugs (NSAIDs) and combined hormonal contraceptives (CHCs).

**Material and Methods:** The study group involved 31 adolescent girls with a suspicion of endometriosis who between January 2020 and September 2023 in Division of Gynecology, Gynecology and Obstetrics Clinical Hospital of Poznań University of Medical Sciences (tertiary level hospital), Poznań, Poland, underwent laparoscopy due to dysmenorrhea resistant to treatment with NSAIDs and CHCs (at least 4 cycles of therapy). In each patient laparoscopy was performed after at least 3 months without hormonal treatment. Adolescents with concomitant obstructive anomalies of the genital tract or endometriosis detected in imaging (pelvic US or MRI) were excluded from the study.

**Results:** The median age at surgery was 17.0 (range of 10.6 to 18.3) years. Endometriosis was diagnosed in 19/31 (61.3%) subjects and ruled out in 12/31 (39.7%) subjects: 16/19 (84.2%) adolescents had peritoneal endometriosis and 3/19 (15.8%) had ovarian endometriosis. In 17 patients endometriosis was visually confirmed. Two of 14 patients (14.3%) with negative laparoscopic visual inspection for endometriosis had positive histology. Stage I of endometriosis (according to the revised American Society for Reproductive Medicine (rASRM) classification) predominated (16/19 subjects (84.2%)). Acyclic bleeding, dyspareunia, and dyschezia were observed exclusively in the group with confirmed diagnosis of endometriosis. The incidence of mental health disorders in the group with and without endometriosis was similar (*p* > 0.05).

**Conclusions:** At least 60% of adolescents with non-contributive clinical and imaging examinations and dysmenorrhea non-responsive to treatment with NSAIDs and CHCs are affected with endometriosis.

**O44. Bone Growth and Final Adult Height in Girls with Central Precocious Puberty**

**A. Athanasiadis, A. Vatopoulou, F. Grozou, C. Skentou, M. Paschopoulos**

University of Ioannina, Ioannina, Greece

**Introduction:** Central precocious puberty refers to puberty occurring before the bibliographically referred age of 8 years old, with the premature activation of the hypothalamic-pituitary-gonadal axis, affecting nearly 1:10,000 children, mainly girls. CPP may develop due to certain conditions and pathologies, like CNS tumors, genetic predisposition, primary hypothyroidism and infections, however the most common cases are idiopathic (ICPP). Signs such as the appearance of secondary sexual characteristics, the start of menstruation and the rapid height growth should raise concerns for a possibility of CPP and further investigation must be done. GnRH analogues are used as a therapy to postpone puberty and positively affect the expected musculoskeletal development.

**Aim:** The aim of this review is to discuss the implications of CPP at the physical height growth and the bone maturation, investigate the long-term outcomes of the therapy with GnRH analogues and focus on information about final adult height prognostic models.

**Methods:** Numerous publications of worldwide recognized authors were reviewed and we selected the most relevant and new data to our subject (from the last 3 years).

**Results/Conclusions:** There is a significant correlation between CPP and diminished adult height due to the early maturation of the epiphyseal growth plate. GnRH analogues are the treatment of choice, because they are safe and efficient at restoring genetic growth potential and slowing down puberty. However, regarding multiple factors that affect final adult height, such as BMI, predicted adult height and diagnosis age, the dosage has to be adjusted individually and combination therapies should be taken under serious consideration. Based on the recent bibliography, there exist many adult height prognostic models some of them proved to be extremely precise.

**O45. Sexual Health and Reproductive Literacy (Shrl) Among Adolescent Girls Attending Menstrual Health Seminar in Tertiary Hospital**

**I. Ahmad Mizam ^1^, A. Nur Azurah ^1^, E. Sweet Yi ^1^, N. Nik Mohd Nor ^1^, L. Lim ^1^, J. Azhary ^2^, N. Abu Ishak^3^, A. Zainuddin ^1^**

^1^  Faculty of Medicine, Universiti Kebangsaan Malaysia, Kuala Lumpur, Malaysia
^2^  Hospital Pantai Bangsar, Kuala Lumpur, Malaysia
^3^  Kulliyyah of Medicine, International Islamic University Malaysia, Kuantan, Malaysia

Although sexuality education has been implemented in Malaysian educational system, the raising health issues among adolescent girls remain to be alarming. Furthermore, taboo still exist in regard to discussing sexual and reproductive health issues with these girls. The aim of this study was to determine the Sexual Health and Reproductive Literacy (SHRL) among adolescent girls aged 13 to 17 years old attending the Menstrual Health Seminar in a university hospital. A cross-sectional study was conducted using a self-administered validated questionnaire. Descriptive, One Way ANOVA and Pearson chi-squared were used to analyse the data.

A total of 341 female students participated. Malaysia consists of three main ethic groups which are Malays (60%), Chinese (30% and Indians (10%). However, a vast majority of respondents was Malays (99.1%) and 99.7% were Islamic religion. About 107 (31.4%) studied secondary 1, 73 (21.4%) secondary 2, 41 (12.0%) secondary 3 and the rest secondary 4 (35.2%). The awareness for menstrual cycle was high among the students (95.3%); however, only around half of the students were awared on HIV and AIDS (52.8%), condom (48.7%), wet dream (44.0%), birth control pill (51.6%), abortion service (40.5%) and contraceptive pill (49.3%). The mean overall scores were 3.84 (SD: 2.45). In general, the students demonstrated good knowledge level with scores of more than 80%. The mean reported knowledge score was 12.06 (SD: 1.36). The attitude of the students were quite positive with mean score of 4.26 (SD: 0.50). There were 38 (11.1%) students reported that they have watched pornography materials, while 52 (15.2%) have read pornography materials. Significant disparities arise in terms of awareness, knowledge, and attitude among students with different educational levels (*p* < 0.001). These findings suggest a need for targeted interventions tailored to educational backgrounds, emphasizing the importance of educational institutions in fostering sexual health awareness.

**O46. Chlamydial Infection in Puberty: Evaluating Our Knowledge**

**A. Marogianni, M. Tsiriva, V. Triantafyllidi, G. Daskalakis, L. Michala**

1st Department of Obstetrics & Gynaecology, Alexandra Hospital, University of Athens, Athens, Greece

**Introduction and Aims of the Study:** Almost 2/3 of total chlamydial infections are diagnosed in pubertal girls, making this group particularly vulnerable to sequelae of Pelvic Inflammatory Disease (PID). This is rightly reflected in the latest Centers for Disease Control and Prevention (CDC) Guidelines, which recommend annual screening for all asymptomatic sexually active pubertal girls. Similar protocols have been developed by the American College of Obstetricians and Gynecologists, and Pediatric Societies. Despite similar guidance from European societies, uptake for chlamydia testing is low in Greece, with few cases being recorded in the National Registry.

**Methods:** We developed a Knowledge, Attitude and Practices (KAP) questionnaire, with an aim to identify trends in chlamydia testing and treating intentions among obstetricians and gynecologists in a tertiary teaching hospital. The questionnaire was devised based on information acquired from National and International protocols on Chlamydial infection in puberty. It was then transcribed on Google Forms and distributed electronically among medical staff. The study received ethical approval from the Institution’s Scientific board, Number 41/2nd/30 January 2024.

**Results:** 55 out of 60 participants answered the questionnaire, of which 30 were trainees. 57% were not aware of the recommendation for annual chlamydial screening in sexually active pubertal girls. Furthermore, 30% felt that culture is the gold standard for chlamydial infection diagnosis. 93% answered that they would treat sexual partners. 65% were unsure whether parental consent is required for a gynecological examination on an adolescent. Only 49% answered correctly that first line treatment for chlamydial cervicitis is doxycycline.

**Conclusions:** We demonstrated that knowledge regarding chlamydia is deficient, with particular gaps in screening, treatment regiments. There was also confusion regarding the ability to examine and treat an adolescent without parental consent, indicative of an unclear legal framework on the matter. Following this, we plan to reinforce teaching around chlamydial infection in puberty and audit by readministering the questionnaire after implementation.

**O47. Do We Need Pediatric and Adolescent Gynecologists?**

**M. Florea, K. Matusiak, M. Walewska-Wolf**

Children’s Memorial Health Institute, Pediatric and Adolescent Gynecology Clinic, Warsaw, Poland

**Introduction and Aims of the Study:** First gynecological visit may have a significant impact on a girl’s life and health choices so how it should look like? We decided to conduct the study determining whether the girls’ carers know when to report for a preventive visit or in case of gynecological problems, whether they seek medical help and where, whether they know how a gynecological visit is performed and what problems they want to discuss during such a visit.

**Methods:** A questionnaire esigned to collecting information about the knowledge of girls’ carers, distributed in schools, gynecological clinics and by family doctors and pediatricians. The data were analysed using student’s *t*-test and chi square test.

**Results:** A total of 418 questionnaires were obtained. We received the following information, among others. 50% of respondents believe that a girl should go to a gynecologist regardless of age if she has gynecological problems, usually in such a situation they would go to a pediatric gynecologist (37%), gynecologist (29%) or pediatrician (18%). 35% believe that a girl should come to the visit only with her mother. Even though 97% declare that they talk to their daughter and try to prepare her for the examination, at the same time 32% do not know what a gynecological examination of a girl looks like and 7% believe that it looks the same as an examination of a woman who has had sexual intercourse.

**Conclusions:** Girls’ caregivers see gynecologists and pediatric gynecologists as an important part of the health team caring for their daughters. They look for help from them both in case of problems and for a preventive visit. However, it is worth putting more emphasis on educating caregivers regarding the course of the visit.

**O48. Endocrine Disrupting Chemicals and Association with the Diabetes Mellitus Type 1: A 5 Year Systematic Review**

**S. Tsokkou ^1,2^, G. Keskesiadou ^1,3^, T. Papamitsou ^1,4^, S. Karachrysafi ^1,5^**

^1^  Member of the Research Team “Histologistas”, Interinstitutional Postgraduate Program “Health and Environmental Factors”, Medical Department, Faculty of Health Sciences, Aristotle University of Thessaloniki, Thessaloniki, Greece
^2^  3rd year Undergraduate Student, Medical Department, Faculty of Health Sciences, Aristotle University of Thessaloniki, Thessaloniki, Greece
^3^  Resident of Paediatrics, Paediatric Clinic, General Hospital of Drama, Drama, Greece
^4^  Laboratory of Histology-Embryology, Medical Department, Faculty of Health Sciences, Aristotle University of Thessaloniki, Thessaloniki, Greece
^5^  Academic Fellow, Laboratory of Histology-Embryology, Medical Department, Faculty of Health Sciences, Aristotle University of Thessaloniki, Thessaloniki, Greece

**Introduction:** Type 1 Diabetes Mellitus (T1DM) is an inclining issue especially in the adolescence group of individuals. The diabetes atlas (7th edition), states that the global prevalence of diabetes is estimated at 415 million (8.8%) and predicted to increase up to 642 million in the following 25 years. T1DM is an autoimmune disease that results in the destruction of β-cells—insulin producing cells found in the pancreatic islets. The lack of insulin leads to hyperglycemia and impaired glucose utilization in the skeletal muscles. It has been suggested that the autoimmune process against β-cells seems to be also determined by environmental triggers, such as endocrine disrupting chemicals (EDCs). EDCs are a group of exogenous compounds with high heterogeneity, found naturally in living organisms or synthesized industrially. Environmental EDCs can affect the development and the fuction of the β-cells and immune system as a whole, promoting autoimmunity. The aim of this study is to examine the association of EDCs and development of T1DM among children and adolescence.

**Methodology:** A PRISMA flow diagram was performed to examine the association between ECDs and T1DM based on the following criteria. Papers had to be published at a 5-year duration, be in form of “open access” and the databases used where SCOPUS, ScienceDirect and PubMed. Exclusion criteria were animal studies, papers not relevant from the title or abstract examination and papers not relevant from their content.

**Results:** After the criteria were applied a total 1092 documents were found and examined from the 3 databases and after exclusion, the articles were narrowed down, and the most relevant studies were selected.

**Conclusions:** Based on the examination performed and the articles studied a high association between ECDs and the development of T1DM was found, especially among Persistent organic pollutants, Phthalates and Bisphenol A.

**O49. What Is Known About Coping with Stress and Expected Pain Before a Gynecological Visit?**

**J. Brodowska ^1^, A. Torres ^1^, P. Pawłowski ^2^**

^1^  Department of Pediatric and Adolescent Gynecology, Medical University of Lublin, Lublin, Poland, Lublin, Poland
^2^  Student Scientific Association at the Department of Psychology, Faculty of Medicine, Medical University of Lublin, Lublin, Poland

**Introduction and Aims of the Study:** A gynecological visit is a difficult experience for women. It may be associated with anxiety, embarrassment or shame. There are a limited number of publications on anxiety and expected pain, as well as methods to reduce them. This is important because the experience from previous consultations may have an impact on the frequency of subsequent check-ups. The main aim of study was to review the literature regarding the possibility of reducing anxiety and expected pain before a gynecological visit.

**Methods:** The search for articles included the databases PubMed and Web of Science, using the terms: stress; fear; anxiety; gynecological visit; gynecological examination; pelvic examination; vaginal examination. Firstly 1169 articles were retrieved and secondly two independent reviewers checked titles and abstracts to assess against the inclusion criteria. The risk of bias and the certainty of the evidence for each outcome were evaluated. On the end full articles were read and the final was done.

**Results:** The analysis allowed to identify two main topics: (1) emotions related to gynecological visit, (2) interventions used to reduce anxiety and expected pain. The selected interventions were grouped into four categories: education; gynecological environment; communication; multimedia techniques. Most of research was conducted on adults. Trials related to coping with stress in pediatric or adolescent group were relatively niche.

**Conclusions:** Checking whether stress-reducing interventions used in adults are effective among younger girls is recommended, because of different levels of mental and physical development. It is important to conduct research in broader groups on new interventions that will help reduce stress before a gynecological visit. Medical professionals should take into account patients’ perspectives and allow for collaboration to ensure an individualized approach during gynecological examinations.

**O50. Evaluating the Impact of Physiotherapy on Behavioral and Emotional Well-Being in Girls and Boys with Autism and ADHD**

**M. Stavropoulou ^1^, A. Hristara-Papadopoulou ^1^, M. Kyriakidou ^2^, P. Iakovidis ^1^, A. Halkia ^1^, I. Xinias^3^, V. Dafoulis ^4^, O. Papadopoulou ^1^**

^1^  Department of Physiotherapy, Faculty of Health Sciences, International Hellenic University, Thessaloniki, Greece
^2^  University of Peloponnese, Tripoli, Greece
^3^  3rd Pediatric Department at the Aristotle University of Thessaloniki, “Hippocration” General Hospital, Thessaloniki, Greece
^4^  Child Psychiatry Department of “Hippocration” General Hospital, Thessaloniki, Greece

**Introduction:** This research explores the efficacy of physiotherapy in enhancing the mental health of children, girls and boys with neurodevelopmental disorders. Given the complex interplay between physical and mental health, this study aims to elucidate the potential benefits of physiotherapy interventions on the psychological well-being and quality of life in this demographic. By examining the impact of physiotherapy, the findings aim to offer critical insights for healthcare professionals, facilitating the development of more holistic treatment strategies for children and especially girls affected by neurodevelopmental conditions.

**Aims:** The objective of this study is to assess the impact of physiotherapy interventions on the mental health of children diagnosed with neurodevelopmental disorders. Specifically, it aims to evaluate the role of physiotherapy in managing stress, enhancing mood, improving social adaptability, and stabilizing the psychoemotional state of these children.

**Methods**: This study, conducted at the Child Psychiatric Department of the Hippocratic Hospital of Thessaloniki, assessed the impact of physiotherapy on the mental health of children and adolescents aged 6 to 18 years old with autism and ADHD. A total of 117 parents, mainly women completed a questionnaire evaluating their children’s mental state. The study divided participants into two groups: 64 children received a physiotherapy program of 10 sessions, including diaphragmatic breathing, auricular neuromodulation, Jacobson’s relaxation exercises, relaxation positions, and pressotherapy, while 53 children served as a control group. The aim was to explore the physiotherapy’s effectiveness in improving stress management, mood, and psychoemotional well-being.

**Results:** Initial assessments of children with autism and ADHD revealed significant behavioral challenges, including inattention, hyperactivity, and impulsivity, alongside elevated stress, anxiety, and depression levels. Post-physiotherapy intervention, a notable reduction in these behaviors was observed. Specifically, improvements were seen in attention deficit and impulsivity, with stress levels slightly above average but improved. Additionally, anxiety and mood disorders were reported to be below average, indicating a positive impact of physiotherapy on these aspects of mental health.

**O51. LGBTQI+: I Will Not Be Silenced: Ambiguous Genitalia & Intersex Rights**

**S. Tsamis ^1^, A. Pappa ^1^, F. Grozou ^2^, C. Skentou ^2^, A. Vatopoulou ^2^**

^1^  University of Ioannina, Ioannina, Greece
^2^  University General Hospital of Ioannina, Ioannina, Greece

**Introduction:** “Intersex” is an umbrella term used to describe a range of innate bodily variations in sex characteristics that do not fit typical definitions for male or female bodies, such as hormone differentiations, chromosomic anomalies and ambiguous genitalia. Until recently, intersex individuals were forced to undergo involuntary surgical interventions, called “normalizing” procedures, due to the existing societal view that non-conformance with the binary norms is pathologic. With the prenatal technological advances making it able to detect intersex conditions, there are rising concerns for possible eugenic practices. Presently, awareness remains insufficient, perpetuating misconceptions and contributing to the infringement of intersex rights.

**Aims of the Study:** This project endeavors to illuminate the long-term repercussions that prepubertal “normalizing” procedures have on physical, developmental and psychological health. Meanwhile, it aims to examine the legal and ethical dimensions surrounding these individuals and to foster increased awareness and understanding within the medical community.

**Methods:** A narrative synthesis through literature research of the last decade was conducted using papers from Medline with Mesh terms, background notes from the United Nations Human Rights Office of the High Commissioner and the specific—if existent-legal frameworks from countries worldwide.

**Results:** The medical procedures inflicted on intersex individuals affect immensely their physical, psychological and mental health, as they demonstrate increased incidence of osteoporosis, depression and suicidal ideation. A deeper understanding of the hormonal patterns and physical attributes of each individual is needed, in order for the physician to administer the correct treatment, when necessary.

**Conclusions:** Emphasizing on the medical oath “first, do no harm”, the project advocates for legal reforms that acknowledge and protect the physical integrity and rights of this marginalized demographic ensuring that medical procedures align with the principles of autonomy and informed consent.

**O52. Growing Up with DSD**

**S. Kouzouna ^1^, A. Patroni ^2^, A. Vatopoulou ^2^, F. Gkrozou ^3^, C. Skentou ^2^**

^1^  Faculty of Medicine, University of Ioannina, Ioannina, Greece
^2^  Faculty of Medicine, University of Ioannina, Ioannina, Greece
^3^  Department of Obstetrics and Gynaecology, University Hospital of Ioannina, Ioannina, Greece

**Introduction and Aims of the Study:** Disorders of Sexual Development consist of congenital conditions of atypical development of chromosomal, gonadal, or anatomic sex. But how a DSD child grows up in nowadays and what the legal framework is?

**Methods:** A systematic review was carried out, based on data from 2019 up to date, to present an approach of DSD.

**Results:** The first clinical approach of a child with DSD is usually performed by a neonatologist. Nowadays, the crucial problem is the gender assignment after birth, that is defined by different legal frameworks at European countries, so it is important for a pediatrician to have knowledge above DSD to inform the parents. A complete history, clinical examination and a correct diagnostic process are necessary. The purpose is to exclude life-threatening conditions such as malignancies. Furthermore, it is significant to underline the role of gynecologist, because of the surgical necessity and the diagnosis of DSD cases that emerge in puberty. There are three problems that must be addressed by them: amenorrhea, sexual activity, and infertility issues. By following this approach, early surgical procedures can be avoided, beneficial hormonal therapy and normal psychosocial development can be accomplished. The major problem that faces people with DSD are psychosocial issues. Social stigma makes integration difficult in society, affecting the psychological composition of both child and parent. A diagnosis of DSD can be traumatic, demanding a multidisciplinary team to involve in caring of DSD individuals and their families consisting of medical, psychological specialties and legal counsellors with access to ethical advice, providing the best personalized treatment.

**Conclusions:** Rashly gender assignment is considered to have negative aspects in psychosocial development of the child and lead to non—necessary medical practices. To conclude, a holistic management and amendment of the law are needed.

**O53. Is It Really the Clitoris? A Misdiagnosis of Clitoromegaly Due to Excess Prepuce Skin**

**S. Kasioni, M. Tsiriva, L. Vogiatzi-Vokotopoulou, L. Michala**

1st Department of Obstetrics and Gynaecology, National and Kapodistrian University of Athens, Athens, Greece

**Introduction and Aims of the Study**: Atypical genitalia are usually diagnosed at birth and indicate diagnosis of an intersex condition. Clitoromegaly that presents later in childhood, is usually caused by excess androgen production, either due to an arrhenoblastoma of the ovary or the adrenal gland or as an expression of late onset congenital adrenal hyperplasia. Nevertheless, a number of conditions can be falsely interpreted as enlargement of the clitoral gland and will need to be ruled out during initial investigation.

**Methods**: We present a case report of a girl that was referred for investigation of a newly acquired clitoromegaly at age 4.

**Results**: The girl had normal development for her age. There were no signs of breast development or hirsutism. At close inspection, the apparent enlargement was due to excess prepuce skin, which gave the false impression of clitoromegaly. The diagnosis was easily made by gently pulling the excess skin and revealing the normal clitoral gland.

**Discussion**: Clitoromegaly is an alarming sign for the attending paediatrician. Nevertheless, the clitoral tissue should be assessed carefully prior to such a diagnosis. The case presented here had normal external genitalia, with prepuce skin giving the impression of enlargement. No further investigation was necessary. Several other conditions, such as neurofibromatosis, epidermoid cysts, hemangiomas, intradermal melanocytic nevus could lead to enlargement of the clitoris, usually causing asymmetric changes. A uniformly enlarged clitoris on the contrary, is usually accompanies by other signs of virilization and, should trigger prompt investigations to identify the source of excess androgen production (Figure 5).

**O54. Perspectives of Patient’ Concerns in the Transition Difference of Sex Development Clinic: A Cross-Sectional Survey**

**A. Tan, P. Latthe, P. Marks, R. Igbokwe, H. Gleeson**

Birmingham Women’s and Children’s NHS Foundation trust, Birmingham, UK

**Introduction:** The psychological concerns and health of patients with differences of sex development (DSD) has an enormous impact on the treatment outcomes. Therefore, it is important to establish the concerns and expectations that they have from their DSD clinic appointments.

**Aims of the Study:** To establish the concerns and expectations and their association of patients attending DSD clinic appointments.

**Methodology:** Cross-sectional survey of patients attending their index appointment at a tertiary transition DSD clinic. The clinic is run once every 3 months by a gynaecologist, endocrinologist, geneticist with ability to refer to a psychotherapist or psychosexual nurse specialist.

**Results:** Over a 3 year period, 43 patients who were seen in this clinic, completed the cross-sectional survey. The age range was 14–24 years. The ability to have children was the most common concern, with 56% reporting being worried to extremely worried. Having issues at college, university, or work was the least common concern (2%).

With regards to the patients’ expectations, most patients requested to see an endocrinologist (53%), while the lowest was to see a psychologist (19%). Patients who are concerned about having children are more likely to request a consultation with an endocrinologist (OR 7.80, 95% CI 1.69–36.06, *p*-value 0.009). There does not appear to be a significant association between the age of the patient and their concern for having children (*p*-value = 0.113).

**Conclusions:** The ability to have children appeared to be the most important issue for patients attending transition DSD clinics and it is one of the reasons to request a consultation with an endocrinologist. It may be worth considering presence of reproductive medicine doctor in the DSD transition clinic.

**O55. Application of 3D-Tumor Spheroids in Drug Discovery**

**M. Kaviladze, S. Levakov, M. Dzhafarova**

I.M. Sechenov First Moscow State Medical University (Sechenov University), Moscow, Russia

**Introduction**: In these latter days special importance is played to in vitro models based on cell cultures, including multicellular tumor spheroids (MTS) because of the tightening of the requirements for animal experiments. MTS are artificially produced small solid tumors, which are a three-dimensional (3D) model consisting of cancer cells received by taking a biopsy from a cancer patient. 3D cultures of tumor cells overcome the limitations associated with such basic characteristics as volume gradients, growth factors, and metabolites and the presence of necrotic, hypoxic, resting, and proliferating cells.

**Aims**: The aim was to prove the advantage of the 3D model over the 2D model in order to further integrate the in vitro model of MTS into the design of anticancer drugs and to use primary tumor cells in drug screening studies for the implementation of personalized cancer treatment.

**Methods**: In the study, multicellular spheroids generated from a suspension of isolated cells of the immortalized adenocarcinoma cell line MCF-7 of human breast were obtained in the serum. Microcapsules with MTS were incubated in 24-well plates with Methotrexate for 48 h. The control group was presented by the monolayer MCF-7 culture (100,000 cells per well). Quantitative evaluation of the surviving cells was carried out with trypan blue dye in a Fuchs-Rosenthal counting chamber.

**Results**: The survival rate of viable cells in the control group was 2 times less than in MTS with a Methotrexate concentration of 100 nM. Evaluation of the cytotoxic effect of Methotrexate, based on the size of MTS was also made. When Methotrexate concentration of 100 nM, the number of living cells was 65 and 88% for spheroids with size of 150 and 300 μm, respectively, while in the control group this value was only 35%.

**Conclusions:** Compared to 2D cultures, cancer cells in 3D spheroid cultures demonstrate greater resistance to cytotoxic drugs, with the cytotoxic effect of Methotrexate decreasing while MTS size increasing. In this regard, 3D tumor models are a valuable “tool” for cancer research in the context of drug discovery.

**O56. Fertility Preservation in Childhood Cancer Survivors Previously Treated with Chemotherapy—Is It Effective?**

**M. Shapira, M. Safrai, L. Levi, R. Orvieto, D. Meirow**

Fertility Preservation Center, IVF Unit, Sheba Medical Center, Tel Aviv, Israel

**Introduction:** Chemotherapy can partially destroy ovarian follicle reserve, adversely affecting reproductive potential. Fertility preservation (FP) at cancer diagnosis can be accomplished using either ovarian tissue cryopreservation (OTC) or oocyte storage (OS), depending on patient’s pubertal status and emotional maturity. However, FP occasionally cannot be performed due to oncologic time constraints or critical medical condition. Alternatively, after recovering from cancer and experiencing menarche, non-sterilized cancer survivors can be offered ovarian stimulation for OS.

**Aim:** Evaluate the efficacy of ovarian stimulation in childhood cancer survivors previously exposed to chemotherapy.

**Methods:** This study involves childhood cancer survivors who were followed in the FP unit between 2015–2022. OS was discussed once laboratory/sonographic evidence for impaired ovarian reserve emerged. Patient’s characteristics and stimulation outcomes were retrospectively collected.

**Results:** A total of 21 childhood cancer survivors underwent ovarian stimulation for OS. Mean age at cancer diagnosis was 12.4 ± 6.4 as opposed to 21.69 ± 3.49 at post-chemotherapy FP. Mean FSH levels were 13.4 ± 5.51 pmol/mL and median AMH levels were 0.55 (IQR 0.16–1.04) ng/mL. First stimulation attempt lasted 9.81 ± 2.22 days, with a mean gonadotropins dose of 3246 ± 1057 IU reaching a median of 3708 pmol/mL (IQR 1379–6971) maximal E2. A median of 5 oocytes (IQR 1–14) and 3 mature oocytes (IQR 1–11) was recorded. Eight patients underwent only one stimulation cycle, while others had repeated cycles-7 (2–3 cycles) and 6 (>3 cycles). Median of total cumulative oocytes and M2-oocytes retrieved in repeated cycles was 17 (IQR 15–23) and 11 (IQR 3–17), respectively.

**Conclusions:** Despite a young age at FP, post-chemotherapy childhood cancer survivors perform poorly and often require repeated cycles for a decent chance of future pregnancy. FP at cancer diagnosis should therefore be performed whenever possible. After recovery, FP via OS should be considered as early as adolescence, as any delays may further compromise stimulation outcomes.

**O57. Group Support for Families of Girls with Turner Syndrome—A Pilot Study**

**M. Shemesh-Iron ^1^, N. Gruber ^1,2^**

^1^  Pediatric Endocrine and Diabetes Unit, Edmond and Lily Safra Children’s Hospital, Sheba Medical Center, Tel-Hashomer, Ramat Gan, Israel
^2^  School of Medicine, Tel Aviv University, Tel Aviv, Israel

**Introduction and Aims of the Study:** Parents of girls with Turner syndrome (TS) have to cope with various challenges associated with caring for a child with a chronic disease. We aimed to explore the logic and importance of group support for parents of children with TS.

**Methods:** Group support sessions for parents of girls with TS were divided into meetings regarding psychological aspects, medical aspects, and sociological aspects. Questionnaires about how the families perceive TS and what obstacles they are facing were given before and after the group support through the Mentimeter application. Questionnaires were given at the end of the group sessions to assess efficacy and satisfaction.

**Results:** Parents of 18 girls, with a median (interquartile range) age of 9 (5.5, 12) years old, participated in the group (Figure 6), 56% with 45X0 karyotype. On average 72% of the participants said that the sessions much/very much contributed, and 10% said that the sessions hardly contributed (*p* < 0.05). At the end of the sessions, parents shared that they felt more confident, less isolated, and gained more knowledge regarding TS (Figure 7). At the end of the support group, fifteen (83%) versus 3 (17%) said they would like to continue the meetings of the support group (*p* = 0.0001).

**Conclusions:** Group support equips parents to better care for their girls with TS, a chronic condition, by diminishing feelings of isolation, creating a secure environment for self-expression, and delivering valuable information.

**O58. “No-One Is Familiar with It… I Am Completely on My Own” a Qualitative Study of Parents’ Perceptions of Mayer-Rokitansky-Kuster-Hauser Syndrome**

**S. Carroll ^1,2,3^, E. Salisbury ^2^, N. Deegan ^1,3^**

^1^  Psychology Department, Children’s Health Ireland at Temple Street, Dublin, Ireland
^2^  Psychology Department, IoPPN, King’s College, London, UK
^3^  The Rotunda Hospital, Dublin, Ireland

**Introduction:** Mayer-Rokitansky-Kuster-Hauser Syndrome (MRKH) is characterized by complete or partial absence of the uterus, cervix and upper vagina. Diagnosis of MRKH can be very distressing both for patients and their parents. While the psychological impact of MRKH on patients has been well documented, very little research has explored parents’ experiences of MRKH. As parents often play a key role in supporting their daughters with MRKH and remain involved in their care beyond adolescence, understanding their experiences is important so that appropriate support can be provided in clinical practice.

**Aims:** To explore parents’ responses to their daughters’ MRKH diagnoses, and their perceptions of healthcare and support for MRKH.

**Methods:** The design was qualitative, using In-depth, semi-structured interviews conducted via Microsoft Teams. Purposive sampling was used to recruit nineteen parents via charity websites and social media platforms. Informed consent was obtained from all participants. Interviews were audio-recorded, transcribed verbatim, and analysed using inductive thematic analysis.

**Results:** Five themes were identified, including: (1) varied paths to diagnosis, which highlighted the long delays some families experienced in accessing specialist care; (2) difficulty accessing support, in which parents spoke about not knowing where to turn to for support; (3) navigating involvement in care, in which parents reflected on grappling with how much to be involved in their daughter’s hospital appointments and decisions about treatment; (4) guilt, highlighting that some parents blamed themselves for their daughters’ diagnoses, and (5) loss, in which parents shared the grief they felt both for themselves and their daughters.

**Conclusions:** Adjustment to MRKH is challenging for parents, and better support is needed in clinical practice. Education of non-specialist healthcare providers may help to foster speedier access to specialist care for patients and parents. Parents should be signposted to charities and specialist services that offer peer and/or professional psychological support for MRKH.

**O59. Importance of Renal Ultrasonography in the Investigation of Müllerian Anomalies**

**R. Marozas ^1^, A. Cekuolis ^2^, Z. Bumbuliene ^2^**

^1^  Centre of Radiology and Nuclear Medicine, Vilnius University Hospital Santaros Klinikos, Vilnius, Lithuania
^2^  Vilnius University, Faculty of Medicine, Institute of Clinical Medicine, Clinic of Obstetrics and Gynecology, Vilnius, Lithuania

**Introduction and Aims:** Müllerian (paramesonephric) ducts are paired embryologic structures that undergo fusion and resorption in utero to give rise to the uterus, fallopian tubes, cervix, and upper two-thirds of the vagina. Interruption of normal development of Müllerian ducts can result in formation of Müllerian duct anomalies (MDAs).

**Materials and Methods:** Obstructed hemivagina and ipsilateral renal anomaly (OHVIRA), known as Herlyn-Werner-Wunderlich (HWW) syndrome—very rare congenital anomaly of genitourinary system and is usually identified after menarche. We present a clinical case of 8 year girl from Vilnius University Hospital Santaros Klinikos with diagnosis of OHVIRA syndrome based on radiological examination combining ultrasound and magnetic resonance imaging (MRI).

**Results and Discussion:** HWW cases are diagnosed between 13 and 25 years, the most common symptoms are lower abdominal pain, dysmenorrhea, tender vaginal mass. Our patient complained of urinary tract infection.

MDAs are broad and complex spectrum of abnormalities that are often associated with primary amenorrhea, infertility, obstetric complications, endometriosis. Also MDAs are commonly associated with renal and other anomalies; thus evaluation of kidneys and pelvic organs is important.

**Conclusions:** Clinicians and radiologists should consider OHVIRA in genital tract anomalies. When a genital malformation is identified, uropoetic system should always be screened. And visa versa—if a uropoetic anomaly is diagnosed, genitals should be evaluated for possible anomalies.

**O60. Large Fibroepithelial Polyp of Uterine Cervix and Ovarian Gynandroblastoma in an Adolescent with Dicer1 Syndrome**

**I. Evruke ^1^, O. Dural ^1^, H. Saygili ^1^, M. Yildiz ^2^, A. Aslanger ^3^, H. Ulusoy ^1^, S. Akhan ^1^, F. Baş ^2^**

^1^  Department of Obstetrics and Gynecology, Istanbul University, Faculty of Medicine, Istanbul, Turkey
^2^  Unit of Pediatric Endocrinology, Istanbul University, Faculty of Medicine, Istanbul, Turkey
^3^  Department of Medical Genetics, Istanbul Faculty of Medicine, Istanbul University, Istanbul, Turkey

**Introduction:** Gynandroblastoma is a rare subtype of sex-cord stromal tumors (SCSTs) with morphological evidence of both female and male sex cord differentiation and is known to be associated with *DICER1* mutations. We describe a case of large protruding fibroepithelial polyp of uterine cervix and ovarian gynandroblastoma in a 15 year-old girl as a manifestation of the *DICER1* syndrome.

**Case:** A 15-years-old girl presented with a mass protruding from the vagina. She also had a history of irregular menstrual periods, excessive hair growth and acne since menarche. On physical examination, she had mild hirsutism, severe inflammatory acne with no signs of virilization. The inspection of vulva revealed a large polyp-like mass protruding from the vaginal introitus and otherwise normal external genitalia (Figure 8). Laboratory investigations revealed moderately elevated testosterone levels around 1.1 ng/mL. Pelvic ultrasound revealed a approximately 8 cm multicystic complex adnexal mass on the right. Magnetic resonance imaging (MRI) also showed multicystic-solid complex adnexal mass in the right ovary with marked contrast enhancement without any other abdominal lesion. Serum tumour markers were normal. Laparoscopic right salpingo-oophorectomy was performed following peritoneal washing and meticulous inspection of the contralateral ovary and the peritoneal cavity (Figure 9). The right ovary was removed through a small abdominal incision with using an Alexis retractor without any damage to the ovarian capsule. Histopathologic examination revealed FIGO stage 1A gynandroblastoma of the right ovary. Adjuvant chemotherapy was not indicated due to early stage of the disease. Genetic testing revealed a *DICER1* mutation.

**Conclusions:** This report highlights the importance of investigation for hormone-secreting tumors in the presence of high testosterone levels, even if there is no sign of virilization. This rare presentation also reminds us of screening for a germline *DICER1* mutation in the presence of the early-onset features of this syndrome.

**O61. Is It Necessary to Assess Kidneys During Pediatric Pelvic Ultrasonogrpahy?**

**A. Čekuolis, R. Marozas, Z. Bumbuliene**

Clinic of Obstetrics and Gynaecology, Faculty of Medicine, Institute of Clinical Medicine, Vilnius University, Vilnius, Lietuva

**Introduction and Aims:** Urogenital system develops from the same embryological bundle. The disorders of formation of one system possibly involves the other one. Assessment of uropoetic system, especially kidneys, is highly recommended for evaluation during pelvic ultrasonography, especially for girls and adolescents.

**Materials and Methods**: To show the principles of evaluation of renal ultrasound in Vilnius University Hospital Santaros Klinikos. This system lets to evaluate kidneys quickly and distinguish between normal and pathological conditions.

**Results and Discussion:** The main ultrasonic features of normal kidney include:

1. Smooth or slightly lobulated contour
2. Renal sliding while breathing
3. Localisation: at the shadow of the 12′th rib
4. Both kidneys visible: neither solitary nor duplicated
5. Corticomedullar differentiation: renal pyramids visible hypoechoic compared to cortical layer
6. Echogenicity of cortical layer is less (sometimes equal), but not exceeding that of liver. Note: in newborns (especially premature) it can be higher than liver’s.
7. No pyelocaliceal dilatation.
8. No masses in the kidneys—neither cystic nor solid.

Any mismatch to these features should be evaluated as possible anomaly or pathology and the patient should be referred to more thorough evaluation.

**Conclusions:** Kidney ultrasound during the pediatric pelvic ultrasonography can be done quickly, reliably, and provide a lot of data as a screening test. We recommend to do this exam on every patient.

**O62. Polish Mothers and Cervical Cancer Prophylaxis. What Do They Know and What Attitude Do They Have Towards HPV Vaccination?**

**K. Matusiak, M. Florea, M. Walewska-Wolf**

Children’s Memorial Health Institute, Warsaw, Poland, Warsaw, Poland

**Introduction and Aim of the Study:** Cervical cancer is the fourth-leading cause of cancer-related mortality among women worldwide as well as one of the most preventable human cancers. The aim of the study was to assess the knowledge and awareness of cervical cancer prophylaxis among our patients’ mothers, their attitude towards HPV vaccination and identify sources of information reliable for them.

**Methods:** During patients’ appointments at Children’s Memorial Health Institute PAG Clinic we asked 234 mothers (aged <20–60) to complete a self-prepared questionnaire. The data were analyzed using t-student test and chi square test.

**Results:** Only 61.5% of respondents correctly indicated the purpose of cervical cytology. Similarly 67.9% were convinced that it should be performed once a year and 79.4% claimed that till the end of life. Although 87.6% of answerers have heard about HPV vaccination, still 69.3% of them were willing to get more information. Physicians were considered as the most reliable source of knowledge about HPV vaccination and had relevant impact on mothers’ decision to vaccinate (*p* < 0.05). Nearly all respondents (92.7%) weren’t vaccinated against HPV, still 57.3% declared willingness to vaccinate their children. The opponents indicated that they had too little information (59%), feared of side effects (45%) and doubted in HPV vaccine effectiveness (23%). Almost half of respondents (46.2%) identified HPV vaccination as recommended for both sexes. Mothers >40 yr and with higher education had greater knowlegde about cervical cancer prevention (*p* < 0.05) and were more willing to vaccinate their children (*p* < 0,05). More educated interviewees advocated for obligatory HPV vaccination (*p* < 0.05).

**Conclusions:** Considering that cervical cytology was invented 100 years ago, knowlegde about this screening test is low. The HPV vaccine is well-recognized and well-accepted, but most of respondents indicated the need of information. Therefore, it is crucial to initiate multidirectional educational actions raising awarness about cervical cancer and it’s prophylaxis.


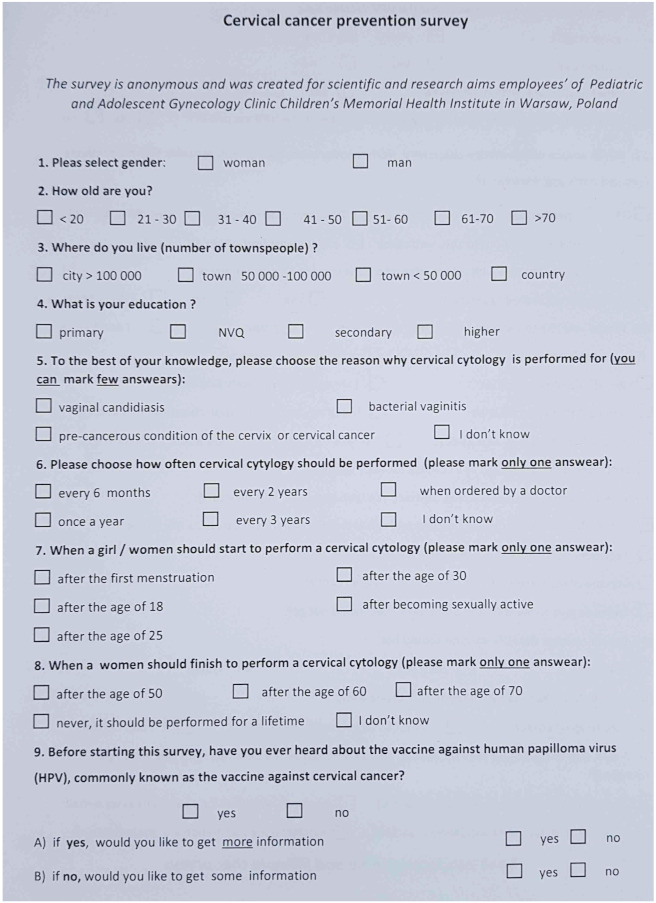



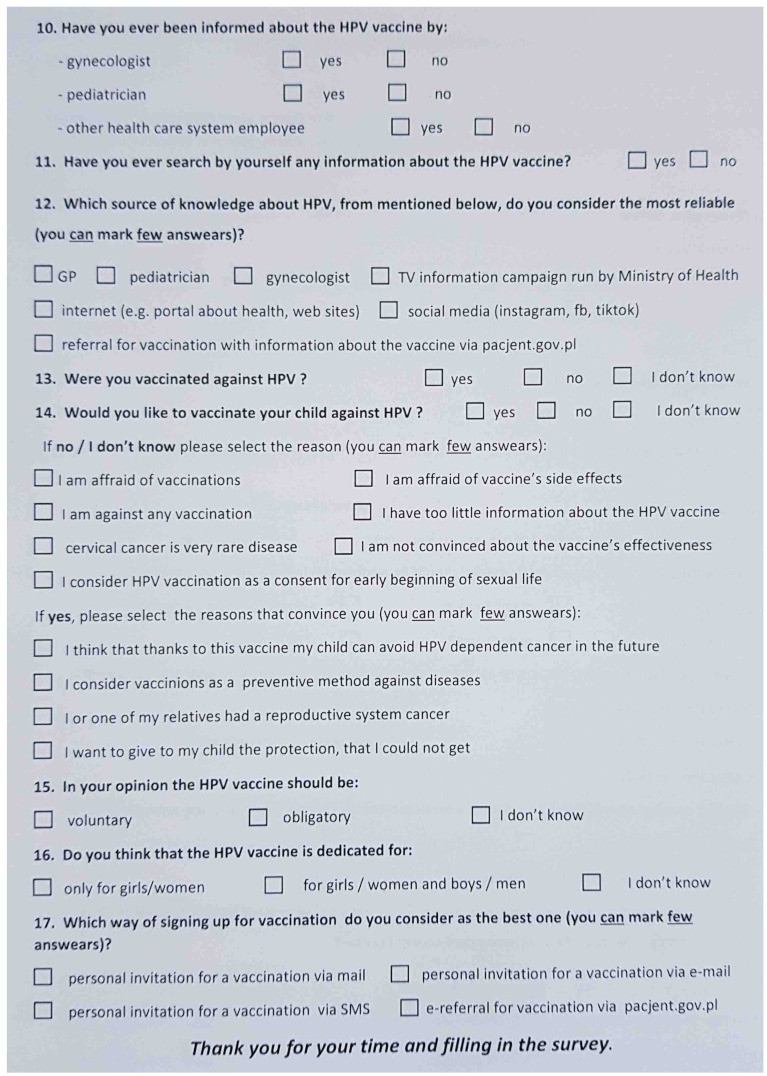

**O63. Safety and Tolerability of the Estetrol/Drospirenone Contraceptive Pill in Starters Compared to Switchers**

**F. Fruzzetti ^1^, T. Piltonen ^2^, K. Gemzell-Danielsson ^3^, A. Black ^4^, F. Gobbi Amorim ^5^, C. Bouchard ^6^, M. Chen ^7^, J. Foidart ^8^**

^1^  Department of Obstetrics and Gynecology, Pisa University Hospital, Pisa, Italy
^2^  Department of Obstetrics and Gynecology, Medical Research Center Oulu, Research Unit of Clinical Medicine, University of Oulu and Oulu University Hospital, Oulu, Finland
^3^  Department of Women’s and Children’s Health, Karolinska Institutet, and Karolinska University Hospital, Stockholm, Sweden
^4^  Department of Obstetrics and Gynecology, University of Ottawa; and The Ottawa Hospital Research Institute, Ottawa, ON, Canada
^5^  Mithra Pharmaceuticals, Liège, Belgium
^6^  Clinique de Recherche en Santé de la Femme (RSF) Inc., Québec City, QC, Canada
^7^  Department of Obstetrics and Gynecology, University of California, Davis, Sacramento, CA, USA
^8^  Department of Gynaecology and Obstetrics, University of Liège, Liège, Belgium

**Introduction and Aims:** Estetrol 15 mg/drospirenone 3 mg (E4/DRSP) is an effective combined oral contraceptive. E4, a native and selective estrogen, combined with DRSP, ensures a regular bleeding pattern and low rates of adverse events (AE). This study assessed the safety/tolerability of E4/DRSP in starters, true starters, and switchers.

**Methods**: Two phase 3 trials (NCT02817828, NCT02817841) assessed E4/DRSP safety/tolerability over 13 cycles (24/4-day regimen). Participants (EU/RUS: 18–50 years, US/CAN: 16–50 years) with regular menstrual cycles were included. Pooled data analyzed frequency and severity of adverse events.

**Results:** Enrolment comprised 1869 starters (51.5%, 20.2% true new starters) and 1763 switchers. Starters reported 46.1% adverse events, lower than switchers (53.4%, *p* < 0.0001). The majority of events were transient (cycles 1–3). The most common reported AEs among starters compared to true new starters and switchers were headache (5.0% vs. 5.3% and 6.8%), metrorrhagia (4.6% vs. 4.9% and 4.9%), vaginal haemorrhage (3.6% vs. 4.4% and 2.8%), weight increase (3.0% vs. 2.9% and 2.4%), nausea (2.9% vs. 2.9% and 2.2%), viral upper respiratory tract infections (2.5% vs. 1.6% and 4.2%, *p* < 0.01), urinary tract infection (2.4% vs. 1.6% and 2.8%), dysmenorrhea (2.3% vs. 1.4% and 4.0%, *p* < 0.001) and acne (2.0% vs. 1.5% and 5.1%, *p* < 0.0001). Treatment discontinuation due to AEs was 9.1% for starters and 10% for switchers, with metrorrhagia being the primary reason. Serious AEs (SAEs) were reported by 24 (1.3%) of starters, 9 (1.2%) true new starters, compared to 17 (1.0%) of switchers. Three SAEs in starters were possibly treatment-related (VTE, acute pancreatitis, and worsening of depression in true starters).

**Conclusions:** E4/DRSP starters and true new starters exhibited lower adverse event rates compared to switchers. Dysmenorrhea, viral upper respiratory tract infections, and acne were more frequent in switchers. This data further supports the favorable safety profile of E4/DRSP in the starter group.

**O64. Menstrual Suppression for Catamenial Anaphylaxis in Adolescents and Young Adults: A Case Series**

**D. Moussaoui, T. Foran, S. Richards, S. Grover**

^1^  Department of Paediatric and Adolescent Gynaecology, The Royal Children’s Hospital Melbourne, Parkville, Australia
^2^  Geneva University Hospitals, Geneva, Switzerland

**Introduction:** Catamenial anaphylaxis describes recurrent anaphylactic episodes occurring at the time of menstruations, which is distinct from “progestogen hypersensitivity” in terms of pathophysiology and presentation. There is no consensus regarding management, but hystero-salpingo-oophorectomy has been described as effective at resolving symptoms in adults. Presentation, disease’s course and optimal management are poorly understood in adolescents.

**Aims:** The aim of this study was to describe the clinical presentation, management and outcome of adolescents and young adults with catamenial anaphylaxis.

**Methods:** We conducted a retrospective chart review of adolescents and young adults (aged less than 30 years) who presented with catamenial anaphylaxis at our institution and at the senior author’s private practice between 1 January 2001 and 31 December 2022. Follow-up ended on 31 December 2023.

**Results:** Seven patients were included. Anaphylaxis symptoms started on day 1–3 of the menstrual cycle or up to one day before the bleeding. Individuals would receive adrenalin on most episodes and be admitted to the hospital. Menstrual suppression was initiated using either a continuous regimen of the combined oral contraceptive pill, norethisterone acetate, or levonorgestrel intrauterine device. Efficacy of menstrual suppression was directly related to improvement in anaphylaxis manifestations, with episodes of breakthrough bleeding associated with relapse in allergic symptoms.

**Conclusions:** In this case series, we found that menstrual suppression using hormonal methods was effective at reducing allergic symptoms related to menstruations. Catamenial anaphylaxis is almost certainly underdiagnosed, and we argue that menstrual history should be explored in adolescents with allergic reactions. Greater awareness about catamenial anaphylaxis could improve its identification and management.

**O65. Study on Menstrual Health: Knowledge, Attitudes, and Practices, Amongst Secondary Schoolgirls Attending a Menstrual Health Seminar in in Malaysia**

**Z. Zulkifli ^1^, A. Zainuddin ^1^, E. Loh Sweet Yi ^1^, L. Lim ^1^, N. Nik Mhd. Nor ^1^, A. Ali ^1^, N. Omar ^2^, N. Abdul Ghani ^1^**

^1^  Faculty of Medicine, The National University of Malaysia (UKM), Kuala Lumpur, Malaysia
^2^  Department of Obstetrics & Gynaecology, Faculty of Medicine, Universiti Teknologi MARA, Sungai Buloh, Malaysia

**Introduction:** Menstrual health is a state of complete physical, mental, and social well-being, not merely the absence of disease or infirmity, in relation to the menstrual cycle. In the Paediatrics and Adolescent Gynaecology (PAG) unit at the Faculty of Medicine, the National University of Malaysia (UKM), many patients with menstrual disorders present late due to inadequate knowledge of menstrual health.

**Aims:** The aims of this study was to determine the menstrual characteristics, knowledge, attitude and menstrual hygiene practices (KAP) of schoolgirls in Cheras, Kuala Lumpur, Malaysia.

**Methods:** A KAP questionnaire of menstrual health, adopted from a survey conducted in Nepal by Simavi et al. (2020) was utilized. This was administered during a menstrual health seminar conducted by the (PAG) Unit of UKM in which 380 schoolgirls attended.

**Results and/or Discussion:** Total number of participants was 341 students, with mean age (+SD) of 14.52 ± 1.25 years. The mean age (+SD) of menarche was 11.8 ± 1.06 years (12 students had not yet attained menarche). When asked to define menstruation; the majority (n = 214; 62.8%) defined it as the flow of dirty blood from the body. Only 118, (34.6%) gave the correct definition. All students use disposable sanitary pad during menses and 145 (42.5%) disposed the pads in the rubbish bins. Majority (n = 256, 75.1%) were able to discuss menstruation with family members. Most (n = 303, 88.9%) were taught on menstruation in their schools. Majority (n = 298; 87.4%) agreed that menstruation is a sign of good health. Most of them (n = 269; 78.9%) agreed that menstruation should not be embarrassing for girls.

**Conclusions:** Although their definition of menstruation was not accurate, the majority of schoolgirls had otherwise good knowledge, practice and attitude towards menstrual health.

**O66. Glycemia and Insulin Need Do Not Vary in Proximity to Self-Reported Menstrual Cyels in Adolescens with Type 1 Diabetes Mellitus**

**A. Priebe ^1,2^, T. Hilyard ^1^, S. Tsai ^1,2^, A. Lawson^1,2^, R. McDonough ^1,2^**

^1^  Children’s Mercy Kansas City, Kansas City, MO, USA
^2^  University of Missouri Kansas City, Kansas City, MO, USA

**Introduction & Objectives:** T1D can change the glycemia and insulin needs of menstruating people living with diabetes. Hormonally induced insulin resistance is thought to play a role in this process. This study evaluates if current treatment methods are associated with differences in continuous glucose monitor (CGM) recorded glycemia and insulin requirement with relation to the timing of self-reported last menstrual periods (LMP).

**Methods:** Adolescent girls with T1D aged 10–18 years and who were connected to a Midwestern Pediatric Diabetes Center Clarity account, were eligible for inclusion. A standard review of systems, which includes a self-reported LMP, is administered to all ambulatory patients across the organization. All patients self-reporting any LMP from November 2018 to August 2023 were included. CGM and insulin administration data were extracted from Dexcom Clarity, Glooko, CareLink, and Tandem t:connect, and were analyzed in relation to the date of the LMP.

**Results:** During the study 689 unique patients reported a total of 1222 menstrual cycles. Inclusion required having device data for the 7 days prior and the 7 days following reported LMP. Ambulatory glucose profile metrics and insulin administration were compared for 7 days preceding and 7 days following the start of the self-reported LMP. There were no statistically significant differences in any of the CGM or insulin metrics (Table 4).

**Conclusions:** In this retrospective study, CGM metrics and insulin requirements were not statistically or clinically different in the 7 days prior to and the 7 days following a self-reported LMP. Prospective future studies are needed to evaluate the effects of advanced insulin delivery systems on glycemia in proximity to menstrual cycles.

**O67. Associations Between Exposure to Violence and Menstrual Health Among Polish Menstruating Adolescents—Results from Polka 18 Study**

**M. Drejza, K. Rylewicz, G. Łopiński, J. Barwinska, E. Majcherek, G. Jarząbek-Bielecka**

Cambridge University Hospitals NHS Foundation Trust, Cambridge, UK

**Introduction and Aims of the Study:** Evidence suggests that being exposed to Adverse Childhood Experiences (ACEs) such as violence is associated with poorer overall health outcomes including poorer mental health and chronic pain. Evidence on the influence of exposure to violence in childhood and adolescence on menstrual health is limited. This study is to investigate how exposure to violence is associated with menstrual health outcomes such as menorrhagia and dysmenorrhea.

**Methods:** This research is part of the POLKA 18 study, a youth-led cross-sectional study aimed at assessing the knowledge, attitudes and practices of Polish adolescents regarding their health and healthcare, with a special focus on sexual and reproductive health, conducted between April and December 2019. Study was funded by the European Society of Contraception and Reproductive Health. Final year high school students were surveyed using self-reported paper questionnaires. The analysis was conducted in the RStudio environment and the *p*-value of less than 0.05 was considered statistically significant.

**Results:** Results from 1545 surveys of menstruating adolescents were taken for the final analysis. 31.7% respondents reported having experienced any form of violence at school, 15.5% sexual violence, 6.5% physical intimate partner violence and 19.5% psychological partner violence. Respondents who experienced school violence reported more painful (*p* = 0.02) and heavier periods (*p* < 0.001) than those who did not. There was also a significant association between having experienced sexual violence, both physical and psychological intimate partner violence (IPV) and both more menorrhagia and dysmenorrhea.

**Conclusions:** Results suggest association between menstrual pain and menorrhagia and exposure to violence among menstruating adolescents. More investigations and analysis for potential moderators and confounders of these associations are needed.

**O68. Adding Norethisterone to Continuous Combined Oral Contraceptive Treatment in Adolescents with Persistent Abnormal Uterine Bleeding: Does It Play a Role?**

**I. Evruke, O. Dural, I. Tas, H. Ulusoy, H. Saygili, C. Yasa, F. Gungor Ugurlucan, S. Akhan**

Istanbul University, Faculty of Medicine, Istanbul, Turkey

**Introduction:** Heavy menstrual bleeding (HMB) is a common condition among adolescents, and COCs containing 30–50 mcg ethinyl estradiol as hormonal therapy are most commonly used as the initial treatment.

**Aim of the Study:** To evaluate whether adding norethisterone (NET) to the continuous COC treatment in adolescents with persistent AUB would be effective.

**Methods:** The study is a prospective study of 16 adolescents aged 10 to 18 years who presented to a tertiary gynecology clinic between 2022 and 2023 with persistent AUB despite being started on a continuous COC regimen due to HMB and were administered NET 10 mg/day for ten days then stepping down to 5 mg/day in addition to the existing therapy. Patients were divided into three groups according to the indications of NET therapy: (i) with recurrent AUB after stepping down the COC regimen to once a day (group 1), (ii) with diagnosed bleeding disorders (group 2), (iii) with increased endometrial thickness (group 3) (Table 5).

**Results:** The mean age at the onset of symptoms was 13.7 ± 2.2 years. Adding NET to existing therapy appeared to cessate the AUB symptoms in all three groups within 24 h. Groups 1 and 2 contained 8 and 5 patients, respectively. Group 3 had 3 patients who had continuous spotting and a mean endometrial thickness of 29.6 ± 8.6 mm. They had 5 mm endometrial thickness at 3 months follow-up after treatment. No additional side effects were reported.

**Conclusions:** Our findings suggest that adding oral NET in all three indications appeared well-tolerated and efficacious in the management of adolescents with persistent AUB.

**O69. Child Sexual Abuse: The Effect of Education on Knowledge, Attitudes, and Perception**

**A. Kalampalikis ^1^, A. Soldatou ^2^, A. Panos ^2^, K. Katsos ^3^, C. Spiliopoulou ^3^, E. Koutsoukou ^4^, L. Michala ^1^**

^1^  1st Department of Obstetrics and Gynaecology, National and Kapodistrian University of Athens, Athens, Greece
^2^  2nd Department of Pediatrics, National and Kapodistrian University of Athens, School of Medicine, Children’s Hospital of Athens “P. & A. Kyriakou”, Athens, Greece
^3^  Department of Forensic Medicine and Toxicology, National and Kapodistrian University of Athens School of Medicine, Athens, Greece
^4^  Department of Social & Education Policy University of Peloponnese, Korinthos, Greece

**Introduction and Aims of the Study:** Research on abuse has shown that the lack of knowledge and awareness can lead to missed opportunities in recognising and reporting cases. We aimed to assess the effect a virtual educational intervention can have on knowledge, attitudes, and perceptions of professionals on Child Sexual Abuse (CSA).

**Methods:** The study population consisted of professionals and students from Greece. A three-hour educational tool covering the interview, examination, management, and legal aspects of CSA with an optional two-hour practice session with clinical case examples was created. A questionnaire assessed their knowledge, confidence in recognising and managing CSA cases, colleague support, faith in services, willingness to be involved, and demographics before, immediately after, and six months after the intervention. Cronbach alpha was used to assess the consistency of the questionnaire. The Wilcoxon Signed-Rank Test and regression analysis were used to estimate the effect of the intervention.

**Results:** In total, 158 participants completed all three questionnaires and 604 participants the first two. The questionnaire was deemed reliable, with a Cronbach alpha of 0.79.

The mean age of the participants was 36.1 (SD = 11.8). The majority identified as females (86.9%). Of them, 0.8% were gynaecologists, 14.2% paediatricians, 1.3% forensic doctors, 9.9% social workers, 13.6% mental health care professionals, 18.5% midwives or nurses. Only 2.6% of the population had regular interaction with suspected victims of abuse (2.6%).

There was improvement in all categories post-seminar when compared to pre-seminar. There was improvement in all categories 6 months post-seminar when compared to pre-seminar, except for confidence. Faith in services and willingness to be involved continued to improve 6 months post-seminar.

**Conclusions:** Educating professionals on CSA can affect their attitudes toward CSA cases and consequently how they respond to them. Continuous education is important to maintain the same level of confidence as highlighted by the attenuation of the effect over time.

**O70. Sex and the “Ovaries”, Allies in the Psychosocial and Sexual Life of Women with Premature Ovarian Insufficiency and Turner Syndrome**

**E. Geramani, M. Tsiriva, A. Rouvali, L. Vogiatzi, S. Roidi, S. Ivanidou, L. Michala, G. Dascalakis**

First Department of Obstetrics and Gynaecology, Alexandra Hospital, Athens, Greece

**Introduction and Aims of Study:** The aim of the study is to evaluate the psychosocial/sexual development of young women with Turner syndrome (TS) compared to women of the same age who were diagnosed with premature ovarian insufficiency (POI) during adolescence.

**Material and Methods**: We retrospectively reviewed case notes of women with TS and POI, whose diagnosis had been made before age 18 and who were under regular follow up in a tertiary department of Paediatric and Adolescent Gynaecology. We recorded demographics, age of coitarche and level of education. Cases of iatrogenic POI were excluded.

**Results**: 11 women with TS and 16 women with POI were identified as eligible for this review. All women had completed a full cycle of basic education. Five (31.5%) women with POI (31.5%) and 4 (36.3%) attended or had completed higher education courses. In addition 45% of TS and 44% of POIs were sexually active. Age at coitarche was 18.5 years and 19.5 years respectively.

**Conclusions:** In this sample, women with TS and POI had similar adaptation in their social development/education. Also, the number of women who did not start their sexual life by the age of 24 was similar in both groups. There was however a slight delay in sexual debut of women with TS, which could be attributed to delayed puberty. Further studies to elaborate whether additional factors, such as low stature and intensive medical monitoring, affects sexuality are required.

**P01. Challenges and Peculiarities Regarding Contraception Among Disabled Persons. A Literature Review**

**P. Papadopoulou ^1^, G. Porfyri ^2^**

^1^  MSC “Premalignant Diseases in Gynecology”, Faculty of Medicine, Aristotle University of Thessaloniki, Thessaloniki, Greece
^2^  National and Kapodistrian University of Athens, Faculty of Medicine, MSC “Global Health-Disaster Medicine”, Athens, Greece

**Introduction and Aims of the Study:** While sexuality is a fundemental characteristic of the identity of every and each individual, people experiencing disability are often demeaned by society, not being considered as being sexually active. This stigmatizing perception represents a significant barrier for their access to the healthcare system, resulting to an increased risk of sexually transmitted infections. The aim of this study is to illustrate the challenges faced by people with disability in regards to contraception, to identify the peculiarities that must be taken in cosideration concerning the different contraception methods and in regards to each category of disability and finally to promote a healthy sexuality for this community.

**Methods:** A review of 31 articles from 2010 to 2023—on PubMed and Google Scholar—regarding the use of contraception among disabled persons.

**Results:** According to research, people with emotional, sensory, developmental or physical disabilities tend to engage in high-risk sexual activity, due to low levels of contraceptive knowledge. As a result, they are at increased risk for sexually transmitted infections, including HIV. People with intellectual disabilities facing various self-care limitations, use less effective contraception tactics such as condoms and withdrawal method, while intrauterine device constitutes the safest and most beneficial type of contraception because of limited side effects. Socioeconomic and health factors such as literacy, age, earnings and functional impairment seem to play a significant role on the use of the different contraception methods.

**Conclusions:** The development of a healthy sexual conduct indicates a pre-existing guidance as well as an appropriate education. Clinicians, occupational therapists as well as family members should be the major source of information for people with disability. Health professionals should prioritize sexual health of disabled persons and de-stigmatize this topic.

**P02. Motherhood and Disability: A Challenge for Patients and Physicians**

**G. Porfyri ^1^, P. Papadopoulou ^2^, K. Porfyris ^3^**

^1^  National and Kapodistrian University of Athens, Faculty of Medicine, MSC “Global Health-Disaster Medicine”, Athens, Greece
^2^  MSC “Premalignant Diseases in Gynecology”, Aristotle University of Thessaloniki, Thessaloniki, Greece
^3^  MSC “Global Health and Disaster Medicine”, National and Kapodistrian University of Athens, Athens, Greece

**Introduction and Aims of the Study:** Women with special needs feel the same desire for motherhood as non-disabled women. However, their desire is often accompanied by high risks of medical complications like preeclampsia, diabetes, venous thromboembolism, cesarean delivery, infant low birth weight, preterm birth, neonatal intensive care unit admission and perinatal death. The aim of this review is to underline the barriers that disabled women face regarding motherhood and to give an additional inside look on their experience.

**Methods:** A review of 41 articles from 2000 to 2023 on PubMed and Google Scholar, concerning motherhood among disabled women.

**Results:** Disabled women state that motherhood “provided” them a gender they felt they had lost. Maternity validated their femininity in manners they had never imagined, reproducing emotions of joy, accomplishment, and self-confidence. These feelings were succeeded by sadness and fear when immediate pregnancy terminations were occasionally proposed by physicians insinuating that these women will not appropriately raise their children. Furthermore, mothers constantly struggle with concerns that their baby could be removed by social services. Alongside, data reveal that motherhood requirements are directly affected by the type of maternal disability. Physically disabled mothers struggle with night care routine, bathing and carrying their child, while visually impaired mothers struggle to achieve an in-depth communication with their infant. On the other hand, mothers with intellectual disability make tremendous efforts to be in position to recognize potential health alarming signs during their pregnancy as well as later, on their newborn.

**Conclusions:** Clinicians should focus on identifying the strengths of disabled mothers and carefully guide them to use these strengths for their parental role. Finally, they should realize their duty to advocate for this population, by promoting their right as well as their capacity of being parents.

**P04. Central Precocious Puberty During COVID-19 Pandemic Period**

**M. Fanaki, L. Michala**

1st Department of Obstetrics and Gynecology, “Alexandra” General Hospital, Athens, Greece

**Introduction and Aim of the Study:** Central precocious puberty (CPP) is a condition where the hypothalamus-pituitary-gonadal axis is activated earlier than normal leading to premature development of secondary sexual characteristics before 8 years of age in girls and 9 years of age in boys. Since the commencement of the COVID-19 pandemic, many studies reported a rise in cases of newly diagnosed precocious puberty comparing to the pre-pandemic years. The purpose of this study was to critically and systematically evaluate the literature regarding CPP rise during the COVID 19 pandemic.

**Methods:** We searched PubMed and Google Scholar for relevant articles using the following MeSH terms: ‘central precocious puberty, ‘COVID-19′, ‘early puberty’. We included studies calculating the risk of CPP before and during the COVID-19 pandemic. We excluded studies looking at patients with an identifiable cause for CPP or with Peripheral Precocious Puberty.

The primary outcome was the incidence of central precocious puberty during the pandemic comparing to the pre-pandemic period. We analyzed data regarding anthropometric, biochemical, and pelvic ultrasound data between the two groups.

**Results:** Overall, 16 studies with 2175 subjects were included, of which 1818 were diagnosed with CPP. Our data reported an increase in the number of new diagnoses of CPP during COVID-19 pandemic (985 subjects) compared with the pre-pandemic period (833 subjects). The mean age of diagnosis in the first group was 7.42 years versus 7.54 years in the second group. Notably, CPP developing during the pandemic was associated with a higher Body Mass Index (BMI) compared with the group of the pre-pandemic period (17.50 versus 17.08).

**Conclusions:** The pandemic and lockdowns led to changes in lifestyle habits, social isolation, sleep disturbance, excess screen time, and increased stress levels. We hypothesize that these alterations influenced the rise in frequency of CPP.

**P05. Transformation of Mullerian Papilloma of Uterine Cervix to Clear Cell Adenocarcinoma in a Nine-Year-Old Girl**

**M. Walewska-Wolf ^1^, B. Antoniak ^1^, O. Rutynowska-Pronicka ^2^, B. Dembowska-Bagińska ^2^, K. Felberg ^3^, P. Gastol ^3^, G. Panek ^4^, W. Grajkowska ^5^, M. Pronicki ^5^**

^1^  Out Patient Department of Pediatric Gynecology Children Memorial Health Institute, Warsaw, Poland
^2^  Department of Pediatric Oncology Children Memorial Health Institute, Warsaw, Poland
^3^  Department of Pediatric Urology Children Memorial Health Institute, Warsaw, Poland
^4^  Department of Obstetrics and Gynecology Centre of Postgraduate Medical Education, Warsaw, Poland
^5^  Pathology Laboratory Children Memorial Health Institute, Warsaw, Poland

The girl, 1 year and 3 months of age, had a vaginal bleeding in June 2015 and a cervical polyp was found during a vaginoscopy, from which a biopsy of 1.2 × 0.7 × 0.2 cm was performed with a histopathological result of mullerian papilloma. Due to the benign nature of the lesion, it was decided to keep the girl under observation until she was older.

There was occasional spotting and discharge, which was treated according to the antibiogram. On an ultrasound, a lesion was observed in the vaginal lumen, richly vascularized, approximately 15–17 mm long.

In October 2023, the patient had heavy bleeding. During a vaginoscopy, a polyp was visualized, which was macroscopically all removed. Histopathology revealed HPV independent clear cell type adenocarcinoma of uterine cervix. Additional investigations: pelvic RM, PET and CT chest showed no active “neo” process.

Due to the disease type and its prognosis, it was decided to perform a vaginal cervical amputation and laparoscopic biopsy of the iliac and obturator lymph nodes. No malignant cells were found during postoperative examination.

Our case demonstrated that there is a long-term risk (even after 8.5 years) of transformation from benign mullerian papilloma, to malignant clear cell adeno carcinoma. Mullerian papillomas, rare and benign polypoid lesions of the cervix in young girls, usually occurs between 2 and 5 years of age. The epithelium of the upper vagina is of mullerian origin and has the potential to differentiate into both a glandular and squamous form. It may undergo neoplastic transformation many years later if the excision is incomplete. This neoplastic transformation was described mainly in postmenopausal women. According to our knowledge, this is the first case report of such transformation in a prepubertal girl.

**P06. Isolated Torsion of the Fallopian Tube Associated with Hematosalpinx in a 17-Year-Old Adolescent: A Case Report**

**H. Ziadeh**

Lebanese American University, Beirut, Lebanon

**Introduction:** We describe a case of 17-year-old girl found to have isolated fallopian tubal torsion (IFTT) and hematosalpinx who underwent laparoscopic salpingectomy. IFTT is a rare entity with an incidence of 1 in 1.5 million women. It occurs in reproductive age women and is uncommon in postmenopausal women, in the pediatric population, and in young sexually inactive adolescents.

**Aims of the Study:** To shed light on a very rare condition which is isolated fallopian tubal torsion (IFTT) that we have to consider it in every adolescent girl with severe pelvic pain and adnexal mass.

**Methods:** This report describes a rare case of a 17-year-old girl with IFTT and hematosalpinx.

**Discussion:** A 17-year-old, adolescent presented to the emergency department for severe left lower quadrant pain, irradiating to the left leg associated with nausea, not relieved by intravenous analgesia. On presentation, her physical examination was normal aside from severe tenderness and guarding on the left lower quadrant. Official transabdominal ultrasound showed normal uterus, normal left and right ovaries with no signs of ovarian torsion, and an oval-shaped retro uterine cystic structure measuring 8.4 cm of unknown origin. Pelvic magnetic resonance imaging was started but interrupted due to the patient’s severe pain, which confirmed the presence of a retro uterine cyst independent from both ovaries with a focal retraction suggestive of an adnexal cyst’s torsion. After informed consent was obtained, a decision was made to proceed with explorative laparoscopy. Inspection of the abdomen showed a large left hematosalpinx was identified with a left tubal torsion (triple spin). The left tube was detorted three times and found to be gangrenous, and a left salpingectomy was performed. The pathology report was consistent with a hematosalpinx with features of torsion and no sign of malignancy.

**Conclusions:** In cases of acute severe lower pelvic pain in young adolescents or children, IFTT is an important and challenging diagnosis.

**P07. Retrospective Review on Management of Heryln-Werner-Wunderlich Syndrome (OHVIRA)**

**K. Ng, M. Lee, N. Ip, S. Chan**

The Chinese University of Hong Kong, Hong Kong

**Background and Aim:** Herlyn-Werner-Wunderlich Syndrome, also known as obstructed hemivagina and ipsilateral renal anomaly (OHVIRA), is a rare congenital anomaly, with challenges in diagnosis due to normal menstruation. Treatment involves vaginal septum resection, with hemi-hysterectomy in severe cases. This review analyzes operative outcomes in OHVIRA patients.

**Methods:** This retrospective review was conducted at a tertiary hospital from 2010 to 2023. Patient medical records were reviewed to gather demographic information, presentation details, and surgical outcomes.

**Results:** Twelve cases were identified, with a mean age of 16.7 years at presentation (range: 9–33 years old). Patients with microperforation (n = 5) presented at a significantly older age than those without (n = 7) (26 vs. 13 years, *p* = 0.005). The most common symptoms were dysmenorrhea (50%) and vaginal discharge (30%). MRI was the primary imaging modality (58%), followed by ultrasound (30%) (Figure 10). Haematocolpos was present in all cases, while haematometra was observed in one case. Haematosalpinx occurred in five cases: two resolved spontaneously, two underwent salpingostomy, and one required laparoscopic surgery. Two cases had concurrent infection with a microperforated septum.

All cases underwent surgery within the same year of diagnosis. Eleven patients underwent a single-stage operation with vaginal septum resection (Figure 11), and none experienced recurrence of obstruction during follow-up. A 12-year-old patient had hemi-hysterectomy for reobstruction after initial incision and drainage for haematocolpos at another hospital. The mean distance of the septum from the introitus was 2.7 cm, with a mean septum thickness of 3 mm.

**Conclusions:** OHVIRA is a rare condition that requires expertise in diagnosis and management. Single-stage operation with vaginal septum resection typically yields favorable outcomes.

**P08. Continued Use of Long-Acting Contraceptive Methods in Adolescents in an Ambulatory in the Amazon Region, from 2020 to 2023**

**M. Ribeiro Simões ^1,2^, E. Iglesias Rosa ^2^, D. Adner Ferrari ^3^, L. Gonçalves Vieira ^3^, M. Simões Silveira ^1^**

^1^  Centro Universitário São Lucas—Afya, Porto Velho, Brazil
^2^  Maternidade Municipal Mãe Esperança, Porto Velho, Brazil
^3^  Universidade Federal de Rondônia—UNIR, Porto Velho, Brazil

**Introduction:** The teenage pregnancy rate has remained stable in recent years, as a result of the increase in the use of contraceptives, but has increased in the 12 to 15 age range. Unwanted pregnancies are a result of incorrect or inconsistent use of contraceptives, and not an intrinsic failure of the method. To avoid this error, reversible contraceptive methods must be used with a small difference between efficacy (Pearl index) and effectiveness, the result of which depends less on the user. Examples are subdermal implants and intrauterine devices (IUDs), currently called long-acting reversible contraception (LARC).

**Objectives:** To analyze the continuity of use of longacting contraceptive methods in adolescents in a reproductive planning outpatient clinic in the Amazon Region.

**Methods:** Quantitative research was carried out, through a survey of the contraceptive methods chosen by the adolescents in medical records, and descriptive statistics were used, such as: position measurements, central tendency and dispersion.

**Results:** Between January 2020 and December 2023, 2452 adolescents were served, with an average age of 18.59 years. Of these, 1.235 (50.4%) had the IUD inserted, 380 copper, 212 (8.6%), removed the IUD and 377 (15.4%) Missing. The main reasons for removing the IUD were: 71 (33.5%) poor positioning of the IUD, 69 (32.5%) pain, 45 (21.2%) bleeding, 9 (4.2%) to get pregnant.

**Conclusions:** The greatest benefits of using LARCs in adolescents are the low discontinuation rate of the method and the fact that it does not depend on the user to be effective, as in the case of short-term contraception methods, where some require daily effort that many teenagers are unable to maintain it, which ends up resulting in an unplanned pregnancy. Teenage pregnancy has been considered, in some countries, a public health problem, as it can lead to obstetric complications, with repercussions for the mother and the newborn, as well as psycho-social and economic problems.

**P09. Retrospective Review on Management of Imperforate Hymen**

**Y. Yeung ^1^, K. Ng ^1^, P. Ip ^1^, N. Lee ^2^, S. Chan ^1^**

^1^  Department of Obstetrics and Gynaecology, Prince of Wales Hospital, Hong Kong
^2^  Department of Obstetrics and Gynaecology, The Chinese University of Hong Kong, Hong Kong

**Introduction:** Imperforate hymen is an uncommon, isolated and sporadic congenital anomaly. Its symptoms including lower abdominal pain, acute urinary retention and amenorrhea. The standard treatment is surgical intervention by hymenotomy or hymenectomy.

**Methods:** This is a retrospective review on all patients who attended a tertiary university hospital with the diagnosis of imperforate hymen from 2000 to 2023. The study aims to review the operative outcomes, including restenosis and re-operation. The secondary outcome includes operative complications. Data were collected by reviewing the medical records.

**Results:** In total, 17 patients were identified. The presenting age ranges from 10 to 40, with a mean age of 14.6. One patient, who presented at 40 years old, actually had microperforation of 5 mm and experienced monthly menstruation with perineal mass since menarch. She presented wit suprapubic pain and fever, then had hymenectomy done in the same admission. All patients were post-pubertal at diagnosis. The most common symptom on presentation was acute lower abdominal pain (n = 9, 52.9%). Second common was acute retention of urine (n = 6, 35.3%). Others included cyclical abdominal pain (n = 5, 29.4%), pelvic mass (n = 2, 11.7%) and perineal mass (n = 1, 5.9%). Associated symptoms included difficulty in urination, constipation and primary amenorrhoea. Many had a bluish bulge at vagina (n = 7, 41.2%) and most USG showed hematocolpos (n = 14, 82.3%) Majority underwent hymenectomy (n = 16, 94.1%), except one who had spontaneous perforation after admission with resolution of symptoms and achieved normal menstruation afterwards.

All patients who had undergone operation had resolution of symptoms immediately. All had normal menstruation afterwards and did not recur by the completion of follow up.

No complications were identified after surgery.

**Conclusions:** The outcome of imperforate hymen with operation done is excellent. Simple surgery can provide immediate relief of symptoms with minimal risk of complications and recurrence.

**P10. Recurrent Adnexal Torsions in an Adolescent Patient with Ehlers-Danlos Syndrome: A Case Report**

**M. Panagiotopoulos, M. Tsiriva, N. Kathopoulis, L. Vogiatzi-Vokotopoulou, K. Koukoumpanis, A. Douligeris, A. Protopapas, L. Michala**

Alexandra Hospital, Athens, Greece

**Introduction**: Hypermobile Ehlers-Danlos syndrome (hEDS) is a clinical diagnosis and the most common type of Ehlers-Danlos syndrome (EDS). It is mainly characterized by large- and small-joint hypermobility, frequent joint dislocations and chronic joint pain. Patients may experience a variety of other associated symptoms, including recurrent abdominal pain of uncertain etiology.

**Case Presentation**: A 17-year-old girl, diagnosed with Hypermobile Ehlers-Danlos syndrome (hEDS), had been having multiple episodes of recurrent, lower abdominal pain, attributed to intermittent partial adnexal torsion for the past 8 years. At age 8 and 10, she had two exploratory laparotomies due to acute abdominal pain. At the first surgery, she had a left adnexectomy due to ovarian torsion and appendectomy, while at the second, detorsion and drainage of a right hydrosalpinx with torsion. As these episodes of recurrent adnexal torsion were suspected to be linked with hEDS, she was offered surgical intervention, a laparoscopic right oophoropexy by shortening the utero-ovarian ligament. Postoperatively, she has been asymptomatic for 3 months and is now under close follow-up observation.

**Conclusions:** Patients with hEDS may be susceptible to recurrent adnexal torsions due to associated tissue elasticity. This could explain some cases with chronic abdominal pain, which is present in over half of these patients and is usually attributed to other causes, such as disorders of gut–brain interaction or irritable bowel syndrome.

**P11. Two Successful Pregnancies of a Mc Cune Albright Patient. A Case Presentation**

**V. Triantafyllidi ^1^, M. Tsiriva ^1^, G. Ntali ^2^, N. Machairiotis ^3^, G. Varvarousi ^4^, A. Marogianni ^1^, A. Papailiou ^1^, S. Roidi ^1^, G. Daskalakis ^1^, L. Michala ^1^**

^1^  1st Department of Obstetrics and Gynecology, ‘Alexandra’ General Hospital, National and Kapodistrian University of Athens, Athens, Greece
^2^  Department of Endocrinology, Diabetes and Metabolism, Evangelismos Hospital, Athens, Greece
^3^  Third Department of Obstetrics and Gynecology, University General Hospital “ATTIKON”, Medical School, National and Kapodistrian University of Athens, Athens, Greece
^4^  Department of Anaesthesiology, Alexandra General Hospital, Athens, Greece

**Introduction and Aims:** McCune Albright syndrome (MAS) is a rare genetic disease due to a sporadic postzygotic mutation of the GNAS1 gene, leading to irregular function of endocrine and exocrine glands. Most girls present with precocious puberty, caused by the development of recurrent autonomous ovarian cysts that produce estrogen. After menarche, the majority of patients with ovarian GNAS mutation have menstrual irregularities and infertility. Our aim is to present a rare case of a 31 years old (y.o.) MAS patient who delivered two healthy neonates, following spontaneous conception after unilateral oophorectomy on the affected side.

**Methods:** Case Presentation.

**Results:** We present the case of a 31 years old female, who was followed in our department since she was aged 19 due to recurrent ovarian cysts, which were managed conservatively. The diagnosis of MAS followed a history of precocious puberty and spontaneous fractures in childhood, along with the presence of two café au lait spots and was confirmed with genetic testing. At age 24, she expressed a wish for conception and after a multidisciplinary team consensus, we performed a laparoscopic unilateral oophorectomy on the affected side. She reported regular menses after six months and two years later, she conceived and had two consecutive pregnancies, which led to uncomplicated deliveries by caesarean section at 38 and 37 weeks of pregnancy in 2022 and 2024 respectively. Both neonates were healthy. Despite discomfort in the 3rd trimester of both pregnancies, she did not develop any permanent skeletal problems postnatally.

**Conclusions:** Literature on pregnancy of MAS patients is restricted. Ovarian and endometrial involvement can be responsible for infertility in MAS women. Spontaneous conception and delivery of healthy neonates is possible on these patients with the proper medical management from a multidisciplinary team. Unilateral oophorectomy may be necessary in adulthood, in order to allow resumption of spontaneous ovulation.

**P12. Evaluation of Chatgpt-Generated Content in Response to Girls’ Questions on Puberty and Adolescence Related Concerns: A Perspective on Sensitive Inquiries**

**A. Brus-Chojnicka ^1^, M. Chojnicki ^2^, K. Kapczuk ^3^**

^1^  Outpatient Clinic of Pediatric and Adolescent Gynecology. Gynecology And Obstetrics Clinical Hospital of Poznań University of Medical Sciences, Poznań, Poland
^2^  Department of Immunobiology, Poznań University of Medical Sciences, Poznań, Poland
^3^  Division of Gynecology, Poznań University of Medical Sciences, Poznań, Poland

**Introduction:** Minors often interact with online resources before mastering literacy, seeking information beyond entertainment, including health and maturation queries. This can result in both reassurance and anxiety. Traditionally, information was sourced from websites validated by search engines. However, with the emergence of generative AI technologies such as ChatGPT, there’s now access to instant, albeit potentially unverified, content. This study evaluates ChatGPT’s responses to girls’ common health-related questions, especially regarding puberty. It aims to assess the precision and safety of medical information provided by AI to young females and identify risks associated with minors using this technology without adult guidance.

**Methods:** The authors compiled queries from the autocomplete function of internet search engines, initiated with the phrase “I am (age) years old and…”, targeting the age group of 8–17 years. Ten questions posed by girls, touching on issues of maturation, intimacy, and sexual health, intended for pediatric gynecology specialists, were selected. These inquiries served as prompts for the chatGPT model. Responses were analyzed by physicians specializing in pediatric gynecology, using the Likert scale, assessing the content relevance, adequacy of recommendations, empathy level, and age appropriateness for the inquirers.

**Results:** The analysis showed that the content relevance, appropriateness of recommended actions, and level of empathy demonstrated in responses to all questions were rated as good or very good, achieving 3 or 4 points (Likert scale). Concurrently, concerns were raised regarding the proper age adjustment of the message for the individuals posing the questions.

**Conclusions:** Large language models, including ChatGPT, exhibit the capacity to generate substantive and empathetic responses to inquiries previously posed via internet search engines. The ability to tailor communication to the age of the inquirers necessitates refinement through subsequent prompting.

**P13. Polycystic Ovary Syndrome and Mental Health Co-Morbidities in Adolescents**

**N. O’Neill ^1^, A. Lynn ^1^, M. Zamani ^2^, A. Kuehne ^1^, K. Miclette ^1^, E. Rowe ^1^, L. Fussner ^3^, A. Vash-Margita ^1^, C. Lepore**

^1^  Yale University, New Haven, CT, USA
^2^  Independent Statistician, Washington, DC, USA
^3^  Children’s Hospital of Philadelphia, Philadelphia, PA, USA

**Introduction:** Mental health disorders (MHDs) have been associated with polycystic ovary syndrome (PCOS), but few have studied the effect of PCOS treatments on MHDs in adolescents. This cohort study explored MHD prevalence, effects of hormonal treatment (HT) and 1,25-dihydroxyvitamin D3 (VitD) supplements on MHDs, biochemical characteristics, and ultrasonographic measurements.

**Methods:** This IRB-approved retrospective study included female subjects 12–21 years seen at a tertiary children’s hospital from 2016–2023. Inclusion criteria included meeting two 2023 modified Rotterdam criteria, defined as “at risk” if <8 years from menarche and as having PCOS if ≥8 years post menarche. Exclusion criteria was menstrual age <2 years. MHDs were identified via chart review of clinical notes. Two radiologists re-interpreted ultrasound imaging. Continuous variables were compared using Wilcoxon Rank Sum Test for two groups and Kruskal Wallis Test for more than two groups. Tests of association were Chi-Square or Fisher’s Exact.

**Results:** We included 181 subjects. 25.4% were on VitD, 67.9% were on HT, and 20.4% on both. 40.9% had anxiety, and 32.6% had depression. Fewer patients on VitD compared to no VitD had anxiety (*p* = 0.044). Fewer patients on HT compared to no HT had anxiety (*p* = 0.042). VitD levels were higher in subjects taking any type of HT (26.1 vs. 23.3 ng/mL, *p* = 0.04). Free and total testosterone levels were lower in patients taking HT (*p* < 0.01; *p* = 0.03). There were no differences in other biochemical characteristics or ovarian volume among treatment groups.

**Conclusions:** There was a high prevalence of anxiety and depression among the cohort. We demonstrated a beneficial association between VitD supplements, HT, and anxiety. A possible advantageous use of HT and VitD in patients with anxiety and PCOS warrants further research. This study emphasizes the importance of a multi-disciplinary care model among adolescents with PCOS.

**P14. Paediatric and Adolescent Vulvar Lichen Sclerosus in the Province of Geneva Switzerland, the Situation from 2007 to 2023**

**V. Crofts, D. Moussaoui, M. Yaron**

Geneva University Hospitals, Geneva, Switzerland

**Introduction:** *Vulvar Lichen Sclerosus* (VLS) is an uncommon condition with a chronic course. The average age of VLS onset in girls is 4 to 7 years, and children may present with a wide variety of complaints. VLS is often misdiagnosed, with a diagnostic delay of 1 to 2 years on average, and up to 84% are not diagnosed before seeing a specialist This delay can often lead to suffering and frustration for both the patient and her parents and possible long term psychological consequences following the diagnosis and presence of abnormal vulvar anatomy. There has been paucity of research on VLS in children and most case series or retrospective cohort studies have few children or adolescent patients included.

**Aims:** This study aims at describing the presentation, delay to diagnosis, treatment and outcome of girls with VLS in the province of Geneva, Switzerland.

**Methods:** This is a retrospective descriptive cohort study, based on reviewing medical charts of paediatric and adolescent girls with the diagnosis of VLS before the age of 18.

**Results:** Ongoing analysis of more than 60 patients. Results will provide information on paediatric and adolescent VLS in the province of Geneva Switzerland and most likely contribute to improvement of knowledge and care for this young population.

**Conclusions:** Improving the knowledge on VLS should enable physicians to make timely diagnosis and treatment, while educating patients and their families in order to improve patients’ outcomes.

**P15. Are We Failing Our Teens? An Audit of Contraception Counselling and Provision in Adolescent Patients Attending an Irish Tertiary-Level Unit**

**G. Madigan, S. Al-Tikriti, D. Browne, N. Deegan**

The Rotunda Hospital, Dublin, Ireland

**Introduction and Aims:** A re-audit, to assess the practice of contraceptive counselling and provision for patients attending the Teen Pregnancy Service, following a Quality Improvement Project aimed at improving access to contraception for adolescent patients. The Teenage Pregnancy Service comprises a vulnerable cohort of patients. Our patients are disproportionately affected by social disadvantage, and face significant barriers to accessing care. 86% describe the index pregnancy as unplanned. An audit to assess counselling and provision of contraception within our service was conducted. This audit revealed deficits in contraceptive counselling, particularly in the antenatal period. A QIP to improve access to contraception was conducted.

**Methods:** This was a retrospective analysis of patients attending the Teen Pregnancy Service, who delivered between 1 January 2023 and 31 July 2023. It was a re-audit of a study assessing the period 1 July 2021 to 31 December 2022, completing the audit cycle. Our intervention was two-fold. In the first instance, we aimed for 100% counselling rates in the antenatal clinic. All patients were counselled on postnatal contraception twice; at least once on her own. Written information in the patient’s native language was provided. Additionally, we had an education initiative for the provision of long-acting reversible contraception (LARC). Doctors were trained to insert the Implanon device (sub-dermal contraceptive implant), and Midwives expanded their role to the administration of the Depo-Provera injection (3-monthly contraceptive injection).

**Results and Conclusions:** 50 charts were reviewed. 100% (n = 50) of patients received antenatal counselling on contraception. 58% (n = 29) of patients were discharged home with contraception, an increase from 22%. 58% (n = 29) of patients attended their postnatal visit, an increase from 30%. These results show that antenatal counselling and provision of contraception is acceptable to patients, and provides certainty. By opportunistically counselling patients in the antenatal period, we increase patients’ ability to vindicate their wishes for family size. This model could be expanded to any Obstetric service.

**P16. Prevalence of Sexually Transmitted Infections in Adolescents Assisted at an Ambulatory in the Western Amazon**

**M. Ribeiro Simões ^1,2^, I. Perea Monteiro ^1,2,3^, M. Duque Bessa ^2,3^, S. De Souza Amado ^3^**

^1^  Centro Universitário São Lucas—Afya, Porto Velho, Brazil
^2^  Maternidade Municipal Mãe Esperança, Porto Velho, Brazil
^3^  Centro de Referencia Saúde da Mulher, Porto Velho, Brazil

**Introduction:** Adolescence is understood by the World Health Organization to be between 10 and 19 years of age, and is linked to the emergence of secondary sexual characteristics, the development of psychosocial processes and the definition of patterns for the differentiation of childhood and adulthood. There are several risk factors that make this group vulnerable, and one of them is the early and in many cases unprotected beginning of sexual life. Globally, adolescents and young adults represent 25% of the sexually active population and are also responsible for almost 50% of all Sexually Transmitted Infections (STIs).

**Objectives:** To analyze behaviors related to the sexual and reproductive health of adolescents in an outpatient clinic in the Western Amazon, and to know the prevalence of the main STIs.

**Methods**: A questionnaire was administered, they underwent a gynecological examination to collect biological material for STIs, which was sent for analysis and rapid tests were performed for syphilis, HIV and viral hepatitis B and C.

**Results:** We observed low use of condoms, relationships with more than one sexual partner, consumption of alcoholic beverages and tobacco. Of the 196 adolescents, between 14 and 19 years old, 163 (83.2%) had G. vaginalis, which is not an STI, but is of great importance in maintaining the health of the vaginal environment, 100 (51.0%) Ureaplasma urealyticum/parvum and 09 (4.6%) Mycoplasma genitalium considered commensal; 45 (23%) Chlamydia trachomatis, 11 (5.6%) Trichomonas vaginalis, 9 (4.6%) Herpes simplex, 06 (3.1%) Neisseria gonorrhoeae and 02 (1%) Syphilis.

**Conclusions:** Adolescents are considered a risk group for STIs, as they start their sexual life early, in an irresponsible and unsafe way, putting their health at risk. This study reports the importance of having updated epidemiological data to compose and assist in protocols and programs to improve the population, so that quality care can be promoted.

**P17. Retrospective Study of Estrogen Therapy in Girls with Primary Ovarian Insufficiency**

**N. Omar ^1^, N. Abdul Ghani ^2^, N. Abu Ishak ^3^, A. Ali ^2^, J. Khong ^4^, S. Loh ^2^, A. Zainuddin ^2^**

^1^  Universiti Teknologi Mara, Sungai Buloh, Malaysia
^2^  Universiti Kebangsaan Malaysia, Cheras, Malaysia
^3^  Universiti Islam Antarabangsa Malaysia, Kuantan, Malaysia
^4^  Pantai Hospital, Bangsar, Malaysia

**Introduction and Aim of Study:** Patients with delayed puberty or primary ovarian insufficiency (POI) of various causes including the genetic disorders like Turner syndrome, complete gonadal dysgenesis and Swyer syndrome, have impaired ovarian function thus they have insufficient endogenous estrogen production to promote breast growth, uterine development and furthermore initiation and normal regulation of menses. The aim of this study is to evaluate the effects of conjugated equine estrogen (CEE) which was traditionally used for pubertal induction, on breast and uterine development and attainment of withdrawal bleeding.

**Methods:** A retrospective study involving female adolescents and women who presented with primary amenorrhea and treated with CEE in PAG unit, Hospital Canselor Tuanku Muhriz in 2015 to 2020. Data collection was made by reviewing the case notes from the medical record. Data entry and analysis was done using the SPSS version 22.

**Results:** 31 patients recruited based on the inclusion criteria. 24 patients (77.4%) had withdrawal bleeding, 6 (19.4%) had no bleeding and changed to a different estrogen therapy and 1 (3.2%) didn’t return for follow up. The mean age of starting treatment was 18 (SD = 7.1). Majority was Turner syndrome (9 cases), followed by isolated hypogonadotropic hypogonadism (4), had chronic disease (4), Mosaic Turner (4), idiopathic primary ovarian insufficiency (2), Swyer syndrome (2) and one case each for Kallman syndrome, Prader Wili syndrome, mixed gonadal dysgenesis, 46XX gonadal dysgenesis, partial androgen insensitivity syndrome and pan hypopituitarism. The mean uterine length during first withdrawal bleeding was 4 (SD = 1.1) cm and width was 2 (SD = 0.6) cm, and majority will have Tanner 3–4 breast. Mean duration of treatment was 26 (SD = 7.2) months and treatment duration significantly associated with uterine length (*p* < 0.05).

**Conclusions:** CEE therapy has positive effect on uterine length and contributed to initiation of menses.

**P18. Contraceptive Practices in Adolescents: Insights from a Five-Year Experience at the Family Planning Center of Democritus University of Thrace, Greece**

**P. Tsikouras, E. Oikonomou, K. Nikolettos, D. Kyriakou, T. Nalmpanti, N. Kritsotaki, S. Kotanidou, V. Spanakis, S. Andreou, A. Mouchterem, K. Chalkia, N. Nikolettos**

Department of Obststeris and Gynecology, Democritus University of Thrace, Alexandroupolis, Greece

**Introduction:** The aim of this study was to gather data and derive meaningful insights regarding the influence of familial, social, and religious factors on the selection of contraceptive methods, with a specific focus on emergency contraception. The study encompassed three distinct adolescent populations: Orthodox Christian women, Muslim women residing and working in Thrace, and Muslim women born in Thrace who had immigrated to Germany several years ago.

**Material and Method:** The study’s data were obtained through anonymous questionnaires distributed at the Family Planning Center of Democritus University of Thrace.

**Results:** Out of a total of 164 women who underwent medical assessments at the outpatient clinics of the Family Planning Center between March 2012 and December 2019, 91 women (55.5%) identified as Christian Orthodox, while 73 (44.5%) identified as Muslim. The age range of women in both groups spanned from 14 to 18 years. The primary sources of contraceptive information for the participants were meetings with family planning professionals (Orthodox Christians: 97.8% vs. Muslims: 97.3%), followed by classmates and friends (Orthodox Christians: 69.2% vs. Muslims: 68.5%) and newspapers and magazines (Orthodox Christians: 65.9% vs. Muslims: 64.4%).

**Discussion:** The outcomes of our investigation underscore the crucial role of Family Planning Centers in aiding individuals to make informed decisions regarding contraceptive options. While we acknowledge the influence of religion and socio-cultural environments, our analysis indicates that the sway of religion appears to be less significant when contrasted with socio-cultural factors.

**P19. Abortion Incidence Among Adolescents: A Decade of Observations from the Family Planning Center at Democritus University of Thrace, Greece**

**P. Tsikouras, E. Oikonomou, K. Nikolettos, D. Kyriakou, T. Nalmpanti, N. Kritsotaki, S. Kotanidou, V. Spanakis, S. Andreou, K. Chalkia, N. Nikolettos**

Department of Obststeris and Gynecology, Democritus University of Thrace, Alexandroupolis, Greece

**Introduction**: During puberty, pivotal gynecological concerns encompass the prevention of Sexually Transmitted Diseases (STDs), addressing issues of abuse, and averting unwanted pregnancies. Adolescent abortions emerge as a substantial health challenge, yielding notable personal repercussions for young girls.

**Methods:** Between June 2010 and June 2020, a total of 150 women who were within 49 days after their Last Menstrual Period (LMP) underwent medical termination of pregnancy using 600 mg of mifepristone (RU 486) (Mifegyn) (Group A1). Another 150 pregnant women, between 49 and 63 days of gestation, were administered 600 mg of mifepristone followed by 800 mcg of misoprostol 48 h later (Group A2). All participants were offered surgical abortion if deemed necessary, and a transvaginal ultrasound was conducted to confirm intrauterine pregnancy before medical administration. Indications for abortion included missed abortion, endometrial demise, spontaneous miscarriage, and induced abortions.

**Results:** According to our research findings, none of the cases required surgical abortion. Common side effects such as nausea, vomiting, pain, and moderate vaginal bleeding were observed, none of which necessitated hospitalization. Bleeding commenced earlier in Group A1 compared to Group A2. Additionally, abortion initiation was approximately 3.5 h earlier in Group A1 compared to Group A2. Similarly, the duration between administration and abortion onset was 50% shorter in Group A1 compared to Group A2.

**Discussion:** The most effective initiatives focus on enhancing adolescents’ awareness and experience to prevent unintended pregnancies. Both the combination of mifepristone and misoprostol and the single administration of mifepristone have proven to be safe and efficient methods for medically terminating pregnancies in the early first trimester.

**P20. Adenomyotic Cyst After Metroplasty for Obstructive Mullerian Anomaly**

**Y. Harel, S. Gurevich, K. Ofir, O. Rabinovitch**

ObGyn Department, Sheba Medical Center, Ramat Gan, Israel

**Introduction:** Uterine adenomyosis is a disorder in which endometrial glands and stroma are present within the myometrium, resulting in hypertrophy of the surrounding myometrium. In this clinical case, we present a patient with a rare adenomyotic cyst mimicking obstructive Müllerian anomaly.

**Methods:** A 27-year-old woman presented with severe progressive lower abdominal pain. Four months before her presentation, she had stopped taking her continued combined oral contraceptive (COC) with the intention to conceive. In her past medical history, at 14 years of age, she had a laparoscopic right hemi-hysterectomy and salpingectomy for an obstructive non-communicating uterus. Since then, she has been treated with COC. Bimanual examination revealed a uterus slightly positioned to the left side of the pelvis and a tender mass palpable on the right side of the pelvis. Ultrasound examination showed a bicornuate-shaped uterus, with a left horn continuous to the cervix with 6.8 mm endometrium, and a right horn filled with hyperechogenic material (Figure 12). MRI showed Unicornuate uterus with a round structure with a diameter of 2.3 cm, containing blood, compatible to an adenomyotic cyst (Figure 13). On Laparoscopy, the area of the right “horn” that protrudes slightly outwards was opened with a harmonic scalpel after vasopressin injection and old blood emerged. The adenomyotic cyst was resected, including its mucosa.

**Conclusions:** This clinical case emphasizes the importance of adenomyotic cysts as a differential diagnosis of Mullerian anomalies. This rare diagnosis could be misinterpreted in ultrasound follow-ups and, therefore, should be kept in mind by the clinicians.

**P21. Review of Adnexal Torsion in Pediatric and Adolescent Population During the Period of 10 Years in University Medical Centre Ljubljana**

**T. Kunič Pirš ^1,2^, M. Jakimovska Stefanovska ^1,2^, A. Štolfa Gruntar ^1^**

^1^  University Medical Centre, Ljubljana, Slovenia
^2^  Faculty of Medicine, University of Ljubljana, Ljubljana, Slovenia

**Introduction and Aims of the Study:** Adnexal torsion occurs when the adnexal organs twist around their vascular pedicle resulting in disrupted blood supply to the ovary, the fallopian tube, or both. The clinical presentation of adnexal torsion is nonspecific and includes pelvic pain, nausea, and vomiting. Ultrasound is the first-line imaging modality to evaluate girls with suspected torsion because it is non-invasive, immediately available and easy to perform, but it has limitations and findings have often been inconsistent. The aim of our study was to make a review of all cases of adnexal torsions operated on pediatric and adolescent population in our department during the period of 10 years.

**Methods:** Retrospective review of all cases of adnexal torsions operated on pediatric and adolescent population in Department of gynecology, University Medical Centre Ljubljana, between 2012 and 2022, was made. Demographic characteristics and operative techniques were reviewed. Data were retrieved from the hospital’s comprehensive computerized electronic medical records.

**Results and Discussion:** The cohort was composed of 43 patients with an operative diagnosis of adnexal mass located in the paraovarian area. Median age was 14 years. We mainly used laparoscopic approach in nearly 80% of cases and performed adnexectomy in only 8.6% of patients. There were no surgical complications and postoperative period was uneventful in all of the patients. Our aim in the future is to use the laparoscopic approach more often even in infants and younger children.

**Conclusions:** Undiagnosed and untreated ovarian torsion commonly results in loss of the ovary and may lead to infertility, necrosis with peritonitis and its sequelae. Timely diagnosis is mandatory to avoid these complications. Urgent laparoscopic adnexal detorsion with ovarian conservation should be offered.


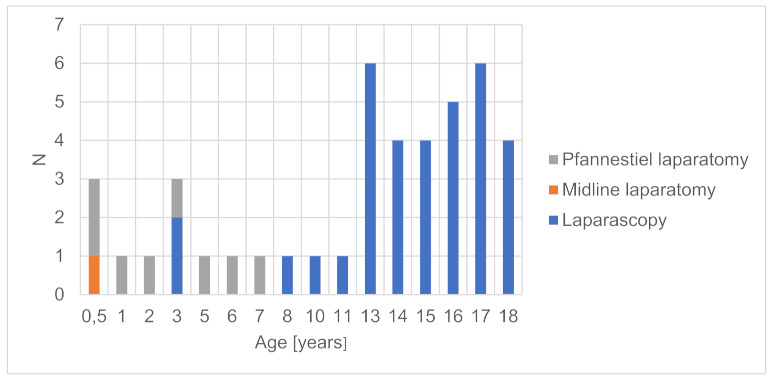

**P22. Delphi Procedure: Diagnostic Criteria of Primary Dysmenorrhea**

**H. Özcan, F. Yarde, J. Albana, J. Huirne, N. Burger, R. De Leeuw**

Department of Obstetrics and Gynaecology, Amsterdam Reproduction and Development Research Institute, Amsterdam UMC, location AMC and VUmc, Amsterdam, Netherlands

**Introduction**: There has been a growing emphasis on research dedicated to understanding and addressing menstrual complaints among adolescents. This is partly due to the fact that menstrual complaints are very common in adolescents. One of the most common menstrual complaints is primary dysmenorrhea. Primary dysmenorrhea often leads to great disruptions in the daily activities of adolescents. Previous studies on primary dysmenorrhea report on different definitions of primary dysmenorrhea. There are studies that describe primary dysmenorrhea as menstrual pain in absence of other pathology. Whereas in daily practice, it is often defined as dysmenorrhea that started with the onset of menarche. Despite much research on primary dysmenorrhea in adolescents, there is no consensus on the meaning of the word ‘primary’ in dysmenorrhea. Neither is there consensus on the diagnostic criteria for the diagnosis of primary dysmenorrhea. In other words, when can we diagnose an adolescent with primary dysmenorrhea?

**Aim:** The objective of this study is to conduct a digital Delphi procedure to reach consensus among international experts, which will result into a standardized definition of primary dysmenorrhea in adolescents.

**Methods:** We performed a digital Delphi procedure in January 2024 to gain consensus on the diagnostic criteria of primary dysmenorrhea in adolescents. The Delphi procedure was performed using digital questionnaires, which were developed after a literature search and evaluation by experts from the Dutch Gynecological Adolescent Care Network. Questionnaires focused on the nomenclature of primary dysmenorrhea, the meaning of the word ‘primary’, whether it’s a diagnosis of exclusion, the necessity to perform pelvic examination, the need for further examination (such as ultrasound), the duration of dysmenorrhea, the severity of dysmenorrhea and the importance of additional symptoms. Consensus on a question was achieved when the Rate of Agreement was 70% or greater.

**Results:** We expect the results and conclusion to follow in April 2024.

**P23. Ovarian Cystadenoma—The Value of Ultrasound in Surgical Treatment and Ovarian Preservation**

**I. Todic ^1^, B. Tomic ^1^, Z. Stankovic ^2^**

^1^  Hospital for Gynecology and Obstetrics”-Clinical Hospital Centre Zemun, Belgrade, Serbia
^2^  President of the Serbian Society of PAG, Belgrade, Serbia

**Introduction and Aims of the Study:** The aim of this study was to the describe the importance of “ovarian crescent sign” (OCS) as a valuable ultrasonographical marker in diagnosis and in choosing the best surgical treatment of ovarian cystadenoma as the one of most common benign neoplasms in pediatric and adolescent patients.

**Methods:** A prospective study using clinical and ultrasound data from patients with ovarian cystadenoma—with and without ovarian torsion, who were surgically treated from January 2006 to December 2023. Study did not include borderline ovarian tumors and ovarian cancer. Age range was 6 to 19 years, with 3 premenarchal girls. Informed consent was obtained from patients and their parents.

**Results:** There was 102 ovarian cystadenoma—58 serous and 44 mucinous. Among them, 9 had ovarian torsion (7 serous and 2 mucinous), with diameter up to 12 cm. Due to tissue edema in those patients OCS was absent. In 7 out of 9 patients with torsion ovary was preserved. From the rest 93 cystadenoma, 35 were huge with median ovarian volume over 500 mL or with diameter over 12 cm and the biggest one had 7.6 kg. The OCS was reported in 30 patients with huge cystadenoma and in all the ovary was preserved, while in the others adnexectomy was performed. Most cystadenomas were smaller with an ovarian volume between 30 to 500 mL or a diameter of 5 to 12 cm, 58 in total. In all 58 patients OCS was reported and the ovary was preserved. The blood level of CA 125 was high in 6 out of 9 patients with ovarian torsion and 16 out of 35 with huge cystadenoma. In the follow-up period formation of new cystadenoma occurred in 5 patients.

**Conclusions:** This study supports the OCS finding on ultrasound as a great predictor for the choice of surgical treatment and further ovarian preservation.

**P24. Choice of Contraceptive Methods in Adolescents in an Ambulatory in the Western Amazon, from 2020 to 2023**

**E. Iglesias Rosa ^1^, M. Ribeiro Simões ^1,2^, S. De Souza Amado ^3^, D. Ferrari ^4^, L. Gonçalves Vieira ^4^, M. Simões Silveira ^2^, G. Rosa de Carvalho ^2^**

^1^  Maternidade Municipal Mãe Esperança, Porto Velho, Brazil
^2^  Centro Universitário São Lucas—AFYA, Porto Velho, Brazil
^3^  Centro de Referência Saúde da Mulher, Porto Velho, Brazil
^4^  Universidade Federal de Rondônia—UNIR, Porto Velho, Brazil

**Introduction:** Adolescence is a psychic-biological process marked by rapid growth and development of the body, mind and social relationships. Physical growth is accompanied by sexual maturity, undergoing intense transformations of the body, stimulated by hormonal action, leading to a series of psychological events that culminate in the acquisition of sexual identity, and the first sexual intercourse, which if unprotected, can lead to pregnancy. unwanted. It is necessary to choose a safe contraceptive method in order to avoid complications in teenage pregnancy, including: hypertensive syndrome of pregnancy, anemia, gestational diabetes, prematurity and complications during childbirth and the postpartum period, thus increasing maternal and infant mortality in this population.

**Objectives:** To analyze which contraceptive method of choice is most prevalent among adolescents treated at an outpatient clinic in the Western Amazon.

**Methods:** Quantitative research was carried out, through a survey of the contraceptive methods chosen by the adolescents in medical records, and descriptive statistics were used, such as: position measurements, central tendency and dispersion.

**Results:** In the period from 2020 to 2023, 2452 adolescents were served with an average age of 18.59 years. Of these 1482 (60.4%) chose copper T IUD 380, 190 (7.7%) quarterly intramuscular injection, 141 (6.4%) monthly combined injection, 125 (5.1%) etonogestrel subdermal implant, 53 (2.2%), combined pill, 34 (1.4%) levonorgestrel IUD, 179 (7.3%) no method and 248 (10.1%) Missing.

**Conclusions:** Long-term methods are superior in terms of safety and effectiveness as they do not depend on daily administration by the adolescent. Contraceptive guidance and appropriate use in adolescence are extremely important for physical, sexual and mental health. Bearing in mind that the destruction or inappropriate use of contraceptive methods can lead to unplanned pregnancy in adolescence. It is essential to evaluate the adolescent individually, explain the risks and benefits of each contraceptive method, expand the supply of safe methods, and thus reduce unplanned pregnancies.

**P25. Primary Amenorrhea: Turner Syndrome with Incidental Pituitary Microadenoma**

**J. Soriano, R. Eustaquio-David**

Davao Doctors Hospital, Davao City, Philippines

Turner syndrome is a genetic disorder in females caused by the partial or complete absence of one of the X chromosomes. The condition affects approximately 1 in every 2500 female livebirths. Patients with Turner Syndrome have hypergonadotropic hypogonadism and they can present with short stature and other medical conditions, such as endocrine disorders, autoimmune disease, and cardiovascular disease. Pituitary adenomas have rarely been identified in patients with Turner Syndrome and only a few cases have been reported in literature.

A 16-year-old female with developmental delay and primary amenorrhea was diagnosed with Turner syndrome, based on karyotype 45, X. Physical examination revealed a patient with short stature. She had webbed neck, a broad chest, and widely spaced nipples, cubitus valgus with Tanner Stage 1 breast and Tanner Stage 3 pubic hair. Transrectal sonography revealed an infantile uterus and gonads. Endocrinologic investigation revealed hormone level deficiencies manifesting as hypergonadotropic hypogonadism with hypoestrogenism and high levels of follicular stimulating hormones and luteinizing hormones. Cranial Magnetic Resonance Imaging (MRI) of the pituitary gland revealed pituitary microadenoma measuring 4.9 × 3.8 mm.

There are two conditions coexisting in this case of primary amenorrhea. Thus necessitates a comprehensive, multidisciplinary approach to management. The patient’s prolactin level was normal, suggesting the microadenoma is non-functional. Initiation of puberty was planned with hormone replacement and close monitoring of the pituitary microadenoma was advised.


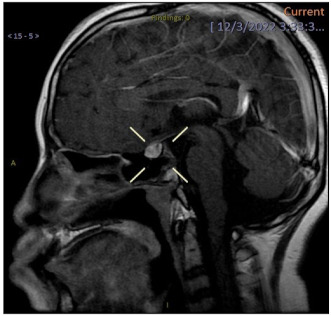

**P26. Case Series: Lipschütz Ulcer in the Gynecologyc Emergency Department of Hospital La Fe**

**V. Monterrey Perera, A. Carrasco Catena, D. Desantes Real, F. Valero Domínguez, B. Marcos Puig**

La Fe University and Polytechnic Hospital, Valencia, Spain

**Introduction:** Lipschütz ulcer is a condition characterized by the onset of genital ulcers in young women. Its cause remains unclear. Our aim is to review its characteristics in our environment to clarify its management and possible associations (mostly EBV, CMV).

**Material and Methods:** Data have been collected retrospectively in the last 10 years in the Emergencies of La Fe University Hospital (Valencia, Spain). A total of 34 patients consulted with similar symptoms, collecting data such as age, clinical presentation, ulcer characteristics, treatment and evolution.

**Results:** Of the 34 patients, 21 were under 25 years old (61%). Except 2 patients, an upper respiratory tract condition was detected in the previous 7 days. Ulcers had similar characteristics (fibrin/necrotic base, 2 cm, erythematous edges, located on the inner surface of labia minora, except 3 cases where occurred on labia majora). In 12 cases the ulcer was mirror-shaped (57%). Ulcer’s PCR was negative in all cases in which a sample was collected (13 cases) and 3 presented superinfection with germs from intestinal flora. Serologies were negative except for 4 cases positive for EBV, 1 for CMV and 1 for HSV. Only on one occasion was biopsied revealing necrotic tissue with microabscessification and abundant cellular debris. Treatment was symptomatic with ointments, NSAIDs and in some cases topical corticosteroids or antibiotics. Symptoms resolved within 6–9 days, except 2 of them which resolved after 3 weeks and one of them recurred with oral ulcers, suspecting Behçet’s disease.

**Conclusions:** It is an acute, benign condition with clinical diagnosis. In our experience, not resolved cases usually hide other diagnosis. Serologies were mostly negative. It should be noted that although cases mainly occur in people aging under 25, in our environment, 61% occurred in this age group.

**P27. Clinical Presentation, Evolution and Management of Peripheral Precocious Puberty Due to Autonomous Ovarian Cysts: A Case Series**

**M. Marinescu ^1^, C. Procopiuc ^1^, A. Caragheorgheopol ^1,2^, C. Dumitrescu, I. Gherlan ^1,2^**

^1^  National Institute of Endocrinology C.i. Parhon, Bucharest, Romania
^2^  University of Medicine and Pharmacy “Carol Davila”, Bucharest, Romania

**Introduction and Aim of the Study:** Autonomous ovarian cysts, representing 5% of ovarian cysts seen in prepubertal age, are the most common cause of peripheral precocious puberty (PPP). They can be isolated or syndromic, with most cysts regressing spontaneously. Some can be recurrent and lead to progression to central precocious puberty and the therapeutic options are inconstantly proved to be efficient. The aim of the study is to present three different clinical courses and management of PPP cases due to autonomous ovarian cysts.

**Results:** At diagnosis, they were 4.2, 1.3 years and 10 months old. Clinical manifestations consisted of thelarche B3, leucorrhea and vaginal bleeding. Upon examination, none of them had café-au-lait spots. FSH and LH were undetectable, and E2 was 129, 324 and 212 pg/mL, respectively. All patients had suppressible GH values in OGTT and normal thyroid function. Pelvic ultrasound revealed ovarian asymmetry with unilateral ovarian cysts of 33/21, 25/12 and 26/22 mm. The first patient was successfully treated with 10 mg medroxyprogesterone acetate (MPA) and, on a follow-up period of 4 years, presented one recurrence; the second patient, on a 9-year follow-up, had multiple recurrences requiring progressive doses of MPA from 5 mg to 20 mg and lastly association with an aromatase inhibitor. The third patient was given Letrozole and Anastrozole and, during the 4.9 years of follow-up, had 4 recurrences; later in evolution developed café-au-lait spots and fibrous dysplasia lesions and McCune-Albright syndrome was diagnosed.

**Conclusions:** In all patients, pubertal clinical manifestations were notable. The two girls under 3 years old at diagnosis had a significant evolution, one with recurrent isolated functional ovarian cysts and the other with a later diagnosis of McCune-Albright Syndrome. None of the patients developed secondary central precocious puberty, neither ovarian torsion and their pubertal progression was well controlled with pharmacological treatment.

**P28. Giant Juvenile Fibroadenoma of the Breast: A Clinical Case**

**M. López Sanclemente, M. Sánchez Prieto, E. Coello Cahuao, R. Sánchez Borrego**

Diatros Clinic, Barcelona, Spain

**Introduction:** Juvenile fibroadenoma is a common cause of breast masses seen in adolescents. It defines like a benign focal tumor usually unilateral, containing both glandular and mesenchymal elements. They are referred to as “giant fibroadenoma” when larger than 5 cm. Pathogenesis includes excessive stimulation of estrogen and progesterone or increased receptor sensitivity.

**Methods:** We aimed to report a “juvenile giant fibroadenoma” case admitted to Diatros Barcelona Clinic in September 2021. We collected the data from our clinical records.

**Results:** We present a clinic case about a 13-year-old female that was evaluated due to a painful mass that was growing on her right breast during the previous 6 months and that made her right breast bigger than the left breast (Figure 14). Her past medical and family histories were uneventful. At the physical examination she had an important breast asymmetry with a mild painful subareolar 5 cm mass on her right breast. The left breast was smaller than the right and both axillae were normal. The right breast ultrasound showed a solid lesion of 56 × 55 × 29 mm with well-defined borders, negative flow doppler, sonographically compatible with a breast fibroadenoma. We excised the 5 cm mass under general anesthesia with an areola border incision, trying to preserve the anatomy and esthetics of the breast. Histopathological examination of the patient was reported as a benign giant fibroadenoma and she recovered without any problems in the postoperative period. The esthetic result was excellent, preserving a good shape, minimum scar and improving the breast symmetry.

**Conclusions:** Giant juvenile fibroadenoma (Figure 15) is a fast-growing mass that can appears during the breast development stage and must be biopsied and excised to differentiate from other conditions like phyllodes tumor.

**P29. Trans-Abdominal Ultrasound in the Evaluation of Dysmenorrhea in Adolescents**

**M. Rosenberg Friedman ^1,2^, M. Benish ^1,3^, L. Reicher ^1,2,4^, A. Segev-Becker ^1,5^, S. Bar-On ^1,2^, I. Levin ^1,2^, C. Klieger ^1,2^**

^1^  Faculty of Medicine, Tel Aviv University, Israel
^2^  Lis Maternity Hospital, Tel Aviv Sourasky Medical Center, Tel Aviv, Israel
^3^  Pediatric Hemato-oncology department, Dana Children’s hospital, Tel Aviv Sourasky Medical Center, Tel Aviv, Israel
^4^  Department of Computer Science and Applied Mathematics, Weizmann Institute of Science, Rehovot, Israel
^5^  Pediatric Endocrinology Unit, Dana Children’s hospital, Tel Aviv Sourasky Medical Center, Tel Aviv, Israel

**Introduction:** Dysmenorrhea profoundly affects the quality of life of adolescents following menarche and many seek medical assistance for symptomatic alleviation. Endometriosis is a known cause for chronic pelvic pain and dysmenorrhea in this age group.

**Aims of Study**: To assess the effectiveness of transabdominal pelvic ultrasound (TAUS) as a diagnostic tool for dysmenorrhea in adolescents.

**Methods**: Data were extracted from our institutional computerized database of adolescents with dysmenorrhea treated at Tel Aviv Sourasky Medical Center during 2021–2023. The study was approved by the Institutional Review Boards.

**Results:** Overall, 219 girls addressed the adolescent clinic, 46 (21%) reported dysmenorrhea, of them 30/46 (65%) completed TAUS as part of initial assessment. Nineteen patients that completed TAUS (63%) did not show any pelvic sonographic abnormalities, therefore regarded as primary dysmenorrhea. The remaining 11/30 patients (37%) showed pathological findings on TAUS, but none related to endometriosis. Six out of the 11 patients (55%) demonstrated polycystic ovaries, and the remaining 5/11 (45%) displayed other signs of secondary dysmenorrhea, including ovarian cysts (3), adenomyosis (1), and congenital Müllerian malformations (1). Of note, 3/30 (10%) underwent expert-guided endometriosis-dedicated TAUS, that did not reveal any pathology. All patients with dysmenorrhea were advised regarding oral contraceptives (OCP) and/or non-steroidal anti-inflammatory drugs (NSAIDS). However, only 14/46 (30%) returned for follow-up: 8/14 (57%) used NSAIDS only, 3/14 (21.5%) OCP only and 3/14 (21.5%) used combined treatment. 11 (79%) patients improved under treatment and the rest were referred for further evaluation.

**Conclusions:** According to our findings, TAUS is not an effective diagnostic tool for endometriosis in adolescents, and treatment decisions should be based on clinical symptoms. However, we highly recommend performing TAUS as an initial evaluation of dysmenorrhea to exclude other causes that should be treated differently.

**P30. Paratubal Cysts in Obese Adolescents: Insights from a Case Series Review**

**N. Mohd Sakri, N. Azmirrulnizam, N. Zainon, N. Abu Ishak**

Paediatric Adolescent Gynaecology Unit, Department of Obstetrics and Gynaecology, Kulliyyah of Medicine, International Islamic University Malaysia (IIUM), Kuantan, Malaysia

**Introduction:** Paratubal cysts (PTCs) are adnexal masses originating from the mesosalpinx and constitute approximately 10% of such masses. They are typically benign. The incidence of PTCs has been found in 7.3% of paediatric and adolescent populations. There is an association between PTCs, obesity, and hyperandrogenism.

**Methods:** The cases of PTCs in obese adolescents were identified, and the information was retrieved from the electronic medical record.

**Case Descriptions:** The girls ranged from 10 to 15 years old, with confirmed PTCs. These adolescents were obese, with BMIs ranging from 35 to 47 kg/m^2^, putting them at risk of developing polycystic ovarian syndrome (PCOS). *Case 1*: A 15-year-old was incidentally found to have a huge right PTC, reporting amenorrhea and rapid weight gain. *Case 2:* A 10-year-old prepubertal girl presented with intermittent right lower abdominal pain associated with gastrointestinal symptoms suspicious of a mesentery cyst, revealing a twisted right PTC intraoperatively. *Case 3:* A 15-year-old presented with an acute abdomen and oligomenorrhea. Intraoperatively revealed a right multiloculated PTC. *Case 4:* A 15-year-old experiencing severe abdominal pain and irregular menses underwent emergency laparoscopic detorsion of a left PTC.

**Discussion:** The occurrence of PTCs in obese adolescents is significantly related to hyperandrogenism and warrants careful examination. Three of the patients were postmenarcheal. Considering that menarche indicates hypothalamic-pituitary-gonadal axis functionality, hormonal mediation of PTCs is plausible. Recent research suggests that PTCs are associated with PCOS-like symptoms such as hirsutism, hyperandrogenism, obesity, and menstrual irregularity, and that their growth is promoted by excess androgens due to their origin in the Wolffian duct remnants.

**Conclusions:** Recognizing and managing PTCs in obese adolescents is crucial to mitigate potential long-term hormonal dysregulation and PCOS sequelae. Early intervention through comprehensive assessment for signs of PCOS, including early adrenarche, menstrual irregularities, and hyperandrogenism, is essential to prevent further complications.

**P31. NEO-Vaginal Creation in Adolescents and Young Women: An Alternative Approach**

**N. Abu Ishak ^1^, A. Ali ^2^, N. Omar ^3^, N. Abdul Ghani ^2^, A. Zainuddin ^2^**

^1^  International Islamic University Malaysia, Kuantan, Malaysia
^2^  Universiti Kebangsaan Malaysia Medical Centre, Kuala Lumpur, Malaysia
^3^  Universiti Teknologi MARA, Puncak Alam, Malaysia

**Introduction:** Vaginal dilatation therapy is a non-invasive therapy advocated for females with pathologies presenting with inadequate or non-existent vagina. Hereby, we share our centre’s experience in intensive vaginal dilatation therapy among adolescents and young female adults.

**Methods**: All patients indicated for intensive vaginal dilatation from year 2019 to 2021 in UKMMC. Dilators of various sizes were used, and adjuvant treatment were utilised consisting of oral Paracetamol and topical application of estrogen and Lignocaine gel.

**Results:** There were total of 9 cases; 5 were Malays, 3 Chinese and 1 Indian. The cases were 3 of vaginal dysgenesis, 3 cases of MRKH, 2 vaginal stenosis/atresia and one transverse vaginal septum. Ages range from 14 to 30 years old. All of them have had prior trial of self vaginal dilation at home which failed. They were admitted for 3 to 5 days duration. Upon commencement of therapy, the mean vaginal length was 3.5 cm with mean width of 1.8 cm. Each session of intensive vaginal dilatation lasted for 30–60 min; 2–3 times per day. Patients were able to independently perform self vaginal dilatation under direct supervision by the third day. Each patient was able to successfully attain a vaginal length of 4–8 cm with overall mean vaginal length prior to discharge of 5.5 cm and width of 2.2 cm. Initial pain score was 6–8 over 10, which was markedly reduced with aide of adjuvant therapy to 0–2 over 10.

**Conclusions:** Non-invasive increment of vaginal length is achievable via intensive vaginal dilatation therapy. This is a promising approach even among Asian adolescents and young adults with various religious and cultural limitations. A dedicated team coupled with clear, directed and patient-centred protocol with usage of adjuvant therapy were all found to be contributing to successful outcomes.

**P32. Pregnancy Outcome in Adolescent Women in Greece**

**A. Papailiou, A. Psarris, L. Michala, P. Antsaklis, M. Theodora, G. Daskalakis**

1st Department of Obstetrics & Gynaecology, Alexandra Hospital, University of Athens, Athens, Greece

**Background:** Teenage pregnancy is a global phenomenon with serious health, social and economic consequences. Its prevalence is greater in societies with low socioeconomic status. Child marriage, sexual abuse and the lack of access to contraceptive methods constitute the main factors contributing to it. Pregnancies at an early age lead to an increased incidence of low birth weight, preterm birth, perinatal mortality, preeclampsia, systemic infections and feelings of social isolation and postpartum depression. This study aims to look at characteristics of adolescent pregnancies in a tertiary maternity hospital in Greece.

**Methods:** We performed a retrospective record review, to identify pregnant women under the age of 18 years, delivering in our institution between January 2018 and December 2023. We collected data on demographic characteristics of the mother, gravidity, parity, gestational age, mode of delivery, indication of c-section, pregnancy and birth complications, Apgar score, neonatal gender, weight and type of anesthesia.

**Results:** In the last 5 years, 592 adolescent women gave birth in our hospital, out of 17.088 deliveries in total. The mean age was 15.9 years (range 12.5–17). The annual average of deliveries among teenagers was 120. In 2023, 148 teenager women gave birth, the highest incidence in the last five years, despite a decrease in the total number of deliveries. The mean gestational age at delivery was 35 + 2 weeks. About two out of three teenager women gave birth vaginally, while in general population the vaginal delivery rate is 50 percent.

**Conclusions:** Literature on teenage pregnancies is scarce and mostly comes from developing countries. This study is important as it presents evidence from an EU country and confirms the increased incidence of late preterm delivery and vaginal birth.

**P33. Uterine Leiomyomas in Adolescence**

**V. Bais, A. Vatopoulou, F. Gkrozou, X. Skentou, A. Sosoni, M. Pasxopoulos**

University Hospital of Ioannina, Ioannina, Greece

**Introduction and Aim of the Study:** Uterine leiomyomas (UL) or fibroids are benign smooth muscle tumors originating from the uteral wall. Although the incidence of UL at the stage of menopause is high, calculated at approximately 70% and 20–30% of women between the ages of 35 to 50, their occurrence in adolescence is extremely rare (<0.5%). The aim of the study was to research rare cases of UL in adolescent girls, determine the symptoms they presented with and the treatment they were administrated.

**Methods:** We searched Pubmed for published cases of UL occurring in adolescence from 2010 until 2024.

**Results:** Our search revealed 15 cases of UL in adolescent girls after 2010. We reviewed the main symptom the cases presented with, the treatment they were given, the dimensions of the masses and the age of the patients. As for the main symptom, six (6) presented with a pelvic mass or abdominal distention, two (2) with abdominal pain, six (6) with abnormal uterine bleeding and one (1) with dyspareunia. The treatment of choice in all of the cases was myomectomy. The majority were performed abdominally (6/15), followed by laparoscopic myomectomy (4/15). Three (3) were performed hysteroscopically, one (1) vaginally and one (1) case did not specify the treatment that was used. The youngest patient that presented with a UL was twelve years old and the bigger mass was 25 × 15 × 10 cm.

**Conclusions:** Uterine leiomyomas despite being extremely rare in adolescence they should should not be excluded from the differential diagnosis of a pelvic mass in younger ages. The treatment of choice is myomectomy, most often performed abdominally than laparoscopically because laparoscopy it is technically more difficult in adolescence, with a great emphasis on fertility preservation.

**P34. Insights from the Hematology-Adolescent Medicine Clinic**

**M. Benish ^1,2^, M. Rosenberg Friedman ^1,3^, A. Segev-Becker ^1,4^, L. Reicher ^1,3,5^, R. Elhasid ^1,2^, S. Bar-On ^1,3^, C. Klieger ^1,3^**

^1^  Faculty of Medicine, Tel Aviv University, Tel Aviv, Israel
^2^  Pediatric Hemato-oncology Department, Dana Children’s hospital, Tel Aviv Sourasky Medical Center, Tel Aviv, Israel
^3^  Lis Maternity Hospital, Tel Aviv Sourasky Medical Center, Tel Aviv, Israel
^4^  Pediatric Endocrinology Unit, Dana Children’s hospital, Tel Aviv Sourasky Medical Center, Tel Aviv, Israel
^5^  Department of Computer Science and Applied Mathematics, Weizmann Institute of Science, Rehovot, Israel

**Introduction**: Menorrhagia frequently drives adolescents to seek medical attention. The hematological assessment for potential bleeding disorders typically involves a comprehensive coagulation workup, including prothrombin time (PT), partial thromboplastin time (PTT), assessment of von Willebrand factor (VWF) antigen and activity levels, and evaluation of platelet function.

**Aims**: The primary aim of our study was to evaluate the efficacy of personal or familial bleeding history as a screening tool for underlying bleeding disorders in adolescents presenting with menorrhagia. Additionally, we investigated the prevalence of menorrhagia as a standalone complaint versus its coexistence with dysmenorrhea or hormonal imbalances such as polycystic ovary syndrome (PCOS).

**Methods**: We searched our institutional computerized database to extract records of adolescents with menorrhagia who visited the Adolescent clinic in Tel Aviv Sourasky Medical Center during 2021–2023. The study was approved by the Institutional Review Boards.

**Results**: Out of the sixty adolescents who presented to the adolescent clinic with menorrhagia, 42 were specifically queried regarding personal or familial bleeding tendencies. Among them, we identified such tendencies in 20/42 (48%) of patients. However, only 9/20 of them (45%) underwent a comprehensive coagulation workup. Remarkably, of those nine, five (55%) exhibited abnormal platelet function, while none showed abnormalities in PT, PTT, or VWF antigen or activity levels. Furthermore, 19/60 patients (32%) reported significant dysmenorrhea, while eight (13%) met the Rotterdam criteria for PCOS.

**Conclusions:** The majority of patients with personal of familial bleeding history, who completed the coagulation workup, exhibited platelet function disorders. However, our study suggests that this criterion alone may not possess adequate sensitivity as a screening tool. Our findings underscore the importance of a systematic evaluation for bleeding disorders, particularly emphasizing the inclusion of assessments related to hyperandrogenism and dysmenorrhea. Combined estrogen-progestin oral contraceptive pills should be considered as an alternative therapy for adolescents with menorrhagia and PCOS.

**P35. The Problem of Teenage Pregnancy in Europe over the Last Decade**

**N. Koutalia, A. Vatopoulou, C. Skentou, F. Gkrozou, E. Moulias, M. Paschopoulos**

University Hospital of Ioannina, Department of Obstetrics and Gynaecology, Ioannina, Greece

**Introduction and Aims of the Study:** Adolescent pregnancy is defined as the occurrence of pregnancy in girls aged 10–19. It is a public health issue with physical, psychological, and socioeconomic consequences. Globally, approximately 13% of adolescents give birth before age 18. The aim of this study is to present the trends of teenage pregnancies and abortions in Europe over the last decade.

**Methods:** We performed a search of all published studies in PubMed and other databases (WHO, UNICEF, EUROSTAT) from 2013 to 2024.

**Results:** According to WHO, Bulgaria (37.9/1000 teenagers) and Romania (35/1000) have the highest rates of teenage pregnancies in 2024 whereas Denmark, Switzerland and Norway have the lowest rates. UNICEF’s data from 2015–2021 demonstrate that Bulgaria and Romania also had the highest incidence, followed by Slovakia and Hungary. The lowest rates were recorded in Norway, Switzerland and Denmark. According to Eurostat, 1 in 8 first children were born to mothers aged below 20 in Bulgaria and Romania in 2017. The lowest shares of births were observed in Denmark (1.0%), Italy and Slovenia (1.1%) and the Netherlands (1.2%). The abortion rates have shown a decline but they vary widely, as the European abortion policies are still very heterogenous. The greatest decline is noted in Estonia and Slovenia. A study, conducted in Greece between 2018–2019, reported that 9.7% of women had at least one abortion at 15–19 years. This proportion was lower compared to France (15.2%) but higher compared to Germany (5.9%).

**Conclusions:** A decline in teenage pregnancy rates is observed in Europe. Romania and Bulgaria recorded the highest rates over the last decade. Considerable variation in abortion rates is noted as legal abortion is not accessible for teenagers in all European countries.

**P36. A Study of a Paediatric and Adolescent Gynaecology Service in a Tertiary Level Teaching Hospital in Hong Kong**

**N. Lee, P. Ip, K. Lee, S. Chan, K. Ng**

The Chinese University of Hong Kong, Hong Kong

**Introduction and Aims:** The Paediatric and Adolescent Gynaecology (PAG) service at The Prince of Wales Hospital a regional referral centre in Hong Kong. We aim to review the service and numbers of new referrals, in order to better understand the scope and uptake of PAG services.

**Methods:** A retrospective review of the PAG service over 3 years. A total of 1445 patients were included in the study. We identified patients attending the PAG clinic for the first time between 1 January 2021 and 31 December 2024. Data was collected from a proforma that was completed after each visit and entered into SPSS for analysis.

**Main Outcome Measures:** Number of new referrals, referral source, body mass index (BMI), age, and presence of co-existing mental health or learning difficulties.

**Results:** There were 384, 459 and 602 new referrals seen in 2021, 2022 and 2023 respectively. Referral source data was available in 143 (37%), 184 (40%) and 189 (31%) of cases from 2021–2023, and 516 (35%) overall. The most common referral source was from paediatrics (47.7%), followed by surgery (10.3%), another in-hospital speciality (excluding paediatrics, surgery and psychiatry) (9.5%), the emergency department (8.5%), private doctors (7.6%), self-referral (2.9%) and psychiatry (2.5%). Patients’ median age was 13 (IQR, 15, 17). Median BMI was 21 (IQR, 17.8, 24.9), 20.3 (IQR, 17.7, 24.5), and 19.9 (IQR, 17.9, 25); 31.7%, 26.4%, and 21.1% had a co-existing mental health diagnosis or learning difficulty in 2021–2023 respectively.

**Conclusions:** The number of referrals has gradually increased over the 3 year period included in this study. This demonstrates an increasing demand and utilisation of PAG services, in keeping with international guidelines that recommend that children should be seen by gynaecologists with specialised PAG training. A significant proportion of new referrals had a co-existing mental health diagnosis or learning difficulty.

**P37. Hematological Profile of Pregnant Adolescents. A Decade Survey in Northern Greece**

**S. Theodoridou ^1^, A. Vyzantiadis ^1^, T. Theodoridis ^2^, A. Sotiriadis ^3^, A. Mamopoulos ^4^, K. Dinas ^3^, A. Athanasiadis ^2^**

^1^  Hippokration Hospital, Thalassemia Prevention Unit, Blood Bank Center, Thessaloniki, Greece
^2^  AUTH 1st Gynecology Department, Thessaloniki, Greece
^3^  AUTH 2nd Gynecology Department, Thessaloniki, Greece
^4^  AUTH 3d Gynecology Department, Thessaloniki, Greece

**Background:** Births from adolescent mothers account for 10% of all births in the world and for 23% of maternal morbidity and mortality. Adolescence is a period of biological modifications and teens have a risk of developing iron deficiency anemia. The objective of this research was to determine the “hematological” profile and compare the distribution as well as the severity of anemia, among the pregnant adolescents (12–19-year-old), during a ten-year period in Northern Greece.

**Material and Methods**: A retrospective study of the medical records of pregnant adolescents were made on the occasion of their first prenatal consulting. The laboratory workout was undertaken in the Thalassaemia Prevention Unit of the Hippokration Hospital of Thessaloniki, Greece, from 2013 to 2022.

**Results:** 209 of the adolescents were of Greek Nationality (88.3%) (Greek natives of the general population N = 86, 41.1%, members of the Roma community N = 112, 53.5%, members of the Muslin minority of Thrace N = 11, 5.2%) and 29 (11.6%) were immigrants from the Balkans, Middle East and Asia. The mean ± SD age of the 238 pregnants was 16.67 ± 1.67 years and most of them (90%) were between 15–19 years approximately and 10% of them were between 12–14 years. Roma were the most (47%) and youngest (15.9 ± 1.3 years old) adolescent pregnant s. All cases had abandoned school. From our data, anemia was found in 33.6%, iron deficiency anemia in 28.5% and iron deficiency in 54.6%. Migrants and Roma had the lowest hemoglobin levels in the second and third trimester of pregnancy respectively, while the lowest ferritin levels were found in Roma and Muslims of Thrace, though with not statistically significant difference among the groups.

**Conclusions:** Despite the knowledge about the prevalence of adolescent pregnancy and its adverse effects, it still remains a socio-medical problem and actions are crucial to support pregnant s and families.

**P38. Evaluating Current Practices and Barriers Towards the Provision of Fertility Preservation Services for Children and Adolescents in a Developing Country**

**A. Anizah ^1^, N. Omar ^2^, N. Abu Ishak ^3^, N. Abdul Ghani ^1^, L. Yew Kong ^4^, H. Alias ^5^, A. Zainuddin ^1^**

^1^  PAG Unit, Department of Obstetrics and Gynaecology, Hospital Canselor Tuanku Mukhriz, Faculty of Medicine, Universiti Kebangsaan Malaysia (UKM), Cheras, Malaysia
^2^  Department of Obstetrics and Gynaecology, Faculty of Medicine Universiti Teknologi MARA (UiTM), Sungai Buloh, Malaysia
^3^  Department of Obstetrics and Gynaecology, Faculty of Medicine, International Islamic University of Malaya (IIUM), Kuantan, Malaysia
^4^  Department of Primary Care Medicine, Faculty of Medicine, Universiti Malaya (UM), Petaling Jaya, Malaysia
^5^  Department of Paediatric, Hospital Tunku Ampuan Besar Tuanku Aishah Rohani, Faculty of Medicine, Universiti Kebangsaan Malaysia (UKM), Cheras, Malaysia

**Introduction:** Fertility preservation (FP) has emerged as solution to combat fertility impairment risk in childhood cancer patients undergoing cancer treatment. FP services catering to children and adolescents (C&A) in developing countries are limited. Malaysia recently launched its FP services for C&A.

**Aims of Study:** To evaluate healthcare providers (HCPs) FP practices and identify barriers towards FP C/S for C&A.

**Methods:** A questionnaire-based study was conducted to evaluate the current FP practices for C&A amongst HCP, and identify barriers towards FP C/S provision. A questionnaire consisting of 4 questions was adapted from G. Quinn et al. (2009). The questionnaire was distributed both online and physically amongst HCPs in a tertiary center. Ethical committee approval was granted by the Research Ethical Committee, Universiti Kebangsaan Malaysia.

**Results:** A total of 102 HCPs completed the questionnaires. Majority of respondents were Malays (74.5%), females (80.4%), gynaecology/pediatrics specialty (80.4%), and had children (88.2%). Nearly 73% HCPs consulted reproductive specialists (RES) on potential fertility issues. Furthermore, 83% HCPs referred patients who enquired on fertility issues to RES. Only 17% HCPs practiced FP discussion, 10% HCPs were unaware of who to discuss FP with, and 12% reported no available person to discuss FP. Top three barriers to FP C/S were patients could not afford FP service (30.4%), limited available information on FP for patients (17.6%), and patients too ill to delay treatment (12.7%). Discussions: HCPs in our study reflected good current FP practice behaviors, and highlighted the main barriers to the uptake of FP C/S for C&A being patient and resource barriers. Mitigating these issues via FP knowledge dissemination, FP funding assistance, and developing pediatric FP-related educational materials would translate to an improved FP service provision for C&A.

**Conclusions:** In summary, strategic planning coupled with effective remedial measures promises a bright path ahead for FP services provision for C&A in Malaysia.

**P39. MR Imaging Algorithm of Amenorrhea in Young Females**

**M. Konidari ^1^, P. Christopoulos ^2^, N. Vlahos ^2^, L. Moulopoulos ^1^, C. Bourgioti ^1^**

^1^  1st Department of Radiology, National and Kapodistrian University of Athens, Areteion Hospital, Athens, Greece,
^2^  2nd Department of Obstetrics & Gynecology, National and Kapodistrian University of Athens, Areteion Hospital, Athens, Greece

**Introduction/Aims:** Normal menstruation depends on the complex coordinated function of the hypothalamic–pituitary axis, the physiologic hormonal function of the adrenal glands and gonads and the normal anatomy of female internal genitalia and outflow tract.

Amenorrhea is defined as the absence or abnormal cessation of menstrual bleeding. Primary amenorrhea is the absence of menarche by 16 or 14 years of age with normal or absent growth and development of secondary sexual characteristics, respectively (Figure 16). Secondary amenorrhea is the absence of menstruation for at least 6 months, in a female with a previously normal cycle.

Amenorrhea may be caused by a variety of pathologic conditions that affect any of the following three levels: (I) uterine/vaginal outlet, (II) ovarian/gonadal and (III) hypothalamic-pituitary level. Depending on the suspected cause, imaging of the pelvis, abdomen or brain can help in establishing an accurate diagnosis. MRI is the imaging modality of choice for detailed evaluation of internal genitalia.

The objectives are to:

- Provide an MRI—based imaging approach in the evaluation of amenorrhea in young females.
- Understand the most common causes of amenorrhea and familiarize with MR imaging findings through a case-based discussion.

**Methods:** MR imaging findings of various conditions associated with amenorrhea are illustrated through a case-based discussion.

**Results and Discussion:** In adolescents presenting with amenorrhea, detailed assessment of the pelvis, and in some cases the upper abdomen or the brain with MRI, especially when pelvic ultrasound findings are inconclusive, is essential in order to provide an accurate diagnosis. Specific appearances of the uterus and gonads are associated with different causes of the condition and should be carefully assessed along with clinical and hormonal findings in order to provide tailored clinical decisions.

**Conclusions:** Along with clinical findings and hormonal evaluation, MR imaging can provide useful information and be a problem-solving tool in the diagnosis and management of amenorrhea.

**P40. MRI of Müllerian Duct Anomalies Based on the New ASRM Classification**

**M. Konidari ^1^, P. Christopoulos ^2^, N. Vlahos ^2^, L. Moulopoulos ^1^, C. Bourgioti ^1^**

^1^  1st Department of Radiology, National and Kapodistrian University of Athens, Areteion Hospital, Athens, Greece
^2^  2nd Department of Obstetrics & Gynecology, National and Kapodistrian University of Athens, Areteion Hospital, Athens, Greece

**Introduction/Aims:** Müllerian duct anomalies (MDAs) consist of a wide spectrum of congenital malformations of the uterus, cervix, fallopian tubes and upper 2/3 of the vagina and are the result of abnormal development, fusion or resorption of the Müllerian ducts. The new 2021 American Society of Reproductive Medicine-ASRM (previously, American Fertility Society-AFS) MDAs classification (MAC2021) expands the 1988 classification to include cervical and vaginal anomalies; it classifies MDAs into nine categories using standardized descriptive terminology.

The objectives are to:

- Illustrate the MRI appearances of congenital uterine tract anomalies according to the new ASRM classification.
- Provide a diagnostic algorithm for imaging approach of MDAs.

**Methods:** Case-based discussion on MRI appearances, clinical presentation and treatment management of MDAs, according to the new ASRM classification.

**Results/Discussion:** MRI provides better soft tissue resolution among different pelvic structures and larger field of view compared to ultrasound (Figure 17). Therefore, it is the modality of choice for detailed pelvic anatomy evaluation, accurate MDA classification and preoperative planning. Clinicians should be familiar with MR appearances of congenital uterovaginal anomalies in order to better plan clinical management.

**Conclusions:** Imaging is crucial in the diagnostic workup of individuals with suspected MDAs, particularly adolescents with primary amenorrhea, since identification and sufficient description of these anomalies and their complications allows tailored clinical decisions.

**P41. Multicystic Dysplastic Kidney and Involution: A Prompt for Early Detection of Obstructed Hemivagina with Ipsilateral Renal Agenesis (OHVIRA)**

**A. Rouvali ^1^, L. Michala ^1^, G. Papaioannou ^2^**

^1^  Paediatric and Adolescent Gynaecology 1st Department of Obstetrics and Gynaecology Alexandra Hospital, Athens, Greece
^2^  Department of Pediatric Radiology Mitera Maternity and Children’s Hospital, Athens, Greece

**Introduction**: Obstructed Hemivagina with Ipsilateral Renal Agenesis (OHVIRA-Syndrome) is a rare Mullerian malformation, associated with mesonephric abnormalities and is usually diagnosed after menarche due to symptoms related to haematocolpos. Early and accurate diagnosis and treatment are of the utmost importance to avoid complications and unnecessary pain. Only few patients with OHVIRA are diagnosed during the neonatal period, prior to any clinical manifestation, usually following prenatal ultrasound diagnosis of a renal abnormality. Although the traditional description of OHVIRA syndrome includes renal agenesis, there is increasing evidence that this is not due to genuine absence of the kidney but rather to renal dysplasia and resorption. The cysts of multicystic dyspastic kidney (MCDK) may regress gradually, rarely prenatally, more commonly postnatally, resulting in a small dysplastic, or even absent, kidney, thus mimicking renal agenesis.

**Aims of the Study**: The purpose is to share our experience and focus attention on a high level of suspicion of OHVIRA during neonatal period when MCDK is detected in a female fetus prenatally.

**Methods**: We retrospectively assessed 142 fetal abdominal magnetic resonance imaging examinations from 2011 to 2024. We identified 6 female fetuses with MCDK, 4 of which were performed at 24 weeks, 1 at 27 weeks and 1 at 30 weeks of gestation.

**Results:** We present three cases of OHVIRA diagnosed postnatally. OHVIRA-syndrome is assumed in two of these neonates (didelphys uterus and no hydrocolpos found on ultrasound examination) and it was proven in one of them with uterus didelphys, obstruction of the ipsilateral vagina and involution of the MCDK following magnetic resonance urography at 10 months of age.

**Discussion**: The presence of MCDK during antenatal ultrasound examination of a female fetus, specifically when this involutes and eventually resembles renal agenesis should raise a high level of suspicion of OHVIRA syndrome.

**P42. Prevalence of Autoimmune Comorbidities in a Longitudinal Cohort of Children with Premenarchal Vulvar Lichen Sclerosus**

**C. Lepore ^1^, M. Wang ^2^, L. Sagnella ^1^, K. Miclette ^1^, A. Smith ^1^, A. Vash-Margita ^1^**

^1^  Yale School of Medicine, New Haven, CT, USA
^2^  University of California San Francisco, San Francisco, CA, USA

**Introduction and Aims:** Vulvar lichen sclerosus (VLS) has a 1 in 900 prevalence in premenarchal children. There are limited longitudinal studies of premenarchal VLS and its comorbidities. In this study we aimed to identify comorbidity prevalences among pediatric VLS patients, diagnosed between 2013–2020, compared to their published prevalences in the United States’ (US) pediatric population according to recent studies.

**Methods:** Through electronic record review, we analyzed 39 patients from a tertiary children’s hospital with clinically-confirmed VLS. Prevalence of autoimmune and psychiatric conditions and latest documented vulvar exam were identified. Controls were defined as published disease prevalences in the US population aged 0–18 years. Descriptive statistics and Chi square analysis were performed for categorical variables. This study was approved by the institutional review board.

**Results and Discussion:** Our cohort included 39 premenarchal patients with VLS. Current mean age was 12.4 years (SD = 2.9), and average time from VLS diagnosis to experimental follow-up was 5.3 years (SD = 1.6). VLS patients in our cohort were more likely to have eczema, asthma, vitiligo, and celiac disease (Table 6) compared to the general pediatric population in the US (*p* < 0.05). These patients were more likely to present with morphea: a rare dermatologic condition (*p* < 0.05). Mood disorders, (anxiety, depression, and adjustment disorder), and Hashimoto thyroiditis had similar prevalences to the general population. Follow-up vulvar physical exam was documented as improved for 38% of patients; high loss to follow-up (20.5%) may confound this value.

**Conclusions:** Longitudinal follow-up of VLS patients demonstrated an increased prevalence of autoimmune diseases including morphea in the 4–11-year range from VLS diagnosis encapsulated in this study. Provider awareness of these findings will support prompt identification and treatment of comorbidities. Prospective studies involving larger study samples are indicated.

**P43. Mixed Gonadal Dysgenesis in Genetic, Endocrinological, Anatomical, Reproductive, Psychological and Ethical Aspects—A Case Report**

**M. Krzyścin ^1^, K. Gruca-Stryjak ^2,3,4^, E. Sowińska-Przepiera ^1,5^**

^1^  Pediatric and Adolescent Gynecology Clinic, Department of Gynecology, Endocrinology and Gynecological Oncology, Pomeranian Medical University in Szczecin, Szczecin, Poland
^2^  Department of Perinatology and Gynecology, Poznań University of Medical Sciences, Poznań, Poland
^3^  Centers for Medical Genetics GENESIS, Poznań, Poland
^4^  Department of Obstetrics and Gynecology, “Polish’s Mother’s Memorial Hospital: Research Institute, Łódź, Poland
^5^  Department of Endocrinology, Metabolic and Internal Diseases, Pomeranian Medical University in Szczecin, Szczecin, Poland

**Introduction:** Sex determination, a fundamental aspect of human biology, is primarily determined by the presence or absence of the sex determining region of the Y gene. The mosaic nature of chromosomal variations can lead to unique and troubling cases that challenge our conventional understanding of sexual development.

**Aim:** To present the complexity of the problem in a child with mixed gonadal dysgenesis (MGD).

**Methods:** Prenatal testing at 12 weeks’ gestation indicated significant fetal developmental abnormalities, and genetic amniocentesis revealed an abnormal 45X0/46XY mosaic karyotype. At birth, the baby’s phenotype included features of facial dysmorphia, a neck with a membrane, and hermaphroditic external organs. Testosterone and antimüllerian hormone were increased. Imaging showed a left testis, a right-sided urogenital strip and the presence of uterus. Cystoscopy revealed well-developed Müllerian structures: vagina about 5 cm long and cervix. Genetic testing in neonate confirmed the presence of the SRY gene. In the blood, a mosaicism of 45X0/46XY (50/50) was obtained and in a smear of buccal epithelium the distribution was (52/48). The parents were informed of the unique difficulties in unambiguously determining the child’s sex. Based on the appearance of the external genitalia and the levels of hormones at birth, it was suggested that the appropriate sex for the child appeared to be male. However, the parents decided to raise the child as a girl.

**Discussion:** The presented case highlights the complexity of the problem—clinical, diagnostic, psychological and ethical implications in an infant with confirmed MGD.

**Conclusions:** MGD requires a comprehensive evaluation including genetic, endocrinological, anatomical, reproductive and psychological aspects and individualized multidisciplinary approach. The percentage of mosaicism can be divergent in different tissues of the body. Psychological support for parents is crucial, given the multifaceted decisions they have already had to make and will face in the future.

**P44. A Rare Congenital Uterine Anomaly: The Role of 3D Ultrasound**

**G. Di Paolo ^1^, C. Silvi ^2^, M. Bernassola ^3^, M. Francesca ^4^**

^1^  Studio Madre Perla, Pediatric and Adolescent Gynecology Center, Pescara, Italy
^2^  Department of Obstetrics and Gynecology, Renzetti Hospital, Lanciano, Italy
^3^  Department of Obstetrics and Gynecology, G. D’Annunzio University of Chieti, Chieti, Italy
^4^  Department of Obstetrics and Gynecology, Sant’Omero Hospital, Teramo, Italy

**Introduction and Aim of the Study**: Septate uterus is the most common congenital anomaly of female genital tract. However, other malformations, such as complete septate uterus with double cervix, are very rare and pose a challenge in understanding their embryological origins and defining therapeutic strategies Our aim is to contribute to the reports of all uncommon cases of Mullerian anomalies and to highlight the role of 3D ultrasound in diagnosing uterine malformations.

**Methods:** The patient was evaluated at Madre Perla clinic in Pescara (SIGIA regional referral center) by medical history, gynecologic examination, TA/TV 2D/3D ultrasound and hysteroscopy.

**Discussion:** we report the case of a 24-year-old patient who came for dysmenorrhea and infertility. The patient reported no family and personal pathological history, menarche at 11-yo with regular cycles, no AUB, no other symptoms. At gynecological examination with speculum a regular vaginal canal and two cervixes separated by an incomplete septum of about 1 cm were observed. At 2D ultrasonography, two separate endometrial lines were visualized in cross-section. However, 3D ultrasonography clearly showed a single uterine body with regular external profile, two endometrial cavities separated by complete septum and double cervical canal. This observation enabled us to exclude a didelphys uterus and make a diagnosis of complete septate uterus, double cervix and incomplete vaginal septum (ASMR), classifiable as U2bC2V1 according to the current ESHRE-ESGE classification. No further urogenital malformations were found. The patient then underwent hysteroscopy, which confirmed the presence of two endometrial cavities divided by complete septum and double cervical canal.

**Conclusions:** 3D ultrasonography is a useful, rapid, safe and sensitive tool in detecting uterine malformations, as it provides information on external contours, cavity morphology and thickness of septum and myometrial wall (useful for therapeutic approach). Therefore, in experts hands, it could replace MRI and diagnostic laparoscopy.


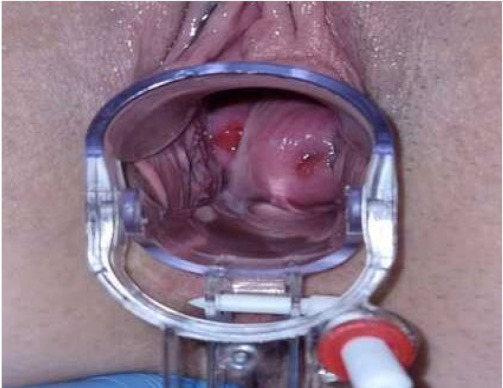



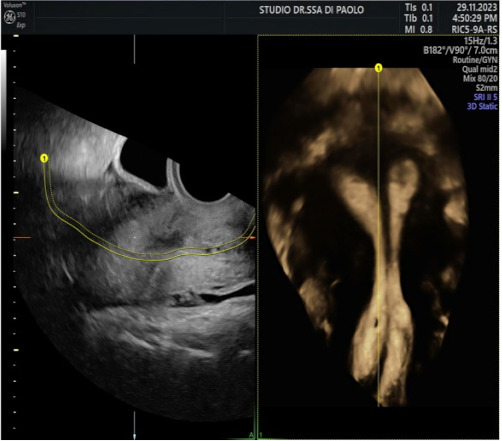

**P45. The Impact of Female Genital Mutilation (Fgm) on Mental Health**

**K. Paschopoulou, A. Vatopoulou, V. Siafaka, M. Paschopoulos**

University Of Ioannina, Ioannina, Greece

**Introduction and Aims of the Study:** More than 200 million girls and women alive today, 600,000 of which are estimated to live in Europe, have undergone FGM in 31 countries across three continents. 1/3 have developed a form of psychopathology connected to the experience. The aim is to explore the relationship between the experience of FGM and the psychological burden the girls and women develop and present the adverse mental health consequences s associated with the practice.

**Methods:** A review of the last 5 years literature was conducted, in order to identify the short and long-term psychological effects connected to FGM, as well as the possible improvement after reconstructive surgery.

**Results:** The complications of FGM include medical, menstrual and sexual problems, but some of the most prominent ones are the severe psychological damage girls and women face connected to the experience. Psychological distress includes but is not limited to depression, anxiety, feelings of isolation, post-traumatic stress disorder, somatization, phobia, sleep disorders, fear, suppression of emotions and low self-esteem. The consequences are directly affected by the age of cutting and the overall experience. The reasons behind the adaptation of the practice are mainly gender inequality, social pressure and cultural attitudes. The belief that FGM has religious support is no longer valid, since numerous religious leaders are contributing to its abandonment.

**Conclusions:** FGM has zero correlation with geographical boundaries due to the large amount of mobile populations. Every year at least 20,000 women and girls are coming to Europe from FGM-risk countries as asylum seekers, many of them in Greece specifically. FGM is a form of physical and emotional abuse, as a result, psychological interventions should always be included in the healthcare of girls and women who have undergone the procedure. More rigorous research on the topic is needed.

**P46. Pregnancy and Labour Outcomes in Greek Roma Adolescents**

**A. Samara ^1^, D. Stamouli ^2^, S. Tsiapakidou ^3^, P. Christopoulos ^4^, S. Sotiriou ^1^, K. Dafopoulos ^2^**

^1^  University of Thessaly, Larissa, Greece
^2^  University Hospital of Larissa, Larissa, Greece
^3^  Papageorgiou General Hospital of Thessaloniki, Thessaloniki, Greece
^4^  Areteion Hospital of Athens, Athens, Greece

**Introduction and Aims of the Study:** Pregnancy during adolescence is associated with an increased risk of adverse maternal and neonatal outcomes. Greek Roma communities are characterized by low education attainments and cultural pressure for marriages during adolescence. The aim of the present study is to investigate the outcomes of pregnancy in a special social group of Greek Roma pregnant adolescents.

**Methods:** All Greek Roma pregnant adolescents that gave birth in a single tertiary hospital during a 24-months period were enrolled in the present study. A retrospective analysis of medical records was performed.

**Results:** In total, 130 adolescents with a median age of 17 years old (range: 13–19) were included in our analysis. The majority of the cases (68%), were experienced their first pregnancy, 25% their second pregnancy and 7% were multiparous. Moreover, the preterm birth rate was calculated at 12%, with sixteen pregnancies being terminated before the 36th week of gestation. A finding of interesting is that six cases (5%) had never visited an obstetrician before the labour day. Regarding the labour, more than the half (59%) of the pregnancies were delivered with a cesarean section (CS) with the two thirds being elective deliveries. The main reason of CS (28 cases—36%) was the request of the adolescence based on their unwillingness to experience a vaginal labour, following by a previous CS (16 cases-21%), 15 cases (19%) of non-progression of labour, 8 cases (11%) of fetal distress, 7 cases (9%) of breech presentation, and 3 (4%) cases of other maternal comorbidities. Regarding complications during pregnancy, 13 adolescents (10%) experienced a major complication during pregnancy including 12 cases of intrauterine growth restriction or preeclampsia and a case of placenta previa.

**Conclusions:** Roma pregnant adolescents are a special group of patients requiring special care and advising.

**P47. Signs and Symptoms of Precocious Puberty: Updated Data from a Tertiary Level Center in Northern Greece**

**A. Pana, E. Iordanidou, E. Deligeoroglou, L. Zepiridis, T. Theodoridis, G. Grimbizis**

Aristotle University of Thessaloniki, Thessaloniki, Greece

**Introduction:** Precocious puberty is commonly defined as puberty that starts before the age of 8 in girls. Signs can include breast growth, pubic hair, rapid bone maturation, and acceleration of growth. In many cases, no cause can be found for the disorder.

**Aim:** The objective of this study was to collect and present data, with respect to signs and symptoms, from patients referred to a pediatric and adolescent gynecology tertiary level center in Northern Greece following the initial diagnosis of precocious puberty.

**Methods:** A retrospective analysis of prospectively collected data from all female patients with signs and symptoms of precocious puberty was performed between January 2014 and January 2024.

**Results:** A total of 46 patients were retrieved from the archives. Mean age at presentation was 7.3 years of age (2–8). 30 patients (65.2%) were 8-year-olds, 9 (19.6%) were 7-year-olds, 4 (8.7%) were 6-year-olds, 2 (4.3%) were 5-year-olds, and 1 (2.2%) was 2-year-old. In 18 (39.1%) patients the presenting symptom was adrenarche, in 17 (37%) thelarche, in 8 (17.4%) vaginal bleeding, and in 3 (6.5%) body odour. 10 patients (21.7%) were diagnosed with idiopathic precocious puberty and in 6 (13%) therapeutic interventions were deemed necessary to mitigate potentially negative psychosocial impacts. Treatment with GnRH analogues was applied in all 16 cases with a six month follow-up until patients reached 11 years of age. Mean time of menarche following therapy was 12 months (6–24).

**Conclusions:** Concerns about early pubertal development are a common cause of parental anxiety in pediatric and adolescent gynecology departments. Only a small number of patients have precocious puberty and require thorough testing and treatment, whereas the great majority of cases have benign, normal variants of puberty that need to be followed by health care providers.

**P48. Diagnosis and Management of Adnexal Lesions in Adolescents: Updated Data from a Tertiary Center in Greece**

**E. Iordanidou, A. Pana, C. Anthoulakis, K. Chatzistamatiou, D. Tsolakidis, T. Theodoridis, G. Grimbizis**

1st Department of Obstetrics & Gynecology, Aristotle University, Thessaloniki, Greece

**Introduction:** Adnexal lesions are the most frequent gynecological pathology seen in adolescent and young girls. The clinical presentation is quite variable and torsion is the most frequent complication. Although most cystic lesions are benign, malignancy must be eliminated. Surgical treatment should always follow oncological standards and be as conservative as possible to preserve future fertility.

**Aim:** The aim of this study is to present the clinical features of adolescents girls with adnexal lesions who were treated surgically in a tertiary gynecological center in Greece.

**Methods:** A retrospective analysis of prospectively collected data from all female patients aged 18 years old or less, who were operated upon with a diagnosis of adnexal lesion, was performed from January 2008 to December 2023.

**Results:** A total of 63 patients were retrieved from the archives. The mean age at presentation was 15.6 years (range 12–18). In 20 patients (31.74%), the leading symptom was abdominal pain. Acute abdomen was evident in only 6 patients (9.52%). All patients were assessed with pelvic ultrasonography. Pelvic MRI was utilized in 18 patients (28.57%) to aid the diagnosis. In 37 patients (58.7%) the adnexal lesion was an incidental finding in pelvic ultrasonography. Adnexal torsion was suspected and subsequently surgically confirmed in 10 patients (15.87%). 7 patients (11.11%) had a unilateral salpingo-oophorectomy and 56 patients (88.88%) had a cystectomy. Pathology revealed 4 malignant cases (6.34%), whereas 59 cases (93.65%) were deemed as benign. From the latter, 18 patients (28.57%) had functional ovarian cysts.

**Conclusions:** Adnexal lesions are mostly benign and their frequency increases with age. Ultrasonography is the diagnostic tool of choice, whereas MRI is an essential complement to aid the diagnosis. Fertility-sparing surgery should be the standard management to maintain ovarian function and to improve quality of life in this particular group of individuals.

**P49. Vaginal Foreign Body Evaluation and Treatment: A Case Report**

**A. Pana, E. Iordanidou, A. Sarieva, I. Theodoulidis, D. Tsolakidis, T. Theodoridis, G. Grimbizis**

Aristotle University of Thessaloniki, Thessaloniki, Greece

**Introduction**: Vaginal foreign body (VFB) may be asymptomatic or present with an array of symptoms, including pelvic pain, vaginal discharge, or vaginal bleeding. VFB may result from ignorance, accident, psychotic tendencies and attempts of sexual stimulation or sexual abuse. There is a broad differential diagnosis including malignancy, sexually transmitted infections and pregnancy.

**Aim**: The aim of the study is to present the case of a 12-year-old girl with VFB and thus highlight common pitfalls in the clinical as well as differential diagnosis.

**Method**: A 12-year-old girl presented to the emergency department under parental supervision in view of persistent abdominal pain. The patient was afebrile whereas clinical signs and symptoms were inconclusive, thus the patient was refereed to the paediatric and adolescent gynecology department for subsequent evaluation. Detailed history was of no added value but laboratory tests were indicative of inflammation. Her parents denied any history of trauma or sexual abuse. Ultrasonography did not aid the diagnosis. A CT scan was performed and a suspicious mass (6.5 cm × 5.5 cm × 6 cm) between the rectum and the bladder was revealed.

**Results**: The patient ultimately underwent an examination under anesthesia and vaginoscopy with removal of a single VFB. The latter was identified as plastic bottle cap. During vaginoscopy, vaginal wall erosion was detected and stitches were placed in order to restore and reinforce the structure. Following the removal of the VFB, all symptoms were immediately alleviated. In order to exclude the possibility of sexual abuse, social workers and psychiatrists interviewed the child and the parents both individually and in groups. Conclusively, sexual abuse was excluded. The postoperative period was uneventful and the patient was discharged from hospital 3 days after surgery.

**Conclusions**: Retained VFBs pose significant health risks and may present with diverse symptoms. VFBs should be considered in young female patients presenting with persistent or recurrent vaginal discharge. Sexual abuse must always be excluded when dealing with these patients.

**P50. Frequency and Obstetrical Complications of Teenage Pregnancy: The Experience of a Tertiary Reference Center**

**E. Iordanidou, A. Pana, E. Deligeoroglou, T. Mikos, E. Bili, T. Theodoridis, G. Grimbizis**

1st Department of Obstetrics & Gynecology, Thessaloniki, Greece

**Introduction:** Adolescent pregnancy is considered a public health issue by the World Health Organization (WHO) that adversely affects birth outcomes and can lead to intergenerational cycles of poverty and ill-health.

**Aim:** The aim of the study is to present the clinical features of teenage pregnancy cases managed in a tertiary pediatric gynecology center in Greece

**Materials and Method:** A retrospective analysis of perspectively collected data was performed in hospital archives from January 2008 to December 2023. The total number of adolescent pregnancies was recorded as well as epidemiological characteristics, maternal age, mode of delivery, birthweight, incidence of preeclampsia and premature birth.

**Results:** The analysis revealed a total of 762 teenage pregnancies, which constitute 2.5% of all births (30,000) in the aforementioned time period. The mean maternal age was 15.2 years (range 12–18). Further subdivided, Group A (15–18 years old) consisted of 628 patients (82.42%) and Group B (12–15 years old) consisted of 134 patients (17.58%). With respect to ethnicity, 146 (19.16%) were Roma Muslims and 134 (7.58%) were immigrants and refugees. 480 patients (62.9%) experienced their 1st pregnancy, 161 (21.12%) their 2nd pregnancy, and 31 (4.06%) their 3rd pregnancy. Preterm labour was recorded in 125 pregnancies (16.4%) and preeclampsia complicated 15 pregnancies (1.96%). 515 patients (67.58%) had a vaginal delivery, whereas 247 patients (32.41%) had a caesarean section. In 34 (6.6%) vacuum-assisted vaginal delivery was indicated. Subgroup analysis revealed no difference in birthweight between Group A and Group B. However, regarding Group B, preterm birth was recorded in 13 patients (9.7%) and preeclampsia in 44 patients (32.8%), indicating that obstetric complications are potentially related to maternal age.

**Conclusions:** Teenage pregnancy is related to socioeconomic factors. The aim is to inform young people about available contraceptive methods in order to reduce the incidence of the phenomenon.

**P51. Gitelman’s Syndrome Diagnosis in an Adolescent Girl During the First Trimester of Pregnancy: A Case Report**

**K. Zacharis, C. Alexakis, I. Anagnostaki, V. Tsapadikou, S. Chondros, A. Barmparousi, E. Chrysafopoulou, S. Kravvaritis, A. Fouka, T. Charitos**

Department of Obstetrics and Gynecology, General Hospital of Lamia, Lamia, Greece

**Introduction:** Healthy period cycle is the crucial aspect of menstrual health (MH), which is a state of complete physical, mental, and social well-being and not merely the absence of disease or infirmity, in relation to the menstrual cycle.

**Aim:** to assess the role of individual factors of sex education and qualitative characteristics of menstrual function at formation of positive attitude towards menstruation in adolescent girls.

**Methods:** Research was performed from May to December 2022 at Almazov National Medical Research Centre (Saint Petersburg, Russia). We surveyed 224 girls about main aspects of MH and menstrual hygiene (MHG). Questionnaire contained 24 questions, divided into 3 groups: general information, evaluation of menstrual cycle, MHG features.

**Results:** Patients were divided in groups with positive (group 1, n = 173) and negative (group 2, n = 51) attitude to menstruation. Mean age: group 1—15.1 ± 1.6 y.o., group 2—14.8 ± 1.8 y.o. (*p* = 0.307). Mean menarche age: group 1—12.3 ± 1.3 years, group 2—11.8 ± 1.1 years (*p* = 0.023). In group 1 mother was more often the primary source of information about MH (83.2 versus 68.6% respectively), at the same time other sources were more frequently mentioned in group 2 (27.5% versus 13.9% respectively) (χ^2^ = 5.36, *p* = 0.021). Patients of group 2 more often suffered from excessive menstrual flow: group 1–67 (38.7%), group 2–31 (60.8%) (*p* = 0.006). Girls in group 2 had more often difficulties in MH in public places (group 1–9 (5.2%), group 2–12 (23.5%) (*p* = 0.001)) and no possibilities to MH implementation at school (group 1–5 (2.9%), group 2–7 (13.7%) (*p* = 0.002)).

**Conclusions:** According to our study the following factors form positive attitude towards menstruation among girls: later onset of menarche, active involvement of mother at education process and on-time informing about menstruations, formed family culture of menstrual calendar use, moderate menstrual bleeding, absence of problems in MH at school, possibilities to disposal of MH products in public places.

**P52. Complete Androgen Insensitivity Syndrome (Cais): Hormone Replacement Therapy (Hrt) Options and Quality of Life (QoL)**

**N. Vozaiti ^1^, M. Tsiriva ^2^, L. Vogiatzi-Vokotopoulou ^2^, E. Charmandari ^1^, G. Daskalakis ^2^, L. Michala ^2^**

^1^  Division of Endocrinology, Metabolism and Diabetes, First Department of Pediatrics, National and Kapodistrian University of Athens Medical School, ‘Aghia Sophia’ Children’s Hospital’, Athens, Greece
^2^  First Department of Obstetrics and Gynaecology, National and Kapodistrian University of Athens Medical School, ‘Alexandra General Hospital’, Athens, Greece

**Introduction:** CAIS is a difference of sex development (DSD) characterized by generalized, complete tissue insensitivity to androgens, resulting in a fully feminine phenotype despite the presence of a 46XY karyotype and normal testicular tissue. Gonadectomy has traditionally been offered following completion of puberty due to risk of malignancy. However, trends towards gonadal preservation beyond puberty have been emerging owing to the diminished sense of wellbeing following surgery.

**Aim:** The aim of this review was to assess physical and psychological wellbeing in subjects with CAIS who have undergone gonadectomy.

**Methods:** A literature review was conducted in Pubmed, Scopus and Google Scholar for studies from 2014 onwards, using the terms ‘CAIS’ and ‘wellness’, ‘well-being’, ‘sexual function’, ‘mental health’, ‘HRT’.

**Results:** A total of 6 studies of gonadectomized CAIS individuals were included. One cross-sectional study of 113 women investigated patients’ preferences over HRT alternatives, indicating that the vast majority preferred oral or transdermal estrogen use over testosterone administration. Three studies assessed sexual function in 71, 66 and 11 women, respectively, with the last one investigating also the overall QoL and presence of psychological distress. The sexual life of women with CAIS, who had undergone gonadectomy, was impaired, with lack of sexual confidence and infrequency reaching up to 66%. Psychological distress is also increased based on calculation of standardized instrument scores. Finally, a crossover trial showed no improvement in QoL in women receiving testosterone, except for sexual desire.

**Conclusions:** Psychological distress and sexual dysfunction were found to be increased in gonadectomized CAIS women. HRT, with the addition of testosterone does not fully improve wellbeing. Counselling prior to gonadectomy should include this information, in order to enable balanced decision-making and informed consent prior to surgery.

**P53. A Large Vulvar Epidermoid Cyst in an Adolescent Girl with a History of Cloaca Malformation Reconstruction**

**E. Paschalidou, A. Matsas, E. Tsarna, O. Triantafyllidou, N. Vlahos, P. Christopoulos**

Division of Pediatric and Adolescent Gynecology, 2nd Department of OBGYN, Medical School, National and Kapodistrian University of Athens, Athens, Greece

**Introduction:** Epidermoid cysts in the vulva are uncommon in adolescent girls and most commonly involve clitoris. They are slowly growing, intradermal or subcutaneous tumors with a wall composed of true epidermis. They are usually associated with previous trauma or female genital mutilation leading to invagination of squamous epithelium and then desquamating into a closed space to form a cystic mass. The majority of vulvar epidermoid cysts do not require treatment, unless they become infected, painful, or enlarged.

**Case Description:** A 20-years-old girl visited our outpatient department with a complaint of a painful and palpable mass in her vulva that has recently increased in size and interfered with the patient’s daily activities (Figure 18). Clinical examination revealed a large, mobile, tender, and soft mass with smooth contour located in the right labial minora and vulva. The magnetic resonance imaging (MRI) revealed a large cystic mass in right labial minora, 9.5 cm in diameter without contrast enhancement. The patient had a history of prophylactic colostomy due to rectal atresia during infancy, followed by posterior sagittal anorectal vaginal urethroplasty (PSARVUP) and colostomy closure for persistent cloacal malformation. Puncture of the cyst and needle aspiration was performed and the cytological examination of the content was consistent with a vulvar epidermoid cyst. Excision of the cyst has been proposed.

**Conclusions:** Epidermoid cysts of the vulva are uncommon and MRI is a valuable imaging technique to define their characteristics and provide valuable information on the possible etiology. Treatment options include local heat application, incision and drainage, or excision. Although vulvar masses in adolescents are rare, differential diagnosis should include epidermoid cysts.

**P54. The Time Trend of Age at Sexual Debut in Greece: An Extended Analysis**

**E. Tsarna ^1,2^, M. Marasioni ^2^, G. Kostomoiris ^1^, A. Katsaka ^2^, A. Valla ^2^, P. Christopoulos ^2^**

^1^  Department of OBGYN, General Hospital of Nikaia “Agios Panteleimon, Athens, Greece
^2^  Division of Pediatric and Adolescent Gynecology, 2nd Department of OBGYN, Medical School, National and Kapodistrian University of Athens, Athens, Greece

**Introduction and Aims of the Study:** Sexual health studies conducted in developed countries have indicated a decrease in age at sexual debut during the last 50 years, especially among women. The aim of this study was to describe the time trend of age at sexual debut among women in Greece.

**Methods:** In this cross-sectional study among women that visited the outpatient OB/GYN offices from 2017 to 2023, we calculated the non-parametric Spearman rank correlation coefficient (rs) between age at sexual debut and year of birth, as these were self-reported during structured interviews. Mean age at sexual debut was calculated per decade of birth among total study population and in nationality subgroups with sample sizes greater than 50.

**Results and Discussion:** Our total study population was 3507 women, among which rs was −0.24 (*p* < 0.01), reflecting a decline in age at sexual debut from 21 years among women born in 1930s to 16.2 years among those born in 2000s. Similarly, among women with Greek nationality rs was −0.26 (*p* < 0.01, n = 2305), among women of Albanian nationality rs was −0.35 (*p* < 0.01, n = 316), among women of Central/Eastern European nationalities rs was −0.28 (*p* = 0.02 with n = 73), and among women of former USSR nationalities rs was −0.18 (*p* = 0.01, n = 195). The association was not significant only among women of Middle East and North African nationalities (rs = −0.27, *p* = 0.06), which was the smallest subgroup (n = 50).

**Conclusions:** Age at sexual debut has declined significantly with time in Greece during the last half of 20th and 21st century. Nonetheless, the magnitude of this decline differs across nationality subgroups underlining the role of socioeconomic and cultural factors, which are not homogeneous across groups of women with different nationalities even when residing within the same country.

**P55. Age at Sexual Debut and Level of Education Among Women in a Socioeconomically Deprived Population in Greece: An Extended Analysis**

**O. Triantafyllidou ^1^, M. Marasioni ^2^, E. Tsarna ^1,2^, A. Matsas ^1^, N. Vlahos ^1^, P. Christopoulos ^1^**

^1^  Division of Pediatric and Adolescent Gynecology, 2nd Departement of OBGYN, Medical School, National and Kapodistrian University of Athens, Athens, Greece
^2^  Department of OBGYN, General Hospital of Nikaia “Agios Panteleimon, Athens, Greece

**Introduction and Aims of the Study:** Age at sexual debut is influenced by poor family status, low socioeconomic and educational levels, as well as illicit substance abuse and coercion. Good school attendance and participation are associated with a later age at sexual debut. The aim of this study was to explore the association of educational level with age at sexual debut among women in a socioeconomically deprived population in Greece.

**Methods:** Self-reported age at sexual debut and educational level from 3071 women that visited the outpatient OB/GYN offices between 2017 and 2023 were analyzed. The non-parametric Kruskal-Wallis Rank Sum Test was applied among six hierarchical educational levels and the non-parametric Dunn’s test with Bonferroni correction was used for pairwise comparisons.

**Results and Discussion:** Mean age at sexual debut increased with higher educational level (*p* < 0.01), ranging from 17.0 (±3.4) years among women that have not concluded the nine years of compulsory education in Greece to 18.8 (±2.4) years among women with an MSc/PhD. Notably, the aforementioned association was statistically significant, even though women of higher educational level were born on average later (*p* < 0.01) and age at sexual debut was negatively correlated with year of birth (*p* < 0.01). Based on results from pairwise comparisons, women who never completed compulsory education differed significantly from all other groups and women who attended nine years of school differed significantly from most other groups. Minimal or no differences in age at sexual debut were detected among women who attended any kind of post-secondary education.

**Conclusions:** In our study, higher educational level was significantly associated with higher age at sexual debut, but this association attenuated for women who later on in their lives followed any kind of post-secondary education.

**P56. A Severe Case of Vulvovaginitis Requiring Hospitalisation and Systemic Treatment in a Young Female**

**K. Karkalemis, A. Morfiadaki, E. Tsarna, E. Karopoulou, N. Vlahos, P. Christopoulos**

Division of Pediatric and Adolescent Gynecology, 2nd Department of OBGYN, Medical School, National and Kapodistrian University of Athens, Athens, Greece

**Introduction:** Vulvovaginitis may present with excessive external genitalia edema disturbing urination and severe inflammation leading to systemic symptoms, such as fever.

**Case Presentation:** A 22-years-old patient visited the dermatology outpatient office due to severe genital pruritus, purulent discharge, excessive external genitalia edema causing minor labial adhesion and severe pain disturbing urination with progressive worsening of symptoms since three days. Regular sitz baths with Triclosan 0.4% solution and application of octenidine hydrochloride-enriched gauzes and amikacin 5% gel on the affected area were advised. Due to exacerbation of the inflammation and fever up to 38.5 °C, the patient was referred for hospitalization and systemic intravenous treatment in the gynecology clinic. Blood, urine, and vaginal discharge cultures were obtained, including testing for chlamydia, gonorrhea, and recent herpes infection. Following consultation with a dermatologist, a single intramuscular betamethasone dose was administered, while intravenous treatment with clindamycin and ciprofloxacin due to amoxicillin allergy and local application of amikacin gel were initiated. Paracetamol was administered to control fever for three days, while parecoxib and ibuprofen were administered against inflammation and for pain relief. Due to clinical presentation highly suspicious for herpes infection, valacyclovir was additionally administered for seven days. Progressive improvement of the external genitalia edema was noted. On the 6th day of hospitalization, vaginal discharge culture results showed growth of ureoplasma and streptococcus agalactiae, while the remaining tests were negative, including herpes. The patient was discharged on the 11th day, advised to continue per os antibiotics for 10 more days. She presented free of symptoms at the outpatient office for follow-up examination.

**Conclusions:** Appropriate treatment of severe cases of vulvovaginitis may require hospitalization and cooperation between multiple specialists for diagnostic management and systematic therapy.

**P57. Contraception Use Among Adolescents and Young Adults in a Socioeconomically Deprived Region of Greece: An Extended Analysis**

**E. Tsarna ^1,2^, G. Kostomoiris ^1^, G. Gkyrti ^2^, G. Karampas ^2^, N. Vlahos ^2^, P. Christopoulos ^2^**

^1^  Department of OBGYN, General Hospital of Nikaia “Agios Panteleimon, Athens, Greece
^2^  Division of Pediatric and Adolescent Gynecology, 2nd Departement of OBGYN, Medical School, National and Kapodistrian University of Athens, Athens, Greece

**Introduction and Aims of the Study**: Method of contraception among adolescents and young adults varies significantly across geographical regions within Europe. The aim of this study was to describe contraception use among female adolescents and young adults in a socioeconomically deprived population in Greece.

**Methods:** All women up to 25 years old that visited the outpatient OB/GYN offices between 2017 and 2023 were included in this study. Self-reported contraception use and contraceptive methods chosen were analyzed and Chi-squared test and Kruskal-Wallis Rank Sum Test were applied to compare baseline characteristics between users and nonusers.

**Results and Discussion:** Out of 544 women, 499 (91.7%) reported using some form of contraception. Nonusers were less likely to be insured (*p* < 0.01), less likely to have Greek nationality (*p* < 0.01), were of lower educational level (*p* < 0.01), more likely to be married (*p* < 0.01) and have children (*p* = 0.03), and less likely to smoke (*p* = 0.04). Regarding contraceptive methods, male condoms were used by 61.7% of our study population, the withdrawal method by 51.7%, emergency hormonal contraception by 14%, oral contraceptives by 9.8%, and intrauterine devices by 3.6%. Male condom use was associated with being insured, being single or in a non-cohabitating relationship, nulliparity, a higher educational level, and an older age at sexual debut. In contrast, withdrawal method was associated with lack of insurance, being married, having already children, having a lower educational level, more frequent smoking, younger age at sexual debut, and being less frequently vaccinated against HPV.

**Conclusions:** Among a socioeconomically deprived population in Greece, the vast majority of women up to 25 years old used some form of contraception; most frequently male condoms and withdrawal method, despite its lack of effectiveness, followed by emergency contraception and oral contraceptives.

**P58. A Rare Case of Ovarian Mucinous Borderline Tumor in an Adolescent Patient Presenting as a 25 cm Pelvic Mass**

**A. Morfiadaki, K. Karkalemis, E. Tsarna, E. Panagodimou, N. Vlahos, P. Christopoulos**

Division of Pediatric and Adolescent Gynecology, 2nd Department of OBGYN, Medical School, National and Kapodistrian University of Athens, Athens, Greece

**Introduction and Aim of the Study:** Borderline mucinous tumors are rare in adolescents and have excellent prognosis. The aim of this study is to present a case of ovarian borderline mucinous tumor in a 17 year old adolescent, emphasizing in the clinical presentation and the applied treatment.

**Case Presentation:** Patient visited the emergency department due to exacerbation of atypical pelvic pain and bloating with the onset of menstruation. The aforementioned symptoms were present the last 3 months. A visible abdominal mass was noted; the pelvic MRI revealed a 25 × 21 cm multilocular cystic mass originating from the left ovary, reaching the L1 vertebrae and causing distention of the left renal pelvis. No pelvic or para-aortic lymph nodes were enlarged, while ovarian cancer blood markers were within normal range. During laparotomy, the ovarian mass was excised intact while sparing healthy ovarian tissue. After fast-track biopsy indicating mucinous borderline tumor, appendectomy and partial omentectomy were performed. Pathology report confirmed the aforementioned diagnosis (Stage IA). After oncologic consultation, oocyte cryopreservation and pelvic MRI at six months post-operatively were advised. The pelvic MRI revealed a normal-sized right ovary, a slightly atrophic left ovary and absence of any pelvic pathology. At 18 years of age, patient underwent natural cycle oocyte retrieval for cryopreservation with adequate response.

**Conclusions:** This rare case of ovarian mucinous borderline tumor in adolescence highlights that symptoms of pelvic masses even of impressively large size remain non-specific in teenage patients. To preserve fertility, surgical treatment and management of such tumors in adolescence is important to be carried by an experienced multi-disciplinary team.


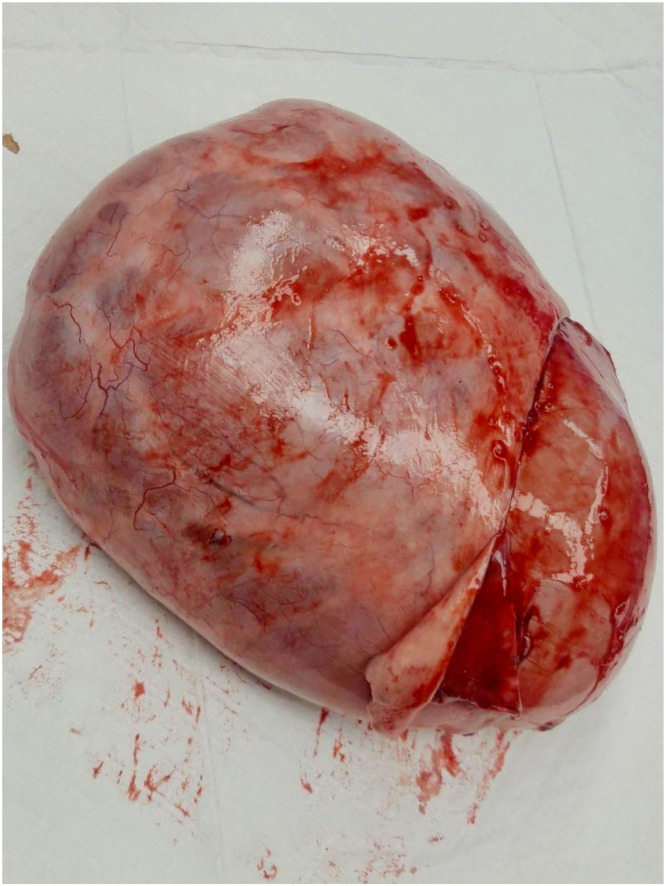

**P59. The Association of Age at Menarche with Sexual Debut in Greece: An Extended Analysis**

**A. Valla ^2^, E. Tsarna ^1,2^, G. Kostomoiris ^1^, M. Marasioni ^1^, A. Katsaka ^2^, P. Christopoulos ^2^**

^1^  Department of OBGYN, General Hospital of Nikaia, “Agios Panteleimon, Athens, Greece
^2^  Division of Pediatric and Adolescent Gynecology, 2nd Department of OBGYN, Medical School, National and Kapodistrian University of Athens, Athens, Greece

**Introduction and Aims of the Study:** Age at menarche has been positively associated with age at sexual debut and high-risk sexual behavior in middle- and low-income countries. Along with psychosocial and cultural factors, a direct biological association is hypothesized since sexual hormones are a prerequisite for sexual desire. The aim of this study was to evaluate the association of age at menarche with sexual debut in Greece.

**Methods:** In this cross-sectional study of women that visited the outpatient OB/GYN offices from 2017 to 2023, the non-parametric Spearman’s correlation coefficient (rs) was calculated based on self-reported age at menarche and age at sexual debut. Sensitivity analysis was performed in subgroups with more than 50 participants stratified by nationality and decade of birth.

**Results and Discussion:** Among 3111 study participants, rs was 0.13 (*p* < 0.01) with mean age at menarche being 12.7 and mean age at sexual debut being 18.3. Positive and significant rs was observed in the subgroups of Greek (n = 2334) and Albanian (n = 327) nationalities, but did not reach statistical significance in Central/Eastern European (n = 74) and former USSR (n = 203) nationalities subgroups. Similarly, rs was positive in all subgroups based on decade of birth, although results were not significant among women born in the 1940s and 1980s. Notably, the strongest association was observed among women of Albanian nationality (rs = 0.26) indicating the role of cultural factors and those born in 2000s (rs = 0.27), among whom recall bias is expected to affect less the results.

**Conclusions:** A weak, but consistent, correlation between age at menarche and sexual debut was observed in our study. Given that psychosocial and cultural factors differ wildly between countries where relevant research has been performed, the stability of this association implies a biological link.

**P60. Features of Retinoic Acid Signaling and Endometrial Receptivity in Genital Malformations and Endometriosis**

**Z. Batyrova, A. Asaturova, A. Magnaeva, E. Uvarova, V. Chuprynin, T. Ivanets, J. Kessler, T. Aksenova, Z. Kumykova**

FSBI, National Medical Research Center For Obstetrics, Gynecology and Perinatology Named After Academician V.I.Kulakov, Moscow, Russian Federation

**Introduction**: Retinoic acid plays a key role during vertebrate development. it has been shown that retinoids play a fundamental role in maintaining the normal functioning of the endometrium and the development of endometriosis.

**Aim of the Study**: To analyse peculiarities of retinoic acid signalling in genital malformation and endometriosis.

**Methods**: 40 patients: 20 with malformations of the genital organs and 20 control.

**Results**: The average levels of retinoic acid and retinol binding protein 4 in the group with confirmed endometriosis were significantly reduced compared to the group without it, moreover exceeding the average values determined in the control. The analysis of the staining intensity demonstrated lower H-score values of Er and Pr receptors in both the stroma and endometrial glands of the uterine horns compared the control. In addition, there was a violation of the ratio of Er/Pr receptors in the endometrium of the uterine horns—in the proliferation phase, the level of Er was lower than the level of Pr. The staining intensity demonstrated lower H-score values of the RxR alpha and beta b both in the stroma and in the endometrial glands of the uterine horns compared with the control. In the studied groups, the results demonstrated a direct relationship between the intensity of staining of Er markers and RxR alpha in both glands and endometrial stroma.In addition, the relationship between the intensity of Pr staining and RxR alpha found in glands and stroma, a close direct relationship was established. In the studied groups, a statistically significant direct correlation was found between the intensity of staining of Er and Pr receptor markers and RxR beta in the endometrial stroma.

**Conclusions:** Patients with genital malformations belong to the risk group for the development of endometriosis, due to the peculiarities of retinoic acid signaling disorders, which requires further study.

**P61. Adolescent Pregnancies and Deliveries in a General Hospital in Athens, Greece**

**E. Tsarna ^1,2^, M. Marasioni ^1^, N. Georgopapadakos ^1^, D. Balafoutas ^2^, E. Karopoulou ^2^, P. Christopoulos ^2^**

^1^  Department of OBGYN, General Hospital of Nikaia “Agios Panteleimon”, Athens, Greece
^2^  Division of Pediatric and Adolescent Gynecology, 2nd Department of Obstetrics & Gynecology, University of Athens, Athens, Greece

**Introduction and Aims of the Study:** Adolescent pregnancy has been associated with increased maternal and neonatal risks. The aim of this study is to present the characteristics of adolescent pregnancies and deliveries in a general hospital of Athens, Greece.

**Methods:** All deliveries of women up to 18 years old from 16 June 2018 to 15 June 2021 were recorded (n = 55), along with demographic characteristics, mode of delivery, neonatal characteristics, and hemoglobin and platelets levels before delivery and on first postnatal day.

**Results and Discussion:** Median age at delivery was 16 years old (IQR 15–17). Out of 55 adolescents, 52 were single (94.5%), 41 (74.4%) had Greek nationality, three were refugees or asylum seekers (5.5%), 39 (70.9%) were nulliparous, 13 (23.6%) primiparous, and 3 (5.5%) were multiparous. Median gestational age at delivery was 38 completed weeks (IQR 37–39), 11 (20%) adolescents gave birth preterm, and 42 (76.4%) delivered vaginally. Maternal request was the most common recorded reason for caesarean section (38.5%). Median birth weight was 3100 g (IQR 2810–3250), while 9 (16.4%) neonates had low birth weight (<2500 g). Pregnancy anemia (hemoglobin < 11 g/dL) was diagnosed in 23 (41.8%) adolescents, while median hemoglobin before delivery was 11.25 g/dL (IQR 10.05–12.30). Median hemoglobin drop after delivery was 0.90 g/dL (IQR 0.10–1.60), while median platelet drop 18,000/μL (IQR −5000–35,000). When comparing adolescents based on mode of delivery, gestational age was higher in the vaginal delivery group compared to the caesarian section group (39 vs. 38 weeks, non-parametric *p* < 0.01) and hemoglobin drop was less after vaginal delivery compared to caesarian section (0.60 g/dL Vs. 1.90 g/dL, non-parametric *p* < 0.01).

**Conclusions:** Vaginal delivery appears feasible and safe among adolescents with uncomplicated pregnancies. Notably, pregnancy anemia and preterm birth rates are high among adolescent pregnant women.

**P62. Microbial Diversity and Personal Hygiene Patterns in Children with Vulvovaginal Complaints**

**A. Deliveliotou ^1^, O. Toutouza ^2^, P. Apostolakopoulos ^3^, G. Creatsas ^1^**

^1^  Department of Pediatric and Adolescent Gynecology, Rea Maternity Hospital, Athens, Greece
^2^  School of Medicine, Imperial College of London, London, UK
^3^  Department of Microbiology/Biopathology, Rea Maternity Hospital, Athens, Greece

**Introduction:** Vulvovaginitis is one of the most common gynecological problem of childhood. The microbial flora in girls with clinical signs and symptoms of vulvovaginitis is variable. The vaginal microbiome is complex, and the presence of potential pathogens does not necessarily suggest the etiology of vulvovaginal complaints.

**Aim of the Study:** To determine and compare clinical and microbiological features of vulvovaginitis in prepubertal girls. This study involved 125 girls from 2–12 (4.6 ± 2.5) years old. Detailed medical history as well as personal hygiene status was recorded. Clinical examination was followed by vaginal smear, urine culture, and stool analyses at the same time.

**Results:** The most common symptoms among the girls at presentation were vaginal discharge (68%, vulvar erythema (37.8%), and vaginal pruritus (24.4%) and vulvodynia (10%). Microorganisms, isolated from vaginal smears, were detected in 48.9% of the patients. The most common isolated pathogens were opportunistic bacteria of faecal origin. Concurrent Escherichia coli was shown in the urine culture of 13 patients with vulvovaginitis (10%). In microscopic stool analysis parasites with the prominence of Enterobius vermicularis were detected in 50/125 (40%). Regarding personal hygiene patterns, self-cleaning after defecation was the predominant factor associated with positive vaginal culture (90%) followed by wiping back to front (75%), toilet paper use (68%) and wet wipes (32%), and bathing sitting (45%) among girls. A 32% of girls with isolated pathogens reported to wear moreover tight clothing in a daily basis.

**Conclusions:** Our results suggest that vulvovaginitis in prepubertal girls is not only related to microbial pathogens, but to specific personal hygiene patterns as well. Children have commonly poor hygiene in the anogenital region, do not wash their hands frequently and can easily transfer pathogens from the oropharynx to genital area. Detailed counselling to children of all ages and their parents/caregivers about personal hygiene should always be provided, with the perspective of a healthy reproductive life in the future.

**P63. The Association of Educational Level with Contraception Use Among Socioeconomically Deprived Adolescents and Young Adults in Greece: An Extended Analysis**

**G. Kostomoiris ^1^, E. Tsarna ^1,2^, A. Matsas ^2^, E. Paschalidou ^2^, A. Lazaridis ^2^, P. Christopoulos ^2^**

^1^  General Hospital of Nikaia, “Agios Panteleimon”, Department of Obstetrics and Gynecology, Nikaia, Greece
^2^  Division of Adolescent and Pediatric Gynecology, Second Department of Obstetrics and Gynecology, Medical School, National and Kapodistrian University of Athens, Athens, Greece

**Introduction and Aims of the Study:** Lack of education and poverty are associated with low contraception use and unintended pregnancies. The aim of this study was to examine how contraception use is affected by educational level among adolescents and young adults in a socioeconomically deprived population in Greece.

**Methods:** Our study population was all sexually active adolescents and young adults who visited the outpatient OB/GYN offices between 2017 and 2023. Based on self-reported data, Chi-squared test was applied to compare contraceptive choices across hierarchical educational levels.

**Results and Discussion:** Out of 544 women up to 25 years old included in this study, 38 had attended less than 9 years of education, 129 had attended 9 years of education, 131 had attended 12 years of education, 104 had concluded postsecondary education, 133 had a BSc, and three had an MSc/PhD. Only 45 participants (8.3%) reported no use of contraception and were of lower educational level (*p* < 0.01). Notably, 11 out of 38 women (28.9%) who had attended less than 9 years of education reported no use of contraception. With regard to type of contraception used among 499 users, higher educational level was associated with more frequent use of the male condom (*p* < 0.01) and less frequent use of the withdrawal method (*p* < 0.01) and intrauterine devices (*p* < 0.01). Use of oral contraceptives (*p* = 0.60) and emergency hormonal contraception (*p* = 0.58) did not differ across educational levels.

**Conclusions:** Among adolescents and young adults in a socioeconomically deprived area in Greece, higher educational level was associated with contraception use. The ineffective withdrawal method was more popular among participants with lower educational level, while male condom, offering concomitant protection against sexually transmitted diseases, among participants with a higher educational level.

**P64. Greek Doctors’ Perception of Ambiguous Genitalia**

**L. Tzounakou ^1,2^, N. Vlachos ^3^, G. Daskalakis ^4^, L. Michala ^5^**

^1^  Leoni Tzounakou, First Department of Pediatrics, National and Kapodistrian University of Athens, Master of Sciences (MSC) Program General Pediatrics and Pediatric Subspecialties: Clinical Practice and Research”, Children’s Hospital Agia Sophia, Athens, Greece
^2^  Leoni Tzounakou, Second Department of Pediatrics, National and Kapodistrian University of Athens, Children’s Hospital p. and A. Kyriakou, Athens, Greece
^3^  Nikolaos Vlachos MD PhD FACOG Professor and Chair of Second Department of Obstetrics and Gynecology of National and Kapodistrian University of Athens Aretaieio Hospital, Athens, Greece
^4^  George Daskalakis, Professor of First Department of Obstetrics and Gynecology, National and Kapodistrian University of Athens, “Alexandra Hospital”, Athens, Greece
^5^  Lina Michala, Associate Professor of First Department of Obstetrics and Gynecology, National and Kapodistrian University of Athens, “Alexandra Hospital”, Athens, Greece

**Introduction:** During the last decades there is a huge advance regarding the knowledge of the needs of people with ambiguous genitalia as far as the medical part is concerned but more impressively the psychological part. As a result, the worldwide management of people with Differences of Sex Development (DSD) tends to conform to their needs. The aim of this study is to assess the current perceptions of neonatologists and obstetrician-gynecologists about DSD people in Greece.

**Methods:** We used a cross-sectional study in form of poll with a questionnaire. The final sample was 55 answers. At first, we conducted descriptive statistics and then statistical analysis using SPSS.

**Results:** We found that the 38.2% knew the right answer about the new law in the country where prescribing hormones or other medications to children under 15 years old is forbidden just for normalizing reasons.

The results showed that there was statistically significant difference between the gynecologists and the neonatologists who will strongly recommend to a DSD parent to take advice by a psychologist with the percentage of 44% and 76.7% respectively, with a *p*-value = 0.021.

Also, the results showed that there was statistically significant difference between the doctors who work in Attica and the doctors who work in other regions of Greece as far as the nomenclature is concerned with a percentage of 91.7% and 42.1% respectively choosing the name ‘person with ambiguous genitalia’, with a *p*-value = 0.004. The rest non-Attica doctors preferred the name ‘atypical’, ‘intersex’, ‘disorder of sex development’, ‘unknown gender’ and ‘hermaphrodites’, in order of frequency.

**Conclusions:** We conclude that there is great need for the medical community to be better informed about this rare but important issue.

**P65. Potential Aspects of Formation of Positive Attitude Towards Menstruation Among Female Adolescents**

**K. Alieva, N. Kokhreidze**

Almazov National Medical Research Centre, Saint Petersburg, Russia

**Introduction:** Healthy period cycle is the crucial aspect of menstrual health (MH), which is a state of complete physical, mental, and social well-being and not merely the absence of disease or infirmity, in relation to the menstrual cycle.

**Aim:** to assess the role of individual factors of sex education and qualitative characteristics of menstrual function at formation of positive attitude towards menstruation in adolescent girls.

**Methods:** Research was performed from May to December 2022 at Almazov National Medical Research Centre (Saint Petersburg, Russia). We surveyed 224 girls about main aspects of MH and menstrual hygiene (MHG). Questionnaire contained 24 questions, divided into 3 groups: general information, evaluation of menstrual cycle, MHG features.

**Results:** Patients were divided in groups with positive (group 1, n = 173) and negative (group 2, n = 51) attitude to menstruation. Mean age: group 1—15.1 ± 1.6 y.o., group 2—14.8 ± 1.8 y.o. (*p* = 0.307). Mean menarche age: group 1—12.3 ± 1.3 years, group 2—11.8 ± 1.1 years (*p* = 0.023). In group 1 mother was more often the primary source of information about MH (83.2 versus 68.6% respectively), at the same time other sources were more frequently mentioned in group 2 (27.5% versus 13.9% respectively) (χ^2^ = 5.36, *p* = 0.021). Patients of group 2 more often suffered from excessive menstrual flow: group 1–67 (38.7%), group 2–31 (60.8%) (*p* = 0.006). Girls in group 2 had more often difficulties in MH in public places (group 1–9 (5.2%), group 2–12 (23.5%) (*p* = 0.001)) and no possibilities to MH implementation at school (group 1–5 (2.9%), group 2—7 (13.7%) (*p* = 0.002)).

**Conclusions:** According to our study the following factors form positive attitude towards menstruation among girls: later onset of menarche, active involvement of mother at education process and on-time informing about menstruations, formed family culture of menstrual calendar use, moderate menstrual bleeding, absence of problems in MH at school, possibilities to disposal of MH products in public places.

**P66. Ovarian Tissue Cryopreservation in a 14-Year-Old Female Patient to Preserve the Possibility of Fertility with Benign Ovarian Disease with a High Risk of Developing Premature Ovarian Failure**

**A. Regendova, M. Bahnikova, D. Ondrova**

The Institut for the Care of Mother and Child, Prague, Czech Republic

Ovarian tissue cryopreservation (OTC) is one of the methods of preserving fertility in women with a malignant or benign disease in which there is a high risk of premature ovarian insufficiency. OTC is basically the only option for preserving fertility for prepubertal and postpubertal girls who cannot undergo ovarian stimulation or for girls when the start of oncological treatment cannot be delayed. In the following text, we describe our case report of OTC in a 14-year-old girl with benign ovarian disease—recurrent dermoid cyst.

A 14-year-old female patient visited our clinic for the first time in December 2023 with a dermoid cyst on the right ovary. The patient already had left oophorectomy due to ovarian torsion with big dermoid cyst in May 2023. From September 2023, the girl was monitored for a dermoid cyst on the right ovary with growth progression. During her first visit in December 2023, the patient was asymptomatic, her menstrual cycle was regular, secondary sexual characteristics were fully developed. According to the expert ultrasound examination, the right ovary was with cystic-solid resistance of mixed echogenicity, size 66 × 67 × 83 mm, with a smooth surface of the lesion, without pathological perfusion.

The conclusion of the ultrasound examination was a benign dermoid cyst with the presence of residual ovarian tissue measuring 14 × 7 × 13 mm. A secondary ultrasound finding of a congenital developmental defect of the uterus—uterus septus—was also diagnosed. Due to the size of the lesion, laparoscopic enucleation of the dermoid on the right ovary was recommended to the patient. Due to the patient’s burdensome anamnesis—condition after left oophorectomy, recurrent dermoid cysts, the possibility of ovarian tissue collection for cryopreservation was proposed—both the parents and the patient agreed. Before the planned procedure, the tumor markers were negative, the AMH value was lower—1.3 ng/mL. At the beginning of January 2024, a laparoscopic enucleation of the dermoid on the right ovary was performed in the endobag, sufficient residual ovarian tissue was found intraoperatively and a sample was taken for its cryopreservation. The definitive histology revealed a cystic teratoma of the ovary, mostly mature, only in 1 out of 14 blocks was a focally captured microsection of primitive undifferentiated neuroectoderm elements, which corresponded to a low grade immature teratoma of the ovary. At the postoperative check-up one month after the procedure, the right ovary was 25 × 26 × 29 mm in size with several follicles, without the presence of solid or cystic resistance in the area of the right adnexa. The patient was put on hormonal contraception in order to protect the ovarian reserve. Due to the result of the definitive histology, the patient went through our oncology board and was subsequently consulted in the pediatric hematology and oncology clinic. Oncological treatment was not indicated, the solitary ovary was left. The patient’s regular dispensary continues in our pediatric gynecology clinic, and the plan is to add an oncogenetic examination. Her ovarian tissue has been preserved for now, although due to the results of histology, eventual autotransplantation is currently questionable.

**P67. The Time Trend of Age at Menarche in Greece: An Extended Analysis**

**E. Tsarna ^1^, M. Marasioni ^1^, E. Panagodimou ^2^, G. Gkyrti ^2^, N. Vlahos ^2^, P. Christopoulos ^2^**

^1^  Department of OBGYN, General Hospital of Nikaia “Agios Pantaleimon”, Athens, Greece
^2^  Division of Pediatric and Adolescent Gynecology, 2nd Department of OBGYN, Medical School, National and Kapodistrian University of Athens, Athens, Greece

**Introduction and Aims of the Study:** Several environmental factors affect age at menarche, which is estimated to change over time from the prehistoric period to the present. The aim of this study was to describe time trends in age at menarche among women residing in Greece that were born between the 1930s and the 2000s.

**Methods:** All women that visited the outpatient OB/GYN offices between 2017 and 2023 provided structured interviews that included questions regarding the age at menarche and the year of birth. Due to heavily tailed data, the non-parametric Spearman rank correlation coefficient (rs) was calculated. To control for genetic variation and differential environmental exposures, the analysis was also performed within nationality subgroups with more than 50 study participants.

**Results and Discussion:** Our total study population was 3440 women, among which rs was −0.08 (*p* < 0.01), capturing a decline in age at menarche from 13 years among women born in 1930s to 12.1 years among those born in 2000s. Similarly, among women of Greek nationality rs was −0.08 (<0.01, n = 2565), among women of Albanian and Central/Eastern European nationalities rs was −0.25 (*p* < 0.01 with n = 355 and *p* = 0.02 with n = 81 respectively), and finally among women with former USSR nationalities rs was −0.15 (*p* = 0.03, n = 221). The correlation between age at menarche and year of birth was not significant only among women with Middle East and North African nationalities (rs = −0.08, *p* = 0.54, and n = 68).

**Conclusions**: Based on our analysis, age at menarche in Greece declined with time among women born after the 1930s. This decrease was also observed in nationality subgroups. Nonetheless, the magnitude of this decrease differed based on nationality, which may reflect differential environmental exposures across different counties and the effect of migratory status on menarche.

**P68. Genetic and Epigenetic Factors and the Association with the Development of Postpartum Psychosis: A 5-Year Systematic Review**

**S. Tsokkou ^1,2^, M. Georgaki ^1,4^, I. Konstantinidis ^1,3^, A. Keramas ^1,2^, D. Kavvadas ^1,2^, K. Papadopoulou ^1,2^, T. Papamitsou ^1,2^, S. Karachrysafi ^1,2^**

^1^  Research Team “Histologistas”, Interinstitutional Postgraduate Program “Health and Environmental Factors”, Department of Medicine, Faculty of Health Sciences, Aristotle University of Thessaloniki, 54124 Thessaloniki, Greece
^2^  Laboratory of Histology-Embryology, Department of Medicine, Faculty of Health Sciences, Aristotle University of Thessaloniki, 54124 Thessaloniki, Greece
^3^  Interinstitutional Postgraduate Program “Health and Environmental Factors”, Department of Medicine, Faculty of Health Sciences, Aristotle University of Thessaloniki, 54124 Thessaloniki, Greece
^4^  Environmental Engineering Laboratory, Department of Chemical Engineering, Aristotle University of Thessaloniki, 54124 Thessaloniki, Greece

**Purpose:** Postpartum psychosis (PPP) is a serious mental health illness affecting women post-parturition. Around 1 in 1000 women are affected by postpartum psychosis, and the symptoms usually appear within 2 weeks after birth. Postpartum mental disorders are classified into 3 main categories starting from the least to most severe types, including baby blues, postpartum depression, and postpartum psychosis.

**Materials and Methods:** In this systematic review, genetic and epigenetic factors associated with postpartum psychosis are discussed. A PRISMA flow diagram was followed, and the following databases were used as main sources: PubMed, ScienceDirect, and Scopus. Additional information was retrieved from external sources and organizations. The time period for the articles extracted was 5 years.

**Results:** Initially, a total of 2379 articled were found. After the stated criteria were applied, 58 articles were identified along with 20 articles from additional sources, which were then narrowed down to a final total of 29 articles.

**Conclusions:** It can be concluded that there is an association between PPP and genetic and epigenetic risk factors. However, based on the data retrieved and examined, the association was found to be greater for genetic factors. Additionally, the presence of bipolar disorder and disruption of the circadian cycle played a crucial role in the development of PPP (Figure 19).

**P69. Modern Computer Technologies for Wide Coverage and Solution of Reproductive Problems of Adolescents and Young Adults**

**G. Alimbayeva ^1^, G. Aldangarova ^1^, A. Yermyrza ^2^**

^1^  Kazakh-Russian Medical University, Almaty, Kazakhstan
^2^  Gymnasium named after Kanysh Satpayev, Almaty, Kazakhstan

**Introduction:** It is known that AYAs care little about their health, much less show interest in visiting medical institutions and various consultations and examinations. The generations of AYAs is inseparable from gadgets and is accustomed to receiving information from the Internet and trust it.

**The Aim of the Project:** Is to use computer technologies to widely cover AYAs, clarify the presence of PMS symptoms and their severity to motivate visit a specialist.

**Methods:** Based on the Excel program, using a QR code (Figure 20), AYAs are able to anonymously assess their symptoms and make a decision to visit a specialist. Moos’s Menstrual distress Questionnair MDQ, 1969 (Figure 21), consisting of 6 section such as Psycho-emotional disorders, Neurological symptoms, Water and electrolyte imbalance, Gastrointestinal symptoms, Skin or Musculoskeletal manifestations and 48 possible symptoms in them. Symptom severity is assessed independently in points from 1 to 6, with 1 being the absence of a symptom and 6 being the maximum severity that interferes with daily activities. The time required to fill out the questionnaire is no more than 10 min.

**Results:** During the 3 months of the project, 202 people visited the platform. Average filling time was 6 min. More than 70% of respondents recognized this form of interaction as optimal. A severe form of PMS/PMDD was previously confirmed in 27 (13.4%) girls and treatment was started with Vitex Agnus Castus at a daily dosage of 20 mg.

**Conclusions:** The use of computer technology to reach the younger generation opens up broad prospects for medical workers, both in the initial screening of possible problems and in the dynamic monitoring the treatment results. Some changes in the focus of the questions make it possible to assess other gynecological problems.

**P70. Starting from Scratch—Setting Up a Tertiary Paediatric and Adolescent Gynaecology Multi-Disciplinary Team (Pag Mdt) Meeting at Uhbw**

**S. Channing, S. Armstrong, R. Hamblin, B. Strachan, N. Crouch**

St. Michael’s Hospital, Bristol, UK

**Introduction:** A PAG MDT is a requirement of NHS England’s specialised commissioning for the care of patients with congenital gynaecological anomalies (Differences in Sex Development (DSD) and complex Müllerian anomalies) 2019.

At our hospital a DSD MDT meeting already existed for paediatric patients and adolescents, but no equivalent for those transitioned to adult services.

Therefore, a PAG MDT was developed, with the first meeting in November 2021, having been delayed by the COVID-19 pandemic.

**Methods:** The hour-long monthly meeting has PAG clinicians, a PAG psychologist and a gynaecological radiologist as core members, with a planned role for a nurse specialist.

On a case-dependent basis other specialists are invited to attend, including an advanced laparoscopic gynaecologist, endocrinologist and geneticists.

A rolling agenda comprises case discussions, education and research updates. A spreadsheet was devised and contemporaneous notes made and added to the patient electronic records.

Managerial support was sought for time to be allocated in job plans and IT support to develop electronic administration.

**Results:** The meetings have consistently run monthly on both virtual and hybrid platforms.

85 patients have been discussed, some multiple times. 48 of these had a congenital gynaecological anomaly.

**Discussion:** The meeting has extended to create a forum to consider best care for all complex cases and enable regional clinicians to join remotely.

Technical support has evolved from a simple spreadsheet of patients to an online request form and record of discussion held within the patient’s electronic notes. This has streamlined administration and improved visibility of outcomes.

The original vision included showcasing departmental educational/research activities and discussion of PAG service development ideas, however these now need a separate meeting as clinical MDT discussion cases have increased considerably.

**P71. Reconstructive Surgery Following Female Genital Mutilation (FGM)**

**I. Dedes, A. Nikolis, A. Vatopoulou, F. Gkrozou, C. Skentou, M. Paschopoulos**

University of Ioannina, Ioannina, Greece

**Introduction and Aims:** To highlight the challenges of FGNM surgery, to present modern surgical techniques and the factors attributing to surgical complications on restoration of genital anatomy, to improve sexual function and the prognostic factors of surgical complications on women who undergo FGM reconstructing surgery. The introduction of novel surgical techniques for clitoral and vulvovestibular reconstruction. The emphasis on the ability of modern surgical techniques on repairing the female genitalia, restoring women’s natural genital anatomy, allowing to improve female sexuality.

**Methods:** We studied the current literature regarding the prevalence of FGM as well as its consequences. Cohort studies and case studies of women who have undergone FGM reconstruction surgery were reviewed to examine the effectiveness and postoperative outcomes of these surgical procedures

**Results:** According to cohort studies, patients significantly reported postoperative reduction of dysmenorrhea, dysuria, and dyspareunia as well as significant improvement of clitoral sensation and ability to achieve orgasm. Case studies have shown that clitoral reconstruction assisted with psychosexual therapy resulted in improvement in pain during intercourse, sexual function and self-confidence. Improved sexual sensation is due to the remaining neurons prior to the surgery, cases where part of the clitoris remains subcutaneous have been described.

**Conclusions**: FGM reconstructive surgery must be practiced by skilled and familiar with the subject Specialists. Procedures as described contribute in improving the effectiveness, in terms of vulvar pain, dyspareunia, sexual activity and sensuality in the area, and assist in achieving of orgasm, self-image improvement and mental health. Omit FGM victims should be informed about the available surgeries that could benefit them.

**P72. Crossroads Between PAG and Urogynecology**

**V. Zolota, V. Triantafyllidi, S. Athanasiou, D. Zacharakis, G. Daskalakis, L. Michala, T. Grigoriadis**

1st Department of Obstetrics and Gynecology, ‘Alexandra’ General Hospital, National and Kapodistrian University of Athens, 80 Vasilissis Sofias Avenue, 11528 Athens, Greece

**Introduction and Aims:** Adolescent girls born with complex Urogenital anomalies require specialized treatment when transitioning into adulthood. Despite successful reconstructive surgery in childhood and adolescence, certain gynecological and urological symptoms may remain, requiring assessment and management in adulthood. Our aim is to present the urodynamic assessment of two girls with bladder exstrophy and cloacal malformation respectively.

**Methods:** Two Case Presentations.

**Results:** The first patient is a 23 years old girl born with bladder exstrophy. She uderwent bladder ileal augmentation with orthotopic urethral opening in adolescence. She later presented with filling phase urodynamic incontinence (above 250 mL) at 17 years old. She also had a Fenton’s procedure at age 21 due to vaginal stenosis, which allowed her to have penetrative sexual intercourse. Τhe current urodynamic study showed severe urodynamic stress incontinence. Her treatment options have been discussed in a Multidisciplinary team and she will undergo urethral closure and Mitrofanoff procedure. The second patient is a 19 years old girl with cloacal malformation. She was operated in neonatal age. Initially, a colostostomy was created, followed by a posterior sagittal anorectoplasty (PSARP). Moreover, she underwent surgical repair of an esophageal atresia and annular pancreas as a child. At age of 15 years old she had a second PSARP due to persistent rectovagina fistula and reconstruction of the perineal body. She also had an ileostomy with a Malone appendicostomy to facilitate defecation. Soon after the second PSARP she presented with urinary retention, for which she had to perform clean intermittent self-catheterization. A urodynamic study performed at age of 19 showed normal bladder sensation, and ability to urinate. Four years following PSARP she is now able to urinate spontaneously.

**Conclusions:** A multidisciplinary approach including urogynecology specialists may assist the management of women, born with complex urogenital anomalies and persistent pelvic floor disorders.

**P73. Sleep in Adolescents**

**S. Mili ^1^, L. Michala ^2^**

^1^  Athens College, Hellenic-American Educational Foundation, Athens, Greece
^2^  National and Kapodistrian University of Athens, Athens, Greece

**Introduction:** Sleep is important for the health and development of adolescents. Healthy sleep habits enhance memory, attention and learning abilities and, as such, improve the academic performance of the adolescent.

**Aim:** The aim of this project was to create an Instagram page that would provide important information regarding teenage sleep, along with results of a survey on how adolescents sleep.

**Methods:** A literature search was performed, aiming at identifying guidelines regarding sleep in adolescence, from scientific societies.

A questionnaire was created to assess sleep patterns, habits and sleep disturbances among adolescents. The Athens Insomnia Scale was used, along with questions, assessing the use of mobile phone and screen time during the day and before and during sleep. The questionnaire was anonymous and was transcribed on google forms and disseminated to adolescents. Simple demographic data were also collected.

**Results:** Sixty-six adolescents responded to the questionnaire. 25.8% were boys and the median age of respondents was 15 (range 13–18). 60% felt that their sleep was inadequate and as a result, 68% felt they were sleepy during the day. 69% of participants slept with their smartphone next to their bed and 79% scrolled through social media platforms before falling asleep.

The results of the survey, along with information obtained during the research phase of the project were conveyed in a public Instagram page called teens.healthy.sleep.

Followers engaged with the page, asking questions and making comments, which were answered by the owner.

**Conclusions:** Adolescents often have disturbed sleep patterns, which may be secondary to their excessive use of smartphones, especially prior to their bed time. Screen time adversely affected sleep, leading to insomnia. As a result, many adolescents felt tired during the day.

Informing teens regarding the significance of sleep is important and through this Instagram page we hope to convey a positive message to our followers.

**P74. Large Adnexal Masses in Pag Patients-One Center Case Series**

**D. Ivanova Panova ^1^, I. Aluloski ^1^, A. Buklioska ^1^, A. Sima ^1^, V. Jovanovska ^1^, A. Atanasova Boshku ^1^, Z. Stankovic ^2^**

^1^  University Clinic for Obstetrics and Gynecology, University Ss Cyril and Methodius, Skopje, North Macedonia
^2^  FIGIJ Training Center, Belgrade, Serbia

**Introduction:** We present our case series (Table 7) of large adnexal masses in PAG patients which were diagnosed and treated at the University Clinic for Obstetrics and Gynecology in Skopje, Republic of North Macedonia during a period of six months.

Intraoperative images of the patient with serous cystadenoma which was filling the entire abdominal cavity (Figure 22 and Figure 23).

**Conclusions:** All patients had present OCS, which showed as a strong predictor of benign pathology.

Although they had large adnexal mass, all cases were treated with maximal sparing techniques-to preserve the normal ovarian tissue for future fertility.

Postoperatively, they were evaluated and had normal findings.

**P75. Endometriosis in the Adolescent Patient**

**A. Matonóg, A. Drosdzol-Cop, K. Kowalczyk, R. Peterek, A. Leziak**

Department of Gynecology, Obstetrics and Oncological Gynecology, Faculty of Health Sciences in Katowice, Medical University of Silesia, Katowice, Poland

**Introduction and Aim of the Study:** Endometriosis is a chronic disease of the female reproductive organs, which is associated with inflammation, pelvic pain, dysmenorrhea, dyspareunia, and decreased fertility. Among adolescents, endometriosis is the main cause of secondary dysmenorrhea. Manifestations in girls may differ from adults, as abdominal discomfort, lower back pain, heavy menstrual bleeding, dysuria, dizziness, and mood disorders are also observed. The prevalence, according to various sources, ranges from 1.9% to 10–18% of women of reproductive age, while estimates among adolescent girls are similar.

**Method:** A 15-year-old patient suffering from chronic, cyclical lower abdominal pain unresponsive to NSAIDs and significant discomfort significantly impairing her daily activities during menstruation was referred to the Pediatric and Adolescent Gynecology Department of the Bonifraters Hospital in Katowice.

**Results:** The presence of an ambiguous cyst on ultrasonography and high CA-125 marker values (700 U/mL) could indicate a gynecological tumor. In diagnostic laparoscopy, adhesions between the ovaries and fallopian tubes, as well as adhesions between the fallopian tubes and the pelvic peritoneum and omentum, were released (ASRM III, #ENZIAN P1 O0/0 T2/2 A0 B1/1 C0 FI). Atypical endometriosis images included “powder burns” on the intestines. Biopsies of endometriotic tissue and fluid from the Douglas pouch indicated features of atypia. The peri- and postoperative course was uncomplicated. Dienogest use was recommended for 6 months, followed by a repeat laparoscopy with biopsy (ASRM II, #ENZIAN P1 O0/0 T1/1 A0 B0/0 C0).

**Conclusions:** The time from the onset of symptoms to a correct diagnosis typically ranges from 8 to 12 years, underscoring the need for special attention in the diagnostic process due to the risk of decreased quality of life. Despite the current lack of recommendations for CA-125 marker use, its analysis should be considered when evaluating endometriosis in adolescents.


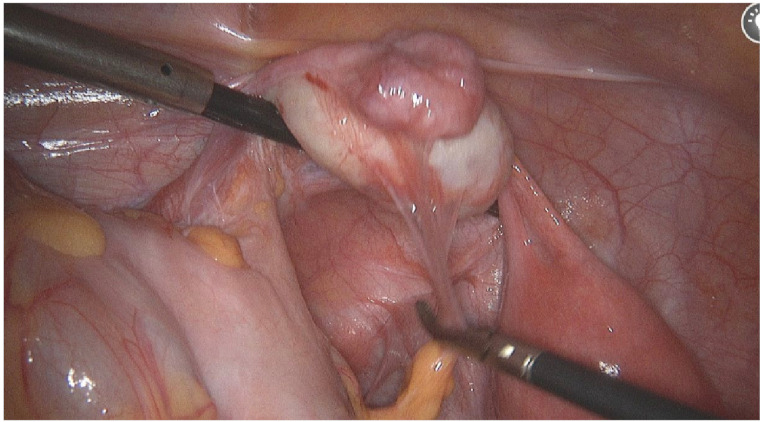



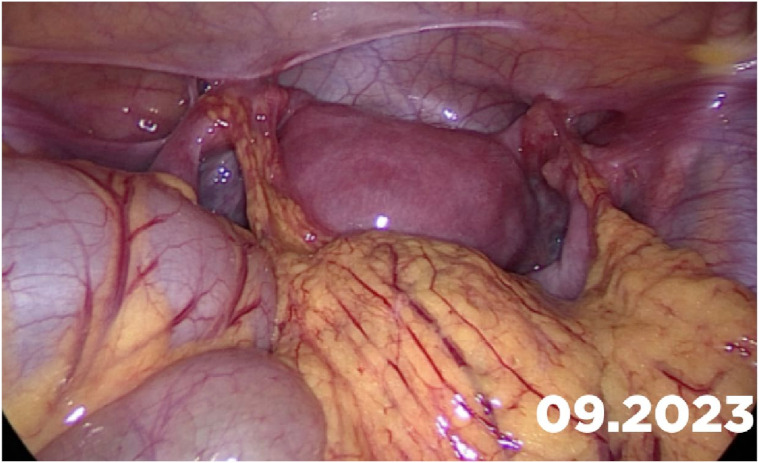

**P76. Contraceptive Methods in Adolescents with Congenital Heart Disease**

**K. Katsikas, E. Mytari, F. Gkrozou, H. Skentou, A. Vatopoulou**

University of Ioannina, Ioannina, Greece

**Introduction:** The enhanced longevity and quality of life in females with congenital heart disease (CHD) increase the probability of adolescents seeking gynecologic care, often regarding available contraceptive options.

**Methods:** After examining existing literature on commonly administered contraceptive methods in adolescents with CHD and assessing documented contradictions along with potential side effects, we compared their safety and efficacy within this patient group.

**Results:**

- Combined Hormonal Contraception (CHC) is associated with an increased risk of atherothrombotic events and thus is contraindicated in patients with related history. Furthermore, when complicated valvular disease is present, CHCs are classified as USMEC category 4 and must be avoided.
- Progestin-only contraceptives are classified as Class 1 medication for women with CHD, with pills specifically constituting an effective alternative, when used correctly. However, lack of adherence leads to significant failure rates resulting in other methods being preferred when pregnancy is contraindicated.
- Progestin implants are preferably used in adolescents with CHD thanks to their high safety and efficacy. Compared to IUDs, they are more likely to cause systemic adverse effects.
- Levonorgestrel Intrauterine Devices (LNG-IUDs) are considered as the primary choice of therapy in adolescents with CHD, with efficacy comparable to sterilization. When administered, they alleviate period related symptoms and prevent excessive bleeding in patients on anticoagulation therapy. Latest guidelines indicate that there is no need for antibiotic prophylaxis.
- Copper IUDs remain the most effective method of emergency contraception, although they may not be optimal for patients receiving anticoagulants or in whom anemia could deteriorate their cardiac status.

**Conclusions:** Concerning young women with CHD, emphasis should be placed on addressing the reported lack of knowledge regarding how sexual activity and/or potential pregnancy could affect their underlying condition. Moreover, education on appropriate contraception selection, considering possible side effects, is considered equally important.

**P77. Serum Angiopoietin-like Protein 8/Betatrophin Levels in Adolescents with Pcos: A Case-Control Study**

**A. Giannouli ^1^, A. Beta ^2^, P. Sidiropoulou ^3^, C. Stefanaki ^1^, S. Markantonis ^3^, F. Bacopoulou ^1^**

^1^  Center for Adolescent Medicine and UNESCO Chair in Adolescent Health Care, First Department of Pediatrics, Medical School, National and Kapodistrian University of Athens, Aghia Sophia Children’s Hospital, Athens, Greece
^2^  Department of Obstetrics and Gynecology, Alexandra Hospital, Athens, Greece
^3^  Laboratory of Biopharmaceutics and Pharmacokinetics, Department of Pharmaceutical Technology, Faculty of Pharmacy, National and Kapodistrian University of Athens, Athens, Greece

**Context:** Adolescents with polycystic ovary syndrome (PCOS) share the similar adverse metabolic profile with adult women with the syndrome. Angiopoietin-like protein 8 (ANGPTL8)/betatrophin is expressed in the liver and fat tissue and has been reported to regulate lipid and insulin metabolism. The role of ANGPTL8/betatrophin in PCOS is yet unclear.

**Objective:** To assess serum ANGPTL8/betatrophin concentrations in adolescents with PCOS, as well as potential correlations with anthropomentric, hormonal and metabolic parameters.

**Patients:** Adolescents, at least two years post menarche, were diagnosed with PCOS if they met the Rotterdam criteria. Adolescents with conditions mimicking PCOS, other chronic diseases or chronic medication use, were excluded for the study.

**Methods:** In this case–control study, adolescents’ anthropometric data were recorded. Commercial methods were used for biochemical and hormonal measurements. ANGPTL8/betatrophin was evaluated with ELISA. Insulin resistance was assessed with the homeostatic model assessment for insulin resistance (HOMA-IR).

**Results:** Thirty-seven adolescents with PCOS and 33 age-matched controls were included in the study. Body mass index (BMI) was higher in adolescents with PCOS than in controls (25.66 ± 5.30 vs. 23.23 ± 4.19 kg/m^2^, *p* = 0.032). ANGPTL8/betatrophin levels did not differ between adolescents with PCOS and controls (*p* = 0.200), and this finding did not alter when data were stratified by BMI or age. Only in the PCOS group, adolescents with insulin resistance (IR) had higher ANGPTL8/betatrophin levels than patients without IR. Positive correlations were found between ANGPTL8/betatrophin and age (r = 0.322, *p* = 0.007) and between ANGPTL8/betatrophin and testosterone (r = 0.354, *p* = 0.004).

**Conclusions:** This is the first study to assess ANGPTL8/betatrophin levels in adolescents with PCOS. The hypothesis of elevated ANGPTL8/betatrophin levels in patients with PCOS than controls, was not confirmed in this age group.

**P78. Non-Communicating Uterine Horns: Our 5-Year Experience**

**K. Koukoubanis, L. Vogiatzi-Vokotopoulou, M. Tsiriva, M. Panagiotopoulos, N. Kathopooulis, G. Daskalakis, A. Protopapas, L. Michala**

National And Kapodistrian University of Athens,1st Department of Obstetrics and Gynecology, ‘Alexandra’ General Hospital, Athens, Greece

**Introduction:** The presence of a non-communicating uterine horn is a rare Müllerian congenital malformation of the female genital tract with an incidence of less than 0.1% of the general population. It is associated with varying clinical presentations depending on the presence of functional endometrium or not, requiring surgical resection. Patients with functional endometrium present with a variety of symptoms such as dysmenorrhea, and chronic or acute abdominal pain.

**Aim—Methods:** This presentation aims to testify the experience of a referral center for the treatment of 6 cases with a non-communicating uterine horn. For that reason, a retrospective case series study was made.

**Results:** From January 2018 to December 2023 in the Department of Pediatric and Adolescent Gynecology, 6 patients were referred. The average age of the patients was 13 years old (range 12–15) and all were referred from their pediatricians. Five out of six patients initially complained of dysmenorrhea and one presented with cyclical pelvic pain and primary amenorrhea. Dysmenorrhoea was so severe in two out of six patients, leading to hospitalization for pain management. All patients had an MRI preoperatively, to map the anomaly in detail. They also had a renal ultrasound to rule out associated renal anomalies, which revealed two ipsilateral renal ageneses. All girls were managed with laparoscopic resection of the rudimentary horn and associated endometriosis was identified in two patients, whereas adenomyosis of the resected horn was found in another two. The two conditions did not coincide.

**Discussion:** Accurate diagnosis of müllerian abnormalities is essential for appropriate management and prevention of complications. However, diagnosis is often missed or delayed because these abnormalities are rare and many pediatric specialists are not fully familiar with them. A diagnosis should be suspected in all girls with early onset and severe dysmenorrhoea.

## Figures and Tables

**Figure 1:**
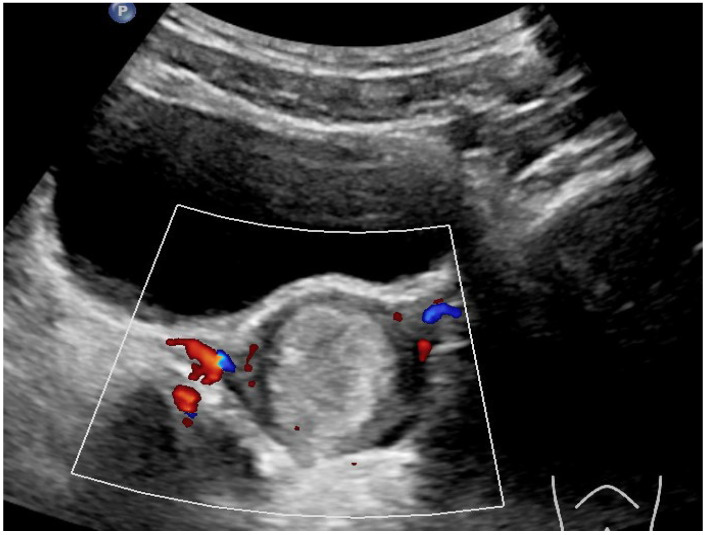
Abdominal ultrasound.

**Figure 2:**
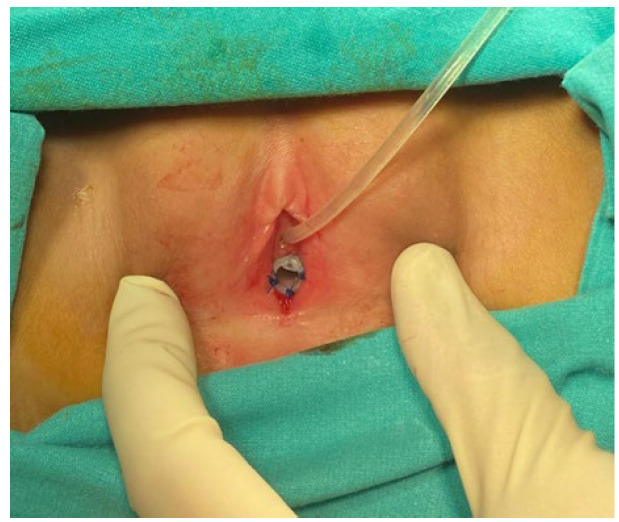
A nonlatex 22 French foley catheter placed in the vagina.

**Figure 3:**
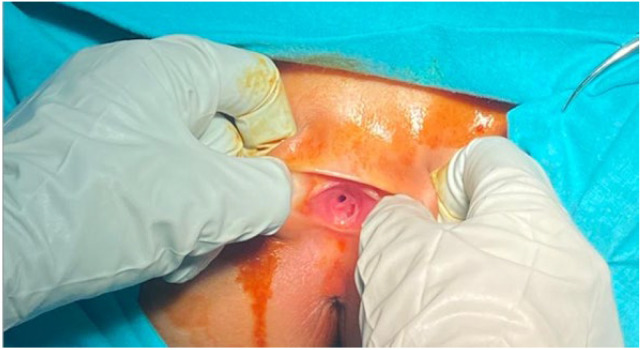
Placement of the stent after 3 months.

**Figure 4:**
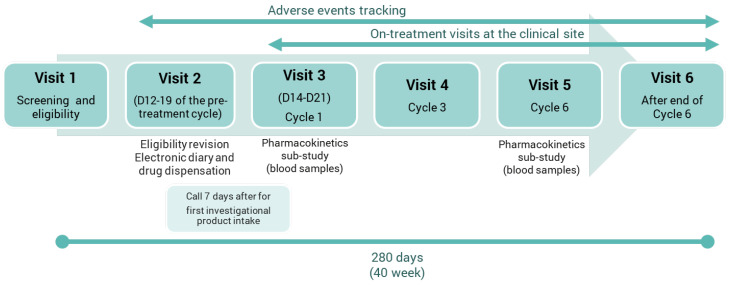
Summary of the study design.

**Figure 5:**
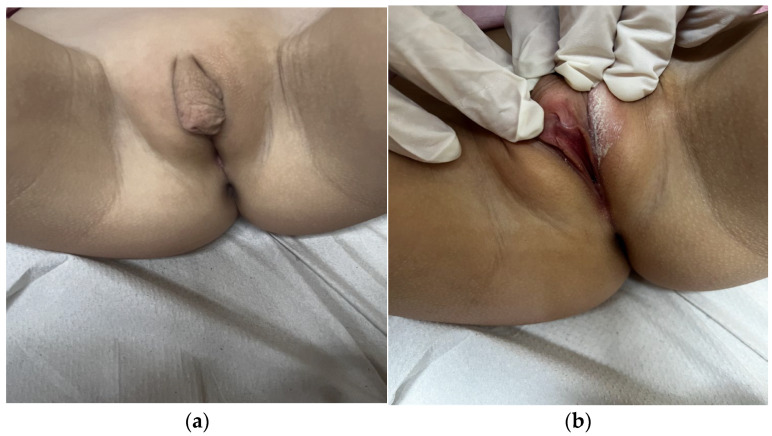
(**a**). External genitalia. (**b**) A normal clitoris after pulling the excess prepuce skin.

**Figure 6:**
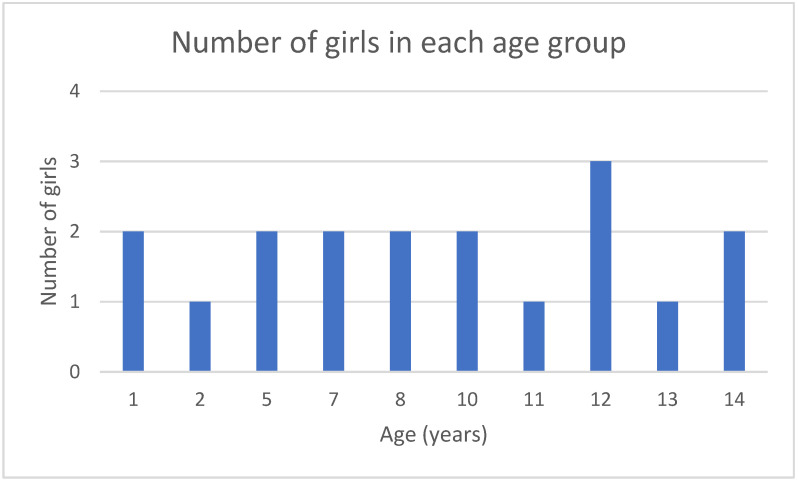
The number of girls in each age group is shown.

**Figure 7:**
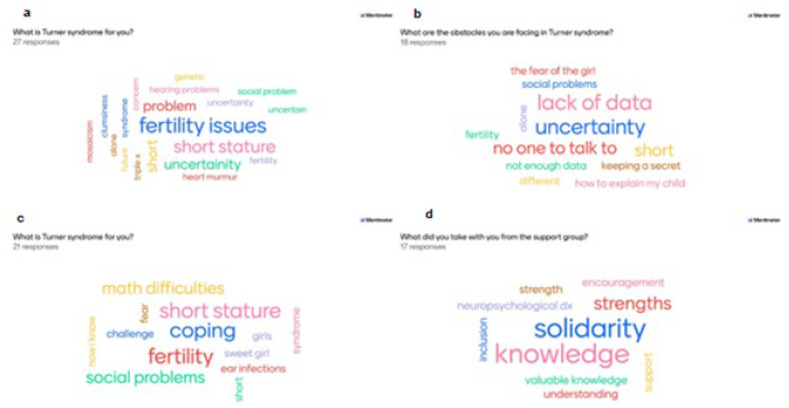
(**a**) Word clouds to the question “What is TS for you?” during the first meeting, (**b**) Word clouds to the question “What are the obstacles you are facing in TS” during the first meeting, (**c**) Word clouds to the question “What is TS for you?” during the last meeting, (**d**) Word clouds to the question “What did you take with you from the support group?” during the last meeting.

**Figure 8:**
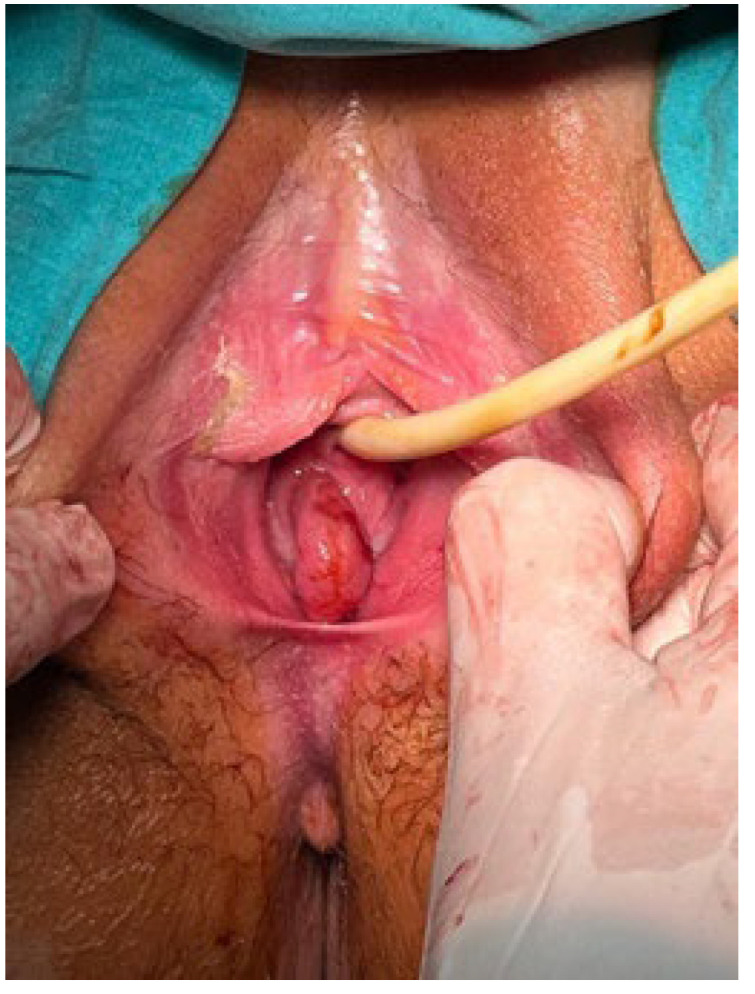
Large polyp-like mass protruding from the vaginal introitus and otherwise normal external genitalia.

**Figure 9:**
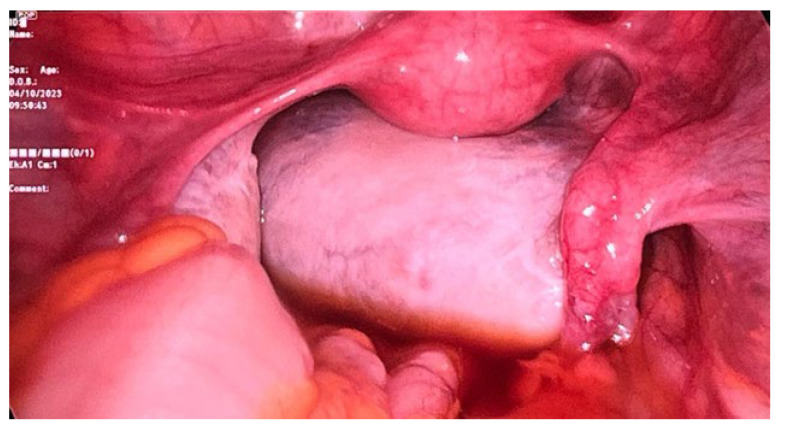
Laparoscopic right salpingo-oophorectomy.

**Figure 10:**
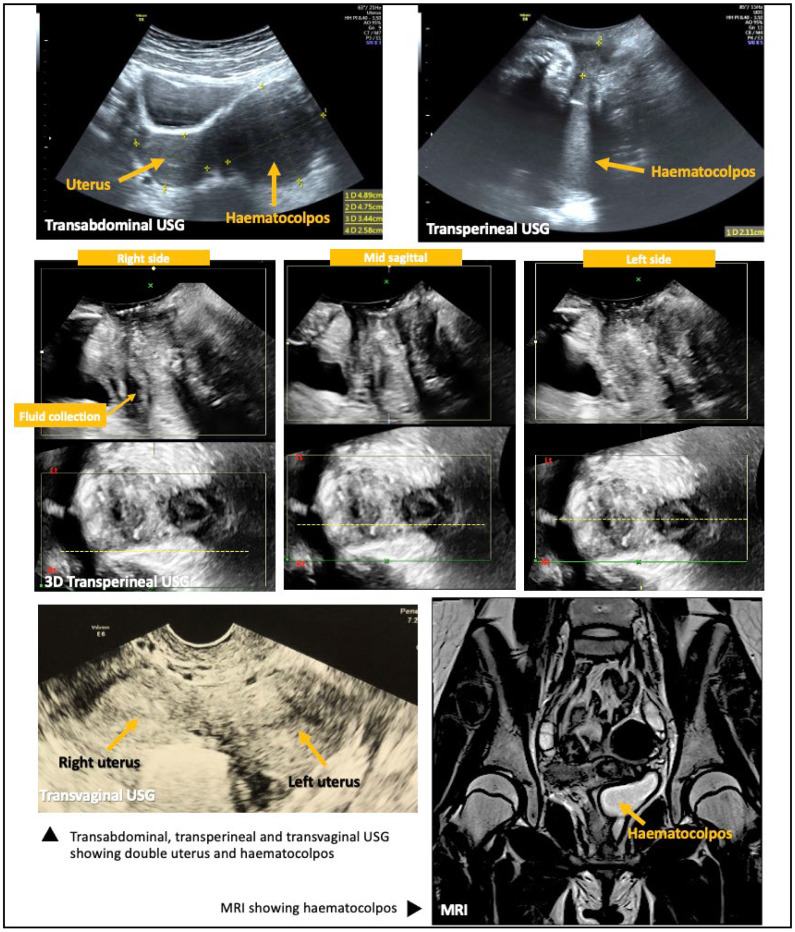
Ultrasound and MRI of cases with OHVIRA.

**Figure 11:**
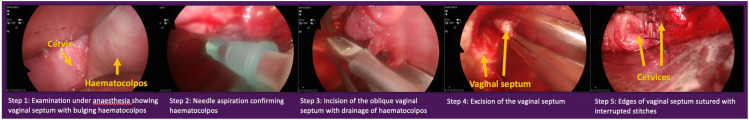
Septum resection for OHVIRA.

**Figure 12:**
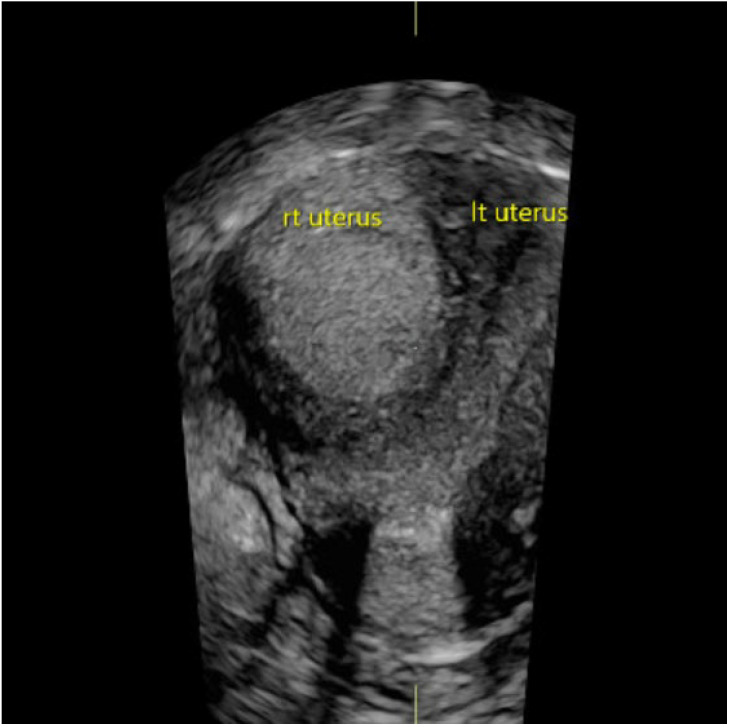
Ultrasound examination.

**Figure 13:**
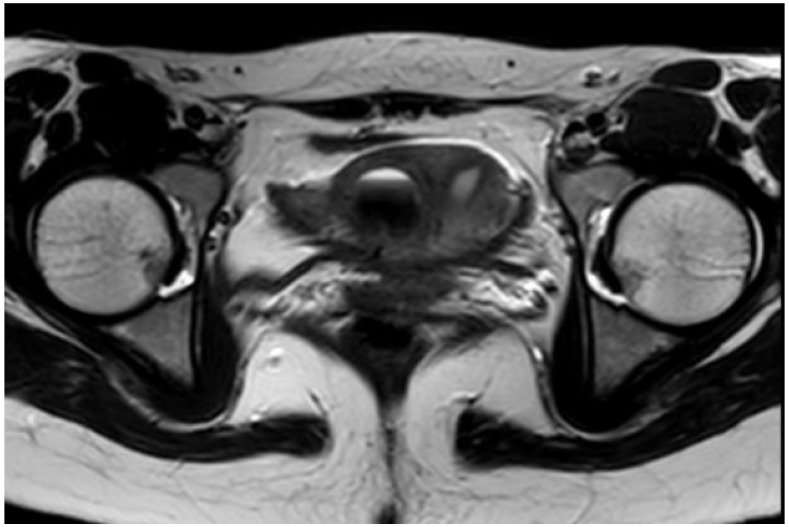
MRI.

**Figure 14:**
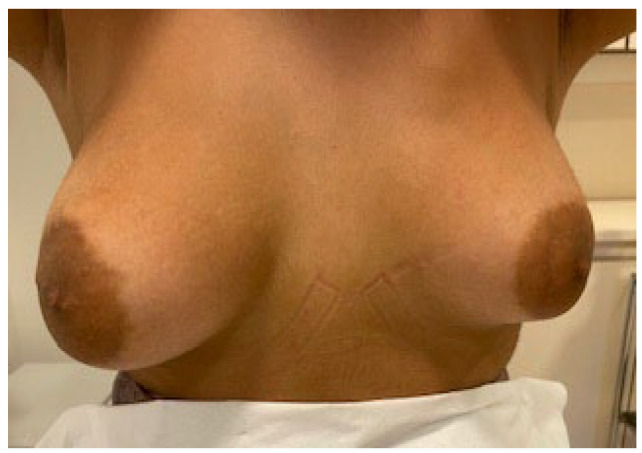
Breast Asymmetry.

**Figure 15:**
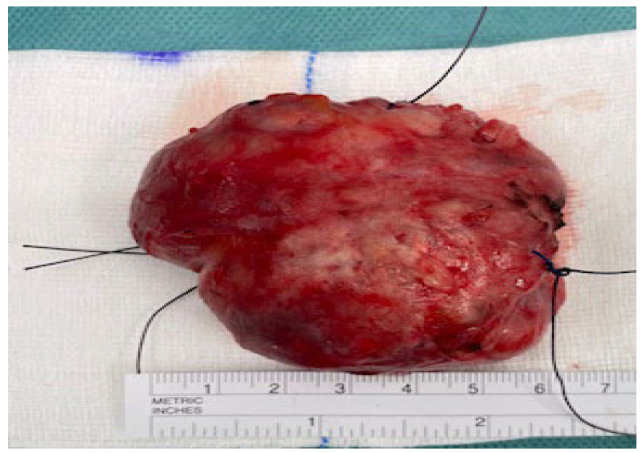
Excised fibroadenoma specimen.

**Figure 16:**
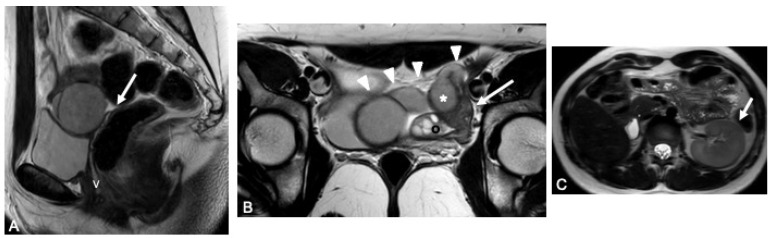
**14-year-old teenage girl with primary amenorrhea, pelvic pain.** (**A**) Sagittal Τ2W MR image of the pelvis demonstrates absence of a normal uterus and cervix (arrow). In the expected location of the vaginal canal (v) a thin fatty band is observed. (**Β**) Axial oblique Τ2W MR image of the pelvis in the same patient shows a left uterine remnant (arrow). A distended with hemorrhagic fluid endometrial cavity (asterisk) and associated hematosalpinx (arrowheads) are observed, indicative of the presence of functional endometrium. Closely associated normal volume ipsilateral ovary (o). (**C**) Axial Τ2W MR image of the upper abdomen in the same patient shows absence of the right kidney. Normal renal location and hypertrophy is shown on the left (arrow). Findings are consistent with Müllerian agenesis with unilateral uterine remnant with functional endometrium.

**Figure 17:**
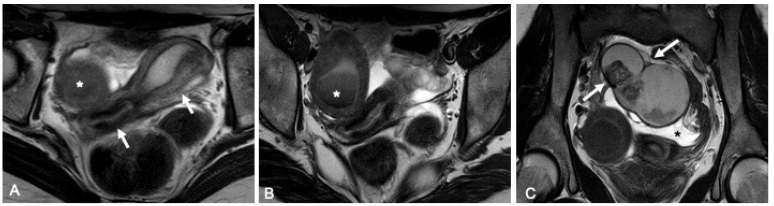
**18-year-old teenage girl with pelvic pain.** (**A**) Axial oblique Τ2W MR image of the pelvis shows a left banana-shaped hemiuterus and single cervix (arrows). There is a closely associated right blind rudimentary horn (asterisk). (**Β**) Axial oblique Τ2W MR image of the pelvis at an upper level demonstrates the right rudimentary horn distended with menstrual fluid (asterisk). (**C**) Coronal oblique Τ2W MR image of the pelvis in the same patient shows associated right hematosalpinx (arrows) and free pelvic fluid (asterisk). Findings are consistent with left unicornuate uterus with right associated non-communicating uterine remnant with functional endometrium.

**Figure 18:**
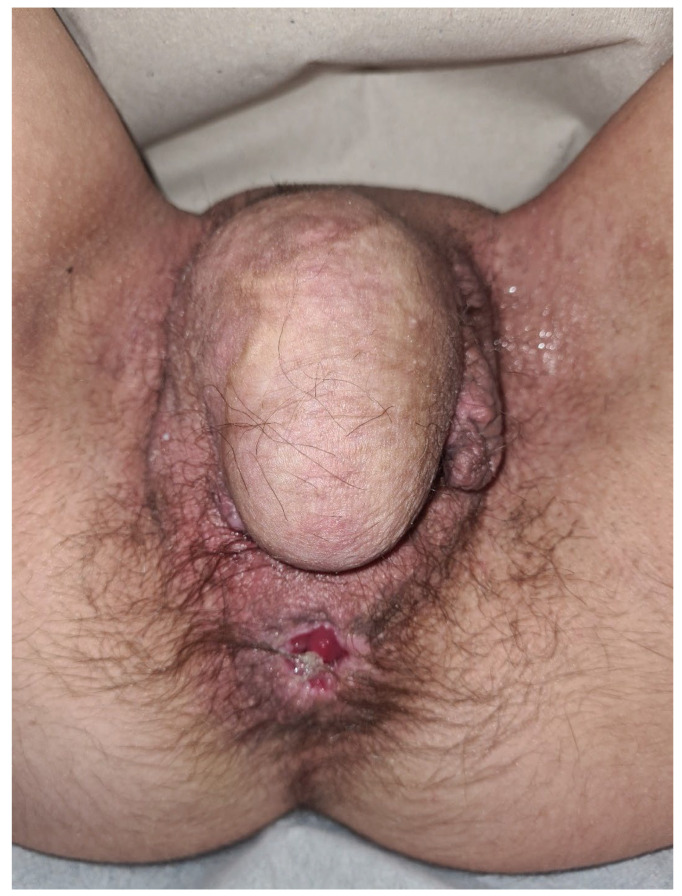
A 20-years-old girl with painful and palpable mass in her vulva.

**Figure 19:**
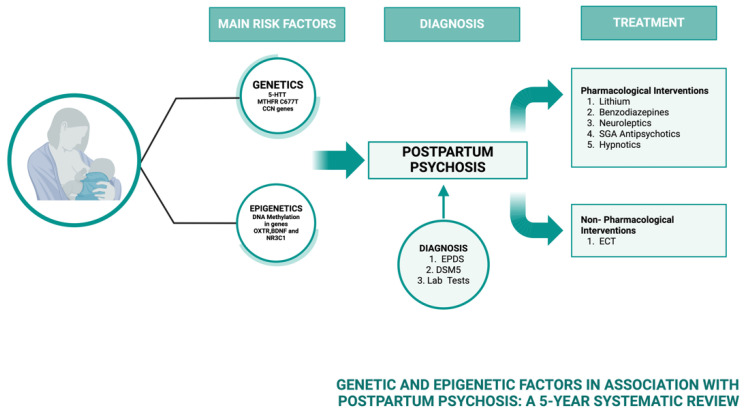
Created with BioRender.com.

**Figure 20:**
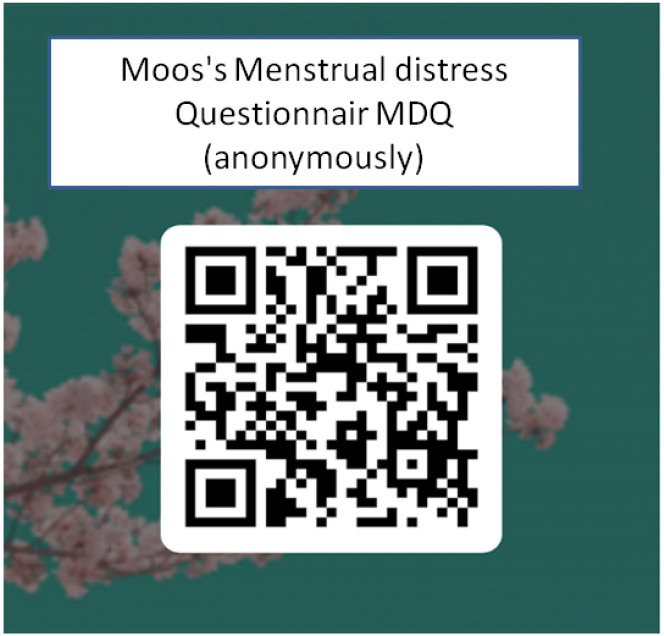
QR code.

**Figure 21:**
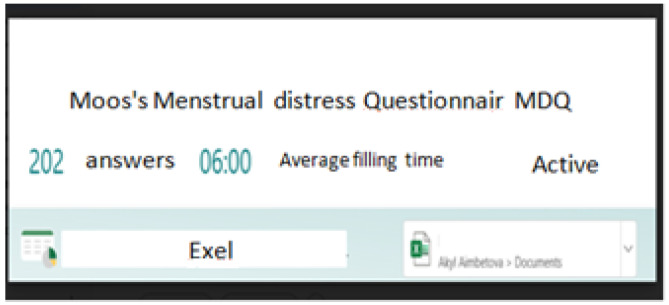
Moos’s Menstrual distress Questionnair.

**Figure 22:**
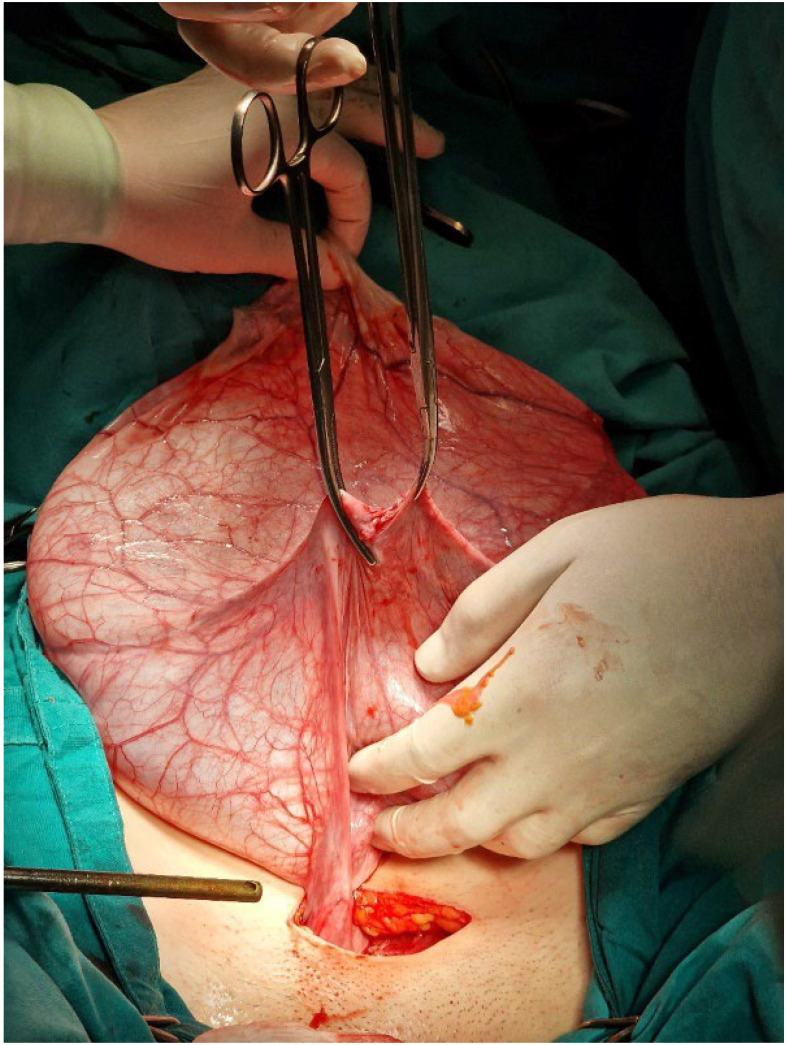
Intraoperative image of the patient with serous cystadenoma.

**Figure 23:**
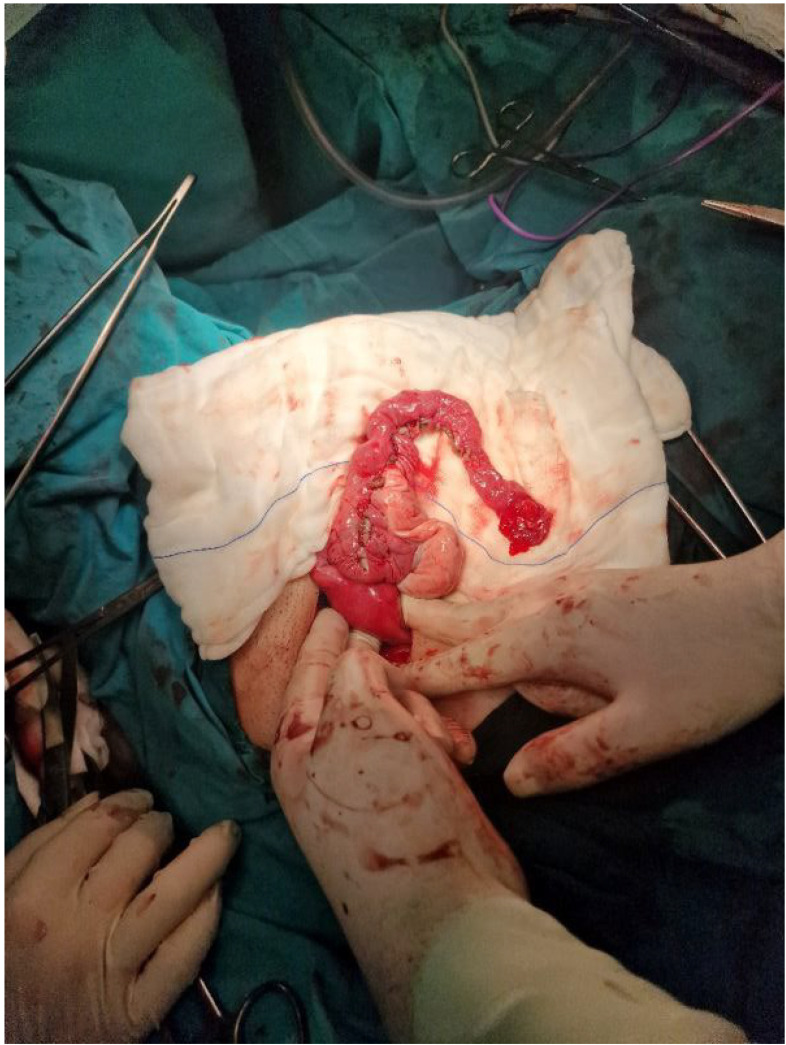
Intraoperative image of the patient with serous cystadenoma.

**Table 1:** Surgical findings and follow-up.

Intraoperative cyst content spillage, n (%)	24 (75)
Postoperative chemical peritonitis, n (%)	None
The median follow up time (months)	35.3 ± 4.3
The patients received postoperative imaging, n (%)	26 (81)
ODC recurrence followed expectantly and with surgery, n (%)	1 (3) and 1 (3)

**Table 2:** Number of relapses reported.

Number of Relapse (s)	1	2	3	4	5
Number of cases (%) Total = 75 (100)	54 (72.0%)	10 (13%)	8 (11%)	2 (2.6%)	1 (1.3%)

**Table 3:** The general characteristics of the patients.

The mean age at the onset of the symptoms(years)	16.2 ± 1.3
Body mass index (BMI)	19.1 ± 2.1
Duration of amenorrhea(months)	10 ± 4.5
Duration of disrupted eating behaviour(months)	8 ± 5.1
DXA lumbar spine Z-score	−1.1 ± 0.7

**Table 4:** CGM Metrics Related to LMP Timing.

CGM Metric	% <54 mg/dL (SD)	% <70 mg/dL (SD)	% 70–180 mg/dL (SD)	% >80 mg/dL (SD)	% >250 mg/dL (SD)	GMI	Coefficient of Variation
7d BEFORE LMP	0.3% (±0.64)	1.5% (±2.3)	48.7% (±23.4)	49.6% (±23.9)	25.4% (±22.4)	8.0% (±1.2)	34.5% (±8.0)
7d AFTER LMP	0.3% (±0.61)	1.5% (±2.2)	49.6 (±22.9)	48.7% (±23.4)	24.5 (±22.4)	8.0% (±1.2)	34.8 (±7.9)

**Table 5:** General characteristics of the patients.

Age	13.7 ± 2.2 years
Duration of continuous COC regime	72.6 ± 81.5 days
Hemoglobin value	10.8 ± 2.2 g/dL
Endometrial thickness	11.43 ± 9.8 mm

**Table 6:** VLS patients in the cohort.

Pathology	Prevalence in VLS Cohort	N	Prevalence in USA Population < 18 y	*p* < 0.05
Eczema	30.77%	12	12.60%	*
Asthma	28.21%	11	6.50%	*
Mood disorders	23.08%	9	18.30%	
Vitiligo	10.26%	4	2.16%	*
Morphea	5.13%	2	0.03%	*
Hashimoto thyroiditis	5.13%	2	3%	
Celiac	2.56%	1	0.33%	*

* statistically siginificant.

**Table 7:** Six patients with large ovarian masses.

Age (Years Old)	11	19	17	18	13	14
**Menarche (years old)**	//	9	12	12	11	10
**Menstrual rhythm**	//	35–40/7	28/7	30/7	28/5	irregular
**Ultrasound measures**	Left 108 × 73 mm	Right-85 mm, left 40 mm	Unmeasurable- filling the abdomen till xyphoid	Left 90 × 78 mm, right 128 × 75 mm	125 × 117 mm on left ovary	155 × 95 mm on left ovary
**Consistency**	clear	Dense billateraly	Clear	Dense billateraly	Mucinous, multichamber	Clear with solid components
**septa**	+	-	-	+	+	+
**OCS**	+	+	+	+	+	+
**Serum tumour markers**	CA125 = 36.7 CA19–9 = 189.05	CA125 = 1538; CA19–9 = 206.9	In range	In range	In range	In range
**Operation**	Laparotomia-extirpatio tumoris ovarii lat sin.	LPSC-extyrpatio capsulae cystae ovarii billateralis	Laparotomia -Extyrpatio cystae ovarii lat dex	LPSC-extyrpatio capsulae cystae ovarii lat. Dex. Resectio ovarii lat sin.	LPSC-extyrpatio capsulae cystae ovarii lat. sin	Laparotomia Extyrpatio Tu cysticum ovarii lat. Sin.
**Pathohistology**	Teratoma maturum ovarii	Endometriosis ovariorum	Cystadenoma serosum	Cystadenoma Ovariorum seromucinosum	Cystadenoma mucinosum	Cystadenofibroma ovarii

## Appendix A

Iakovidis, P	O50	Karachrysafi, S	P68
**A**			
Abdul Ghani, N	P17, P31, P38	Anizah, A	P38
Abdul Ghani, N	O65	Anthoulakis, C	O39, P48
Abu Ishak, N	P17, P30, P31, P38	Anthoulakis, C	O36
Abu Ishak, N	O45	Antoniak, B	P05
Adner Ferrari, D	P08	Antsaklis, P	P32
Ahmad Mizam, I	O45	Apostolakopoulos, P	P62
Akhan, S	O11, O25, O34, O68	Apter, D	O28
Akhan, S	O60	Ardelean, M	O03
Aksenova, T	P60	Armstrong, S	P70
Albana, J	P22	Arnaud, A	O07, O12
Aldangarova, G	P69	Asanidze, E	O15
Alexakis, C	P51	Asaturova, A	O06, O21, P60
Ali, A	O65, P31	Aslanger, A	O60
Ali, A	P17	Atanasova Boshku, A	P74
Alias, H	P38	Athanasiadis, A	O44
Alieva, K	P65	Athanasiadis, A	P37
Alimbayeva, G	P69	Athanasiou, S	P72
Al-Tikriti, S	P15	Avgerinopoulou, E	O32
Aluloski, I	P74	Avnon Ziv, C	O41
Anagnostaki, I	P51	Azhary, J	O45
Andreou, S	P18, P19	Azmirrulnizam, N	P30
**B**			
Bachynska, I	O05, O24	Black, A	O29, O63
Bacopoulou, F	P77	Bonnin, Y	O07
Bahnikova, M	P66	Bouchard, C	O29
Bais, V	P33	Bouchard, C	O63
Balafoutas, D	O14, P61	Bourezma, M	O07
Barel, O	O42	Bourg, A	O07
Barmparousi, A	P51	Bourgioti, C	P39, P40
Bar-On, S	P29, P34	Bousquet, M	O07
Barwinska, J	O67	Brain, P	O01
Baş, F	O60	Brandtner, G	O03
Battle, A	O04	Brodowska, J	O49
Batyrova, Z	O06, O21, P60	Browne, D	P15
Bauer, L	O03	Bruni, V	O40
Belgacem, A	O07	Brus-Chojnicka, A	P12
Ben-Harush Negari, S	O41	Buklioska, A	P74
Benish, M	P29, P34	Bumbuliene, Z	O61
Bernassola, M	P44	Burger, N	O02
Beta, A	P77	Burger, N	O16
BILI, E	O39, P50	Burger, N	P22
Bizzarri, C	O22		
**C**			
Caragheorgheopol, A	P27	Chatzistamatiou, K	P48
Carducci, C	O22	Chen, M	O29, O63
Carrasco Catena, A	P26	Chojnicki, M	P12
Carroll, S	O58	Chondros, S	P51
Cartault, A	O07, O12	Christopoulos, P	O14, O35, P39, P40, P46, P53, P54, P55, P56, P57, P58, P59, P61, P63, P67
Chalkia, K	P18, P19	Chrysafopoulou, E	P51
Chan, S	P07	Chuprynin, V	O06, O21, P60
Chan, S	P36	Clermidi, P	O07
Chan, S	P09	Coello Cahuao, E	P28
Channing, S	P70	Creatsas, G	P62
Charitos, T	P51	Crofts, V	P14
Charmandari, E	P52	Crouch, N	P70
Chatel, G	O28		
**Č**			
Čekuolis, A	O61		
**D**			
Dabeski, D	O20	Deliveliotou, A	O10, P62
Dafopoulos, K	P46	Dembowska-Bagińska, B	P05
Dafoulis, V	O50	Demirsoy, U	O25
Dascalakis, G	O70	Derksen, M	O16
Daskalakis, G	O26, O32, O46, P32, P64, P78	Desantes Real, D	P26
Daskalakis, G	P52	Di Paolo, G	P44
Daskalakis, G	P11, P72	Dimakakou, K	O32
De Leeuw, R	P22	Dinas, K	P37
De Leeuw, R	O16	Domali, E	O26
De Souza Amado, S	P24	Douligeris, A	P10
De Souza Amado, S	P16	Drejza, M	O30, O67
Dedes, I	P71	Drever, N	O04
Deegan, N	O58, P15	Drosdzol-Cop, A	P75
Defert, C	O07, O12	Dumitrescu, C	P27
Dekker, J	O02	Duque Bessa, M	P16
Deleforterie, T	O07	Dural, O	O11, O25, O34, O60, O68
Deligeoroglou, E	P47, P50	Dzhafarova, M	O27, O55
**E**			
Elhasid, R	P34	Evruke, I	O34, O68
Eustaquio-David, R	P25	Evruke, I	O11, O25, O60
**F**			
Fambrini, M	O37	Foidart, J	O29, O63
Fanaki, M	P04	Foran, T	O64
Faraj, S	O12	Fouka, A	P51
Felberg, K	P05	Francesca, M	P44
Ferrari, D	P24	François-Coridon, H	O07
Filippova, E	O21	Fruzzetti, F	O29, O63
Florea, M	O47, O62	Fussner, L	P13
**G**			
Gaikaiwari, R	O04	Glenisson, M	O07
Gastol, P	P05	Gobbi Amorim, F	O63
Gavrilova, I	O05, O24	Gonçalves Vieira, L	P08
Gemzell-Danielsson, K	O28, O29	Gonçalves Vieira, L	P24
Gemzell-Danielsson, K	O63	Grajkowska, W	P05
Georgaki, M	P68	Grapsas, P	O14
Georgiou, I	O38	Grigoriadis, T	P72
Georgopapadakos, N	P61	Grimbizis, G	O19, O36, P48, P49, P50
Georgopoulos, N	O10	Grimbizis, G	O39, P47
Geramani, E	O70	Grover, S	O04, O64
Gherlan, I	P27	Grozou, F	O33, O44, O51
Giannouli, A	P77	Gruber, N	O42, O57
Gkrozou, F	O17, O31, O52, P33, P35, P71, P76	Gruca-Stryjak, K	P43
Gkrozou, F	O13	Gungor Ugurlucan, F	O11, O34
Gkyrti, G	P57, P67	Gungor Ugurlucan, F	O68
Gleeson, H	O54	Gurevich, S	P20
**H**			
Haffreingue, A	O07	Hild, O	O07
Haldre, K	O28, O29	Hilyard, T	O66
Halkia, A	O50	Hourvitz, A	O42
Hamblin, R	P70	Hristara-Papadopoulou, A	O50
Haraux, E	O07	Huirne, J	P22
Harel, Y	P20	Huirne, J	O16
**I**			
Igbokwe, R	O54	Ip, P	P09
Iglesias Rosa, E	P08, P24	Ivanets, T	P60
Impastato, G	O37, O40	Ivanidou, S	O70
Iordanidou, E	O19, O36, O39, P47, P48, P50	Ivanova, T	O24
Iordanidou, E	P49	Ivanova Panova, D	P74
Ip, N	P07	Ivanova-Panova, D	O20
Ip, P	P36		
**J**			
Jakimovska Stefanovska, M	O08, P21	Jovanovska, V	P74
Jarząbek-Bielecka, G	O67		
**K**			
Kalampalikis, A	O69	Klonos, E	O19
Kalavas, P	O17	Kokhreidze, N	P65
Kaliatsi, E	O32	Konidari, M	P39, P40
Kapczuk, K	O43, P12	Konstantinidis, I	P68
Karabatch, V	O21	Kostomoiris, G	P54, P57, P59
Karachrysafi, S	O48	Kostomoiris, G	P63
Karampas, G	P57	Kostukov, K	O21
Karkalemis, K	P56	Kotanidou, S	P18, P19
Karkalemis, K	P58	Koukoubanis, K	P78
Karopoulou, E	P61	Koukoumpanis, K	P10
Karopoulou, E	P56	Koutalia, N	P35
Kasioni, S	O26, O53	Koutrouli, M	O32
Kathopooulis, N	P78	Koutsoukou, E	O69
Kathopoulis, N	P10	Kouzouna, S	O52
Katifori, K	O17	Kowalczyk, K	P75
Katsaka, A	P54	Kravvaritis, S	P51
Katsaka, A	P59	Kristesashvili, J	O15
Katsikas, K	P76	Kritsotaki, N	P18, P19
Katsos, K	O69	Kruglyak, D	O06, O21
Kaviladze, M	O27, O55	Krzyścin, M	P43
Kavvadas, D	P68	Kucuck, S	O25
Kebudi, R	O25	Kuehne, A	P13
Kedem, A	O42	Kulabuchova, P	O06
Keramas, A	P68	Kumykova, Z	O06, O21, P60
Keskesiadou, G	O48	Kunič Pirš, T	O08, P21
Kessler, J	P60	Kwaśniak, K	O43
Khong, J	P17	Kyriakidou, M	O50
Kioussis, G	O36	Kyriakou, D	P18, P19
Klieger, C	P29, P34		
**L**			
Latthe, P	O54	Levin, I	P29
Lawson, A	O66	Leziak, A	P75
Lazaridis, A	O35, P63	Lim, L	O65
Lee, M	P07	LIM, L	O45
Lee, N	P36	Livrinova, V	O20
Lee, N	P09	Loh, S	P17
Lee, K	P36	Loh Sweet Yi, E	O65
Lefébure, C	O07	López Sanclemente, M	P28
Lentzaris, D	O31	Lubet, A	O07
Lepore, C	P13, P42	Lucchetti, M	O22
Levakov, S	O27, O55	Lynn, A	P13
Levi, L	O56		
**Ł**			
Łopiński, G	O67		
**M**			
Machairiotis, N	P11	Mertino, P	O37, O40
Madigan, G	P15	Messelod, C	O07
Magnaeva, A	P60	Metzger, R	O03
Majcherek, E	O67	Michala, L	O09, O10, O18, O26, O46, O53, O69, P04, P10, P32, P41, P52, P64, P72, P73
Makedona, C	O09	Michala, L	O70
Mamedova, F	O06, O21	Michala, L	O32, P78
Mamopoulos, A	P37	Michala, L	P11
Marasioni, M	O14, P54, P55, P59, P61, P67	Miclette, K	P13, P42
Marcos Puig, B	P26	Mikos, T	P50
Marinescu, M	P27	Mikos, T	O36
Markantonis, S	P77	Mili, S	P73
Marks, P	O54	Mirochnik, I	O05
Marogianni, A	O46	Mizrahi Sapir, R	O42
Marogianni, A	P11	Mohd Sakri, N	P30
Marozas, R	O59, O61	Monterrey Perera, V	P26
Matonóg, A	P75	Morfiadaki, A	P56, P58
Matsas, A	P53, P55	Mouchterem, A	P18
Matsas, A	P63	Moulias, E	P35
Matusiak, K	O47, O62	Moulopoulos, L	P39, P40
McDonough, R	O66	Moussaoui, D	O64, P14
McQuillan, S	O01	Mpagiasta, A	O35
Meirow, D	O56	Mytari, E	P76
**N**			
Nalmpanti, T	P18, P19	Nikolettos, K	P18, P19
Nelson, K	O01	Nikolettos, N	P18, P19
Ng, K	P07, P09	Nikolis, A	P71
Ng, K	P36	Ntali, G	P11
Nik Mhd. Nor, N	O65	Nur Azurah, A	O45
Nik Mohd Nor, N	O45		
**O**			
Ofir, K	P20	Omar, N	P38
Oikonomou, E	P18, P19	Ondrova, D	P66
Olland, A	O07	O’Neill, N	P13
Omar, N	P17	Orendi, I	O03
Omar, N	O65	Orvieto, R	O56
Omar, N	P31	Osborne, C	O01
**Ö**			
Özcan, H	O16, P22		
**P**			
Pampaloni, F	O37, O40	Pasxopoulos, M	P33
Pana, A	O19, O36, O39, P48, P49, P50	Patroni, A	O52
Pana, A	P47	Pawłowski, P	O49
Panagiotopoulos, M	O26, P10, P78	Penekeli, D	O13
Panagodimou, E	P67	Perea Monteiro, I	P16
Panagodimou, E	P58	Peterek, R	P75
Panek, G	P05	Petraglia, F	O40
Panos, A	O69	Peute, L	O16
Papadopoulou, K	P68	Piltonen, T	O29, O63
Papadopoulou, O	O50	Pinhas-Hamiel, O	O42
Papadopoulou, P	P01, P02	Pogadayeva, N	O24
Papailiou, A	P32	Politis, M	O33
Papailiou, A	P11	Poniros, N	O18
Papaioannou, G	P41	Porfyri, G	P01, P02
Papamitsou, T	O48, P68	Porfyris, K	P02
Papanikolaou, A	O38	Priebe, A	O66
Papari, N	O13	Procopiuc, C	P27
Pappa, A	O51	Pronicki, M	P05
Paschalidou, E	O14, P53	Protopapas, A	P10
Paschalidou, E	P63	Protopapas, A	P78
Paschopoulos, M	O17, O31, O33, O38, O44, P35, P45, P71	Psarris, A	P32
Paschopoulou, K	P45		
**R**			
Raanani, H	O42	Roidi, S	O70
Rabinovitch, O	P20	Roidi, S	P11
Regendova, A	P66	Rosa de Carvalho, G	P24
Reicher, L	P29, P34	Rosenberg Friedman, M	P34
Ribaldone, C	O22	Rosenberg Friedman, M	P29
Ribeiro Simões, M	P24	Rouvali, A	P41
Ribeiro Simões, M	P08, P16	Rouvali, A	O70
Richards, S	O64	Rowe, E	P13
Roidi, S	O10	Rutynowska-Pronicka, O	P05
Roidi, S	O18	Rylewicz, K	O30, O67
**S**			
Safrai, M	O56	Simões Silveira, M	P08, P24
Sagnella, L	P42	Skentou, C	O17, O33, O44, O51, O52, P71
Salisbury, E	O58	Skentou, C	O13
Samara, A	P46	Skentou, C	O31, P35
Sánchez Prieto, M	P28	Skentou, H	P76
Sánchez Borrego, R	P28	Skentou, X	P33
Sarieva, A	P49	Smith, A	P42
Saygili, H	O11, O25, O34, O60	Soldatou, A	O10, O18, O69
Saygili, H	O68	Soriano, J	P25
Scalabre, A	O07	Sosoni, A	P33
Schmitt, F	O07	Sotiriadis, A	P37
Segev, M	O42	Sotiriou, S	P46
Segev-Becker, A	P29, P34	Sowińska-Przepiera, E	P43
Serbis, A	O38	Spagnol, L	O22
Shani, H	O42	Spanakis, V	P18, P19
Shapira, M	O56	Spiliopoulou, C	O69
Shaqollari, J	O09	Stamouli, D	P46
Shemesh-iron, M	O57	Stankovic, Z	P23, P74
Siafaka, V	P45	Stavropoulou, M	O50
Sidiropoulou, P	P77	Stefanaki, C	P77
Silveri, M	O22	Strachan, B	P70
Silvi, C	P44	Sukhikh, G	O21
Sima, A	P74	Sweet Yi, E	O45
Simeonova-Krstevska, S	O20		
**s**			
Sima, a	O20		
**Š**			
Štolfa, A	O08	Štolfa Gruntar, A	P21
**T**			
Tan, A	O54	Tsai, S	O66
Tas, I	O11, O68	Tsamis, S	O51
Tas, I	O34	Tsapadikou, V	P51
Tassi, A	O22	Tsarna, E	O14, O35, P53, P54, P55, P56, P57, P58, P59, P61, P63, P67
Tatarchuk, T	O05	Tsiapakidou, S	P46
Theodora, M	P32	Tsiapakidou, S	O19, O36, O39
Theodoridis, T	P37	Tsiaras, K	O38
Theodoridis, T	O19, O36, O39, P47, P48, P49, P50	Tsikouras, P	P18, P19
Theodoridou, S	P37	Tsimaris, P	O10
Theodoulidis, I	P49	Tsiriva, M	O26, O46, O53, P10, P52, P78
Todic, I	P23	Tsiriva, M	O70
Tomic, B	P23	Tsiriva, M	P11
Torres, A	O49	Tsokkou, S	O48, P68
Toutouza, O	P62	Tsolakidis, D	P48
Triantafyllidi, V	O26, O46, P11, P72	Tsolakidis, D	P49
Triantafyllidou, O	O35, P53, P55	Tzounakou, L	P64
**U**			
Ulusoy, H	O11, O25, O34, O60, O68	Uvarova, E	O06, O21, P60
**V**			
Valero Domínguez, F	P26	Vlachos, N	P64
Valla, A	P54, P59	Vlahos, N	O35, P53, P56, P57, P67
Van den Boogaard, E	O02	Vlahos, N	P58
Vangelisti, M	O37	Vlahos, N	P39, P40, P55
Varvarousi, G	P11	Vodopivec, S	O08
Vash-Margita, A	P13	Vogiatzi, L	O70
Vash-Margita, A	P42	Vogiatzi Vokotopoulou, L	O26
Vasilopoulou, V	O18	Vogiatzi-Vokotopoulou, L	O53, P10, P52
Vatopoulou, A	O10, O17, O31, O33, O38, O44, P33, P35, P45	Vogiatzi-Vokotopoulou, L	P78
Vatopoulou, A	O13, O51, O52, P71, P76	Vozaiti, N	P52
Verot, P	O07	Vyzantiadis, A	P37
**W**			
Walewska-Wolf, M	O47	Warreth, N	O01
Walewska-Wolf, M	O62, P05	Wright, I	O04
Wang, M	P42		
**X**			
Xinias, I	O50		
**Y**			
Yarde, F	O02	Yeung, Y	P09
Yarde, F	P22	Yew Kong, L	P38
Yaron, M	P14	Yildirim, U	O25
Yasa, C	O11, O34, O68	Yildiz, M	O60
Yermyrza, A	P69		
**Z**			
Zacharakis, D	P72	Zepiridis, L	P47
Zacharis, K	P51	Ziadeh, H	P06
Zainon, N	P30	Zikopoulos, A	O13
Zainuddin, A	O65, P17, P31, P38	Zislin, M	O07
Zainuddin, A	O45	Zolota, V	P72
Zajączkowska, W	O43	Zovnir, V	O24
Zamani, M	P13	Zulkifli, Z	O65
Zanon, M	O37, O40		

